# Estimating Mixed Memberships in Directed Networks by Spectral Clustering

**DOI:** 10.3390/e25020345

**Published:** 2023-02-13

**Authors:** Huan Qing

**Affiliations:** School of Mathematics, China University of Mining and Technology, Xuzhou 221116, China; qinghuan@cumt.edu.cn or qinghuan07131995@163.com

**Keywords:** community detection, directed networks, overlapping networks, spectral clustering

## Abstract

Community detection is an important and powerful way to understand the latent structure of complex networks in social network analysis. This paper considers the problem of estimating community memberships of nodes in a directed network, where a node may belong to multiple communities. For such a directed network, existing models either assume that each node belongs solely to one community or ignore variation in node degree. Here, a directed degree corrected mixed membership (DiDCMM) model is proposed by considering degree heterogeneity. An efficient spectral clustering algorithm with a theoretical guarantee of consistent estimation is designed to fit DiDCMM. We apply our algorithm to a small scale of computer-generated directed networks and several real-world directed networks.

## 1. Introduction

Many real-world complex networks have community structure such that nodes within the same community (also known as cluster or module) have more links than across communities. For example, in social networks, communities can be groups of students in the same department; in co-authorship networks, a community can be formed by researchers in the same field. However, community structure for a real-world network is usually not directly observable. To process this problem, community detection, also known as graph clustering, is a popular tool for uncovering a latent community structure in a network [1,2]. For decades, many community detection methods have been proposed for non-overlapping undirected networks in which each node belongs to a single community, and the interactions between two nodes are symmetric or undirected. The stochastic block model (SBM) [3] is a popular generative model for non-overlapping undirected networks. In SBM, it is assumed that each node only belongs to one community and that nodes in the same community have the same expectation degrees. Ref. [4] proposes the classical degree corrected stochastic block model (DCSBM) which extends SBM by considering variation in node degree. In recent years, numerous algorithms have been developed to estimate node community for non-overlapping undirected networks generated from SBM and DCSBM, see [5,6,7,8,9,10,11,12,13,14,15]. For recent developments about SBM, see the wonderful review paper [16].

However, in most real-world networks, a node may belong to more than one community at a time. In recent years, the problem of estimating mixed memberships for the undirected network has received a lot of attention [17,18,19,20,21,22,23,24,25,26,27,28,29], and references therein. Ref. [17] extends the SBM model from non-overlapping undirected networks to mixed membership undirected networks and designs the mixed membership stochastic block (MMSB) model. Based on the MMSB model, ref. [24] designs a model called the degree corrected mixed membership (DCMM) model by considering degree heterogeneity, where DCMM can also be seen as an extension of the non-overlapping model DCSBM, and ref. [24] also develops an efficient and provably consistent spectral algorithm. Ref. [27] presents a spectral algorithm under MMSB and establishes per-node rates for mixed memberships by sharp row-wise eigenvector deviation. Ref. [29] proposes an overlapping continuous community assignment model (OCCAM), which is also an extension of MMSB, by considering degree heterogeneity. To fit OCCAM, ref. [29] develops a spectral algorithm requiring a relatively small fraction of mixed nodes when building theoretical frameworks. Ref. [26] finds the cone structure inherent in the normalization of the eigen-decomposition of the population adjacency matrix under DCMM and develops a spectral algorithm to hunt corners in the cone structure.

Though the above works are encouraging and appealing, they focus on undirected networks. In reality, there exist substantial directed networks, such as citation networks, protein–protein interaction networks, and the hyperlink network of websites. In recent years, a lot of works with encouraging results have been developed for directed networks. Ref. [30] proposes a stochastic co-block model (ScBM) and its extension DC-ScBM by considering degree heterogeneity to model non-overlapping directed networks, where ScBM and DC-ScBM can model directed networks whose row nodes may be different from column nodes, and the number of row communities may also be different from the number of column communities. Ref. [31] studies the theoretical guarantees for the algorithm DSCORE [32] and its variants designed under DC-ScBM. Ref. [33] studies the spectral clustering algorithms designed by a data-driven regularization of the adjacency matrix under ScBM. Ref. [34] studies higher-order spectral clustering of directed graphs by designing a nearly linear time algorithm. Based on the fact that the above works only consider non-overlapping directed networks, ref. [35] develops a directed mixed membership stochastic block model (DiMMSB), which is an extension of ScBM, and models directed networks with mixed memberships. DiMMSB can also be seen as a direct extension of MMSB from an undirected network to a directed network.

Recall that DCSBM, DCMM, and DCScBM are extensions of SBM, MMSB, and ScBM by considering node degree variation, respectively, this paper aims at proposing a model as an extension of DiMMSB by considering node degree heterogeneity and building an efficient spectral algorithm to fit the proposed model. In this paper, we focus on the directed network with mixed membership. Our contributions are as follows:

(i) We propose a novel generative model for directed networks with a mixed membership, the directed degree corrected mixed membership (DiDCMM) model. DiDCMM models a directed network with mixed memberships when row nodes have degree heterogeneities, while column nodes do not. We present the identifiability of DiDCMM under popular conditions which are also required by models modeling mixed membership networks when considering degree heterogeneity. Meanwhile, our results also show that modeling a directed network with mixed membership when considering degree heterogeneity for both row and column nodes needs nontrivial conditions. DiDCMM can be seen as an extension of the DCScBM model from a non-overlapping directed network to an overlapping directed network. DiDCMM also extends the DCMM model from an undirected network to a directed network and extends the DiMMSB model by considering node degree heterogeneity. For a detailed comparison of our DiDCMM with previous models, see Remark 2.
(ii) To fit DiDCMM, we present a spectral algorithm called DiMSC, which is designed based on the investigation that there exists an ideal cone structure inherent in the normalized version of the left singular vectors and an ideal simplex structure inherent in the right singular vectors of the population adjacency matrix. We prove that our DiMSC exactly recovers the membership matrices for both row and column nodes in the oracle case under DiDCMM, and this also supports the identifiability of DiDCMM. We obtain the upper bounds of error rates for each row (and column) node and show that our method produces asymptotically consistent parameter estimations under mild conditions. Our theoretical results are consistent with classical results when DiDCMM degenerates to SBM and MMSB under mild conditions. Numerical results of simulated directed networks support our theoretical results and show that our approach outperforms its competitors. We also apply our algorithm to several real-world directed networks to test the existence of highly mixed nodes and asymmetric structures between row and column communities.

**Notations.**  We take the following general notations in this paper. For a vector *x* and fixed q>0, ${∥x∥}_{q}$ denotes its $l_{q}$-norm. For a matrix *M*, $M^{\prime }$ denotes the transpose of the matrix *M*, ∥M∥ denotes the spectral norm, ${∥M∥}_{F}$ denotes the Frobenius norm, and ${∥M∥}_{2\to \infty }$ denotes the maximum $l_{2}$-norm of all the rows of *M*. Let rank(M) denote the rank of matrix *M*. Let $\sigma_{i}\left(M\right)$ be the *i*-th largest singular value of matrix *M*, and $\lambda_{i}\left(M\right)$ denote the *i*-th largest eigenvalue of the matrix *M* ordered by the magnitude. M(i,:) and M(:,j) denote the *i*-th row and the *j*-th column of matrix *M*, respectively. $M(S_{r},:)$ and $M(:,S_{c})$ denote the rows and columns in the index sets $S_{r}$ and $S_{c}$ of matrix *M*, respectively. For any matrix *M*, we simply use Y=max(0,M) to represent $Y_{ij}=\max\left(0,M_{ij}\right)$ for any i,j. For any matrix $M\in R^{m\times m}$, let diag(M) be the m×m diagonal matrix whose *i*-th diagonal entry is M(i,i). Here, 1 and 0 are column vectors with all entries being ones and zeros, respectively; $e_{i}$ is a column vector whose *i*-th entry is one, while other entries are zero. *C* is a positive constant that may vary occasionally.

## 2. The Directed Degree Corrected Mixed Membership Model

**Figure 1:**
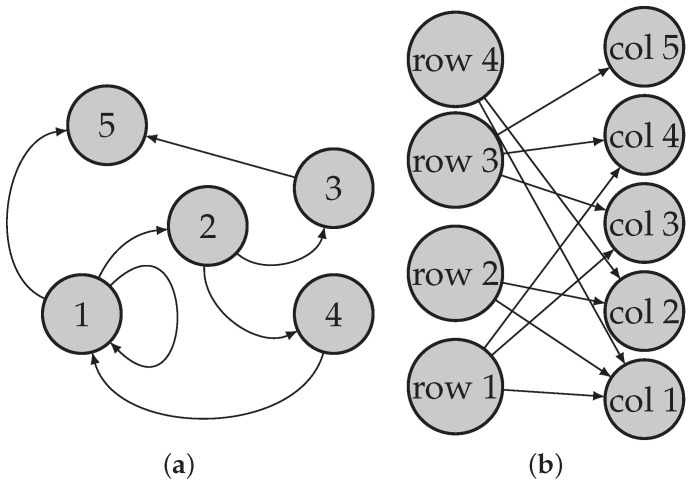
Illustration for directed network and bipartite network. Panel (**a**): directed network; Panel (**b**): bipartite network.

Consider a directed network $N=(V_{r},V_{c},E)$, where $V_{r}=\left\{1,2,\ldots ,n_{r}\right\}$ is the set of row nodes, $V_{c}=\left\{1,2,\ldots ,n_{c}\right\}$ is the set of column nodes ($n_{r}$ and $n_{c}$ indicate the number of row nodes and the number of column nodes, respectively), and E is the set of edges. Note that when $V_{r}=V_{c}$ such that row nodes are the same as column nodes, N is a traditional directed network [31,36,37,38,39,40,41,42]; when $V_{r}\neq V_{c}$, N is a bipartite network (also known as a bipartite graph) [30,33,35,43,44,45]; see Figure 1 for illustrations of the topological structures for a directed network and a bipartite network. Without confusion, we also call bipartite networks directed networks occasionally in this paper.

We assume that the row nodes of the directed network N belong to *K* perceivable communities (called row communities in this paper)

(1) $$\begin{array}{c} C_{r}^{\left(1\right)},C_{r}^{\left(2\right)},\ldots ,C_{r}^{\left(K\right)}, \end{array}$$

and the column nodes of the directed network N belong to *K* perceivable communities (called column communities in this paper)

(2) $$\begin{array}{c} C_{c}^{\left(1\right)},C_{c}^{\left(2\right)},\ldots ,C_{c}^{\left(K\right)}. \end{array}$$

Define an $n_{r}\times K$ row nodes membership matrix $\Pi_{r}$ and an $n_{c}\times K$ column nodes membership matrix $\Pi_{c}$ such that $\Pi_{r}\left(i,:\right)$ is a 1×K probability mass function (PMF) for row node *i*, $\Pi_{c}\left(j,:\right)$ is a 1×K PMF for column node *j*, and

(3) $$\begin{array}{cc} \; & \Pi_{r}\left(i,k\right)\;\mathrm{is}\;\mathrm{the}\;\mathrm{weight}\;\mathrm{of}\;\mathrm{row}\;\mathrm{node}\;i\;\mathrm{on}\;C_{r}^{\left(k\right)},1\leq k\leq K, \end{array}$$

(4) $$\begin{array}{cc} \; & \Pi_{c}\left(j,k\right)\;\mathrm{is}\;\mathrm{the}\;\mathrm{weight}\;\mathrm{of}\;\mathrm{column}\;\mathrm{node}\;j\;\mathrm{on}\;C_{c}^{\left(k\right)},1\leq k\leq K. \end{array}$$

Call row node *i* ‘pure’ if $\Pi_{r}\left(i,:\right)$ is degenerate (i.e., one entry is 1, all other K−1 entries are 0) and ‘mixed’ otherwise. The same definitions hold for column nodes. Note that mixed nodes considered in this article are not the boundary nodes introduced in [46] since boundary nodes are defined based on non-overlapping networks, while mixed nodes belong to multiple communities.

Let $A\in {\left\{0,1\right\}}^{n_{r}\times n_{c}}$ be the bi-adjacency matrix of N such that for each entry, A(i,j)=1 if there is a directional edge from row node *i* to column node *j*, and A(i,j)=0 otherwise. So, the *i*-th row of *A* records how row node *i* sends edges, and the *j*-th column of *A* records how column node *j* receives edges. Let *P* be a K×K matrix such that

(5) $$\begin{array}{c} P(k,l)\geq 0\;\mathrm{for}\;1\leq k,l\leq K. \end{array}$$

Note that since we consider a directed network in this paper, *P* may be asymmetric.

Without loss of generality, suppose that row nodes have degree heterogeneities, while column nodes do not i.e., row nodes have variation in degree, while column nodes do not. Note that in a directed network, if column nodes have degree heterogeneities while row nodes do not, to detect memberships of both row nodes and column nodes, we set the transpose of the adjacency matrix as input when applying our algorithm DiMSC. Meanwhile, in a directed network, if both row and column nodes have degree heterogeneity, to model such a directed network with mixed memberships, we need nontrivial constraints on the degree heterogeneities between row nodes and column nodes for model identifiability, for detail, see Remark 1.

Let $\theta_{r}$ be an $n_{r}\times 1$ vector whose *i*-th entry is the positive degree heterogeneity of row node *i*. For all pairs of (i,j) with $1\leq i\leq n_{r},1\leq j\leq n_{c}$, DiDCMM models the entries of *A* such that A(i,j) are independent Bernoulli random variables satisfying

(6) $$\begin{array}{c} P\left(A\left(i,j\right)=1\right)=\theta_{r}\left(i\right)\sum_{k=1}^{K}\sum_{l=1}^{K}\Pi_{r}\left(i,k\right)\Pi_{c}\left(j,l\right)P\left(k,l\right). \end{array}$$

Equation (6) means that $P\left(A\left(i,j\right)=1\right)=\theta_{r}\left(i\right)\Pi_{r}\left(i,:\right)P\Pi_{c}^{\prime }\left(j,:\right)$, i.e., the probability of generating a directional edge from row node *i* to column node *j* is $\theta_{r}\left(i\right)\Pi_{r}\left(i,:\right)P\Pi_{c}^{\prime }\left(j,:\right)$, and this probability is controlled by the degree heterogeneity parameter $\theta_{r}\left(i\right)$ of row node *i*, the connecting matrix *P*, and the memberships of nodes *i* and *j*. Equation (6) functions similarly to Equation (1.4) in [24], and both equations define the probability of generating an edge. For comparison, Equation (6) defines the probability of generating a directional edge under DiDCMM for a directed network, while Equation (1.4) in [24] defines the probability of generating an edge under DCMM for an undirected network, i.e., DiDCMM can be seen as an extension of DCMM from an undirected network to a directed network.

Introduce the degree heterogeneity diagonal matrix $\Theta_{r}\in R^{n_{r}\times n_{r}}$ for row nodes such that

(7) $$\begin{array}{c} \Theta_{r}\left(i,i\right)=\theta_{r}\left(i\right)\;\mathrm{for}\;1\leq i\leq n_{r}. \end{array}$$

Equation (7) uses a diagonal matrix $\Theta_{r}$ to contain all degree heterogeneities, and $\Theta_{r}$ is useful for further theoretical analysis through Equation (8).

**Definition 1.**  *Call model (1)–(6) the directed degree corrected mixed membership (DiDCMM) model, and denote it by $DiDCMM_{n_{r},n_{c}}\left(K,P,\Pi_{r},\Pi_{c},\Theta_{r}\right)$.*

The following conditions are sufficient for the identifiability of DiDCMM:

- (I1) rank(P)=K, and *P* has unit diagonals.
- (I2) There is at least one pure node for each of the *K* row and *K* column communities.

When building statistical models for a network in which nodes can belong to multiple communities, the full rank requirement of connecting matrix *P* and pure nodes condition are always necessary for model identifiability, see models for an undirected network such as MMSB considered in [23,27], DCMM considered in [24,26], and OCCAM considered in [26,29]. Meanwhile, if models modeling networks with mixed memberships consider degree heterogeneity, the unit diagonals requirement on connecting matrix *P* is also necessary for model identifiability, see the identifiability requirement of DCMM and OCCAM considered in [24,26,29]. Furthermore, based on the fact that DiDCMM, DCMM, and OCCAM can include the well-known model SBM, letting *P* have unit diagonals is not a serious problem since many wonderful works study a special case of SBM when *P* has unit diagonals and a network has *K* equal size clusters (this special case of SBM is also known as a planted partition model), see [12,47,48,49,50,51,52].

Let Ω=E[A] be the expectation of the adjacency matrix *A*. Under DiDCMM, we have

(8) $$\begin{array}{c} \Omega =\Theta_{r}\Pi_{r}P\Pi_{c}^{\prime }. \end{array}$$

We refer to Ω as the population adjacency matrix. Since $\mathrm{rank}\left(\Theta_{r}\right)=K,\mathrm{rank}\left(\Pi_{r}\right)=K,\mathrm{rank}\left(\Pi_{c}\right)=K$ and rank(P)=K by Equation (7) and Conditions (I1) and (I2), the rank of Ω is *K*. Recall that *K* is the number of communities, and it is much smaller than network size. We see that Ω has a low dimensional structure. The form of Ω given in Equation (8) is powerful to build the spectral algorithm developed in this paper to fit DiDCMM. Analyzing properties of the population adjacency matrix to build a spectral algorithm fitting statistical model is a common strategy in community detection, for example, references [24,26,27,35] also use this strategy to design their algorithms fitting DCMM, MMSB, and DiDCMM.

For 1≤k≤K, let $I_{r}^{\left(k\right)}=\left\{i\in \left\{1,2,\ldots ,n_{r}\right\}:\Pi_{r}\left(i,k\right)=1\right\}$ and $I_{c}^{\left(k\right)}=\left\{j\in \left\{1,2,\ldots ,n_{c}\right\}:\Pi_{c}\left(j,k\right)=1\right\}$. By Condition (I2), $I_{r}^{\left(k\right)}$ and $I_{c}^{\left(k\right)}$ are nonempty for all 1≤k≤K. For 1≤k≤K, select one row node from $I_{r}^{\left(k\right)}$ to construct the index set $I_{r}$, i.e., $I_{r}$ is the indices of row nodes corresponding to *K* pure row nodes, one from each community, and $I_{c}$ is defined similarly. W.L.O.G., let $\Pi_{r}\left(I_{r},:\right)=I_{K}$ and $\Pi_{c}\left(I_{c},:\right)=I_{K}$ (Lemma 2.1 [27] has a similar setting to design their spectral algorithm under MMSB.), where $I_{K}$ is the K×K identity matrix. The proposition below shows that the DiDCMM model is identifiable.

**Proposition 1.**  *(Identifiability). When Conditions (I1) and (I2) hold, DiDCMM is identifiable: for eligible $\left(P,\Pi_{r},\Pi_{c},\Theta_{r}\right)$ and $\left(\tilde{P},{\tilde{\Pi }}_{r},{\tilde{\Pi }}_{c},{\tilde{\Theta }}_{r}\right)$, set $\Omega =\Theta_{r}\Pi_{r}P\Pi_{c}^{\prime }$ and $\tilde{\Omega }={\tilde{\Theta }}_{r}{\tilde{\Pi }}_{r}\tilde{P}{\tilde{\Pi }}_{c}^{\prime }$. If $\Omega =\tilde{\Omega }$, then $\Theta_{r}={\tilde{\Theta }}_{r},\Pi_{r}={\tilde{\Pi }}_{r},\Pi_{c}={\tilde{\Pi }}_{c}$ and $P=\tilde{P}$.*

 **Remark** **1.** *(The reason that we do not model a directed network with mixed memberships where both row and column nodes have degree heterogeneities). Suppose both row and column nodes have degree heterogeneities in a mixed membership directed network. To model such a directed network, the probability of generating an edge from row node i to column node j is*

$$\begin{array}{c} P\left(A\left(i,j\right)=1\right)=\theta_{r}\left(i\right)\theta_{c}\left(j\right)\sum_{k=1}^{K}\sum_{l=1}^{K}\Pi_{r}\left(i,k\right)\Pi_{c}\left(j,l\right)P\left(k,l\right), \end{array}$$

*where $\theta_{c}$ is an $n_{r}\times 1$ vector whose j-th entry is the degree heterogeneity of column node j. Set Ω=E[A], then $\Omega =\Theta_{r}\Pi_{r}P\Pi_{c}^{\prime }\Theta_{c}$, where $\Theta_{c}\in R^{n_{c}\times n_{c}}$ is a diagonal matrix whose j-th diagonal entry $\theta_{c}\left(j\right)$. Set $\Omega =U\Lambda V^{\prime }$ as the compact SVD of Ω. Follow similar analysis as Lemma 1, we see that $U=\Theta_{r}\Pi_{r}B_{r}$ and $V=\Theta_{c}\Pi_{c}B_{c}$ (without causing confusion, we still use $B_{c}$ here for convenience.). For model identifiability, follow similar analysis as the proof of Proposition 1, since $\Omega \left(I_{r},I_{c}\right)=\Theta_{r}\left(I_{r},I_{r}\right)\Pi_{r}\left(I_{r},;\right)P\Pi_{c}^{\prime }\left(I_{c},:\right)\Theta_{c}\left(I_{c},I_{c}\right)=\Theta_{r}\left(I_{r},I_{r}\right)P\Theta_{c}\left(I_{c},I_{c}\right)=U\left(I_{r},:\right)\Lambda V^{\prime }\left(I_{c},:\right)$, we see that $\Theta_{r}\left(I_{r},I_{r}\right)P\Theta_{c}\left(I_{c},I_{c}\right)=U\left(I_{r},:\right)\Lambda V^{\prime }\left(I_{c},:\right)$. To obtain $\Theta_{r}\left(I_{r},I_{r}\right)$ and $\Theta_{c}\left(I_{c},I_{c}\right)$ from $U\left(I_{r},:\right)\Lambda V^{\prime }\left(I_{c},:\right)$, when P has unit diagonals, we see that it is impossible to recover $\Theta_{r}\left(I_{r},I_{r}\right)$ and $\Theta_{c}\left(I_{c},I_{c}\right)$ unless we add a condition that $\Theta_{r}\left(I_{r},I_{r}\right)=\Theta_{c}\left(I_{c},I_{c}\right)$. Now, suppose $\Theta_{r}\left(I_{r},I_{r}\right)=\Theta_{c}\left(I_{c},I_{c}\right)$ holds and call it Condition (I3); we have $\Theta_{r}\left(I_{r},I_{r}\right)P\Theta_{r}\left(I_{r},I_{r}\right)=U\left(I_{r},:\right)\Lambda V^{\prime }\left(I_{c},:\right)$; hence, $\Theta_{r}\left(I_{r},I_{r}\right)=\Theta_{c}\left(I_{c},I_{c}\right)=\sqrt{\mathrm{diag}(U\left(I_{r},:\right)\Lambda V^{\prime }\left(I_{c},:\right))}$ when P has unit diagonals. However, Condition (I3) is nontrivial since it requires $\Theta_{r}\left(I_{r},I_{r}\right)=\Theta_{c}\left(I_{c},I_{c}\right)$, and we always prefer a directed network in which there are no connections between row nodes degree heterogeneities and column nodes degree heterogeneities. For example, when all nodes are pure in a directed network, ref. [30] models such directed network using model DC-ScBM such that $\Omega =\Theta_{r}\Pi_{r}P\Pi_{c}^{\prime }\Theta_{c}$ when all nodes are pure, and $\Theta_{r}$ and $\Theta_{c}$ are independent under DC-ScBM. Because Condition (I3) is nontrivial, we do not model a mixed membership directed network with all nodes having degree heterogeneities.*

For DiDCMM’s identifiability, the number of row communities should equal that of column communities when both row and column nodes may belong to more than one community. However, when only row nodes have mixed memberships while column nodes do not, the number of row communities can be lesser than that of column communities, and this is also discussed in [53]. All proofs of our theoretical results are provided in the Appendix A.1.

Unless specified, we treat Conditions (I1) and (I2) as default from now on. Proposition 1 is important since it guarantees that our model DiDCMM is well-defined, and we can design efficient spectral algorithms to fit DiDCMM based on its identifiability. The reason that we do not consider degree heterogeneity for column nodes for our DiDCMM is mainly for its identifiability. As analyzed in Remark 1, considering degree heterogeneity for both row and column nodes make the model unidentifiable unless adding some nontrivial conditions on model parameters. Meanwhile, many previous statistical models in the community detection areas are identifiable, and spectral algorithms can be applied to fit them. For examples, SBM [3], DCSBM [4], MMSB [17], DCMM [24], OCCAM [29], ScBM (and DCScBM), [30], and DiMMSB [35] are identifiable. Especially, though different statistical models may have different requirements on model parameters for identifiability, the proof of identifiability enjoys a similar idea as that of Proposition 1, for instance, Proposition 1.1 [24] and Theorem 2.1 [27] build theoretical guarantees on identifiability for DCMM and MMSB, respectively.

**Remark 2.**  *We compare our DiDCMM with some previous models in this remark*.

- *When $\Theta_{r}=\rho I$ for ρ>0, Equation (8) gives $\Omega =\rho \Pi_{r}P\Pi_{c}^{\prime }$ and DiDCMM degenerates to DiMMSB [35], where ρ is known as a sparsity parameter [9,27,35]. So, DiDCMM includes DiMMSB as a special case, and the relationship between DiDCMM and DiMMSB is similar to that between DCSBM [3,4]. Meanwhile, DiDCMM considers degree heterogeneity parameter $\Theta_{r}$ at the cost that DiDCMM requires P to have unit diagonals for model identifiability, while there is no such requirement for P on DiMMSB’s identifiability. Note that both DiDCMM and DiMMSB are identifiable only when P is a full-rank square matrix.*
- *When $\Theta_{r}=\rho I$ for ρ>0 and all nodes are pure, DiDCMM reduces to ScBM [30]. DiDCMM can model a directed network in which nodes enjoy overlapping memberships, while ScBM cannot. Meanwhile, DiDCMM enjoys this advantage at the cost of requiring rank(P)=K for model identifiability, while ScBM is identifiable even when P is not a square matrix, i.e., ScBM can model a directed network in which the number of row communities can be different from the number of column communities. A comparison between DiDCMM and DCScBM [30] is similar.*
- *When $\Theta_{r}=\rho I$ and the network is undirected, DiDCMM reduces to MMSB [17]. However, DiDCMM models directed networks with mixed memberships, while MMSB only models undirected networks with mixed memberships. Again, DiDCMM enjoys its advantage at the cost of P having unit diagonals for its identifiability (not that DiDCMM allows P to be asymmetric since DiDCMM models directed networks), while MMSB is identifiable even when P has non-unit diagonals (note that P is symmetric under MMSB since it models undirected networks). Meanwhile, the identifiability of both DiDCMM and MMSB requires the square matrix P to have full rank.*
- *When $\Theta_{r}=\rho I$, the network is undirected and all nodes are pure, DiDCMM reduces to SBM [3]. For comparison, DiDCMM models directed networks and allows nodes to belong to multiple communities, while SBM only models undirected networks in which a node only belongs to one community. Meanwhile, DiDCMM enjoys these advantages at the cost of requiring P to be full rank with unit diagonals for its identifiability, while SBM is identifiable even when P is not full rank and P has non-unit diagonals. Note that DiDCMM allows P to be asymmetric, while P must be symmetric for SBM since DiDCMM models directed networks, while SBM models undirected networks. Comparison between DiDCMM and DCSBM [4] is similar.*
- *Compared with DCMM introduced in [24] and OCCAM introduced in [29], DCMM, and OCCAM model undirected networks with mixed memberships, while DiDCMM models directed networks with mixed memberships. DiDCMM, DCMM, and OCCAM all consider degree heterogeneity for overlapping networks, and they are identifiable only when the full rank matrix P has unit diagonals. These three models are identifiable only when the square matrix P is full rank. Meanwhile, DiDCMM allows P to be asymmetric, while P must be symmetric for DCMM and OCCAM since DiDCMM models directed networks, while DCMM and OCCAM model undirected networks.*

## 3. Algorithm

The primary goal of the proposed algorithm is to estimate the row membership matrix $\Pi_{r}$ and column membership matrix $\Pi_{c}$ from the observed adjacency matrix *A* with given *K*. We start by considering the ideal case when Ω is known, and then we extend what we learn in the ideal case to the real case.

### 3.1. The Ideal Simplex (IS), the Ideal Cone (IC), and the Ideal DiMSC

Recall that rank(Ω)=K under Conditions (I1) and (I2), and *K* is much smaller than $\min\{n_{r},n_{c}\}$. Let $\Omega =U\Lambda V^{\prime }$ be the compact singular value decomposition of Ω such that $U\in R^{n_{r}\times K},\Lambda \in R^{K\times K},V\in R^{n_{c}\times K}$, $U^{\prime }U=I_{K},V^{\prime }V=I_{K}$. The goal of the ideal case is to use U,Λ, and *V* to exactly recover $\Pi_{r}$ and $\Pi_{c}$. As stated in [8,24], $\theta_{r}$ is one of the major nuisances, and similar to [7], we remove the effect of $\theta_{r}$ by normalizing each row of *U* to have a unit $l_{2}$ norm. Set $U_{*}\in R^{n_{r}\times K}$ by $U_{*}\left(i,:\right)=\frac{U(i,:)}{{∥U\left(i,:\right)∥}_{F}}$, and let $N_{U}$ be the $n_{r}\times n_{r}$ diagonal matrix such that $N_{U}\left(i,i\right)=\frac{1}{{∥U\left(i,:\right)∥}_{F}}$ for $1\leq i\leq n_{r}$. Then, $U_{*}$ can be rewritten as $U_{*}=N_{U}U$. The existences of the ideal cone (IC for short) structure inherent in $U_{*}$ and the ideal simplex (IS for short) structure inherent in *V* are guaranteed by the following lemma.

**Lemma 1.** *(Ideal Simplex and Ideal Cone). Under $DiDCMM_{n_{r},n_{c}}\left(K,P,\Pi_{r},\Pi_{c},\Theta_{r}\right)$, there exist a unique K×K matrix $B_{r}$ and a unique K×K matrix $B_{c}$ such that*

- $U=\Theta_{r}\Pi_{r}B_{r}$, where $B_{r}=\Theta_{r}^{-1}\left(I_{r},I_{r}\right)U\left(I_{r},:\right)$, and $U_{*}=YU_{*}\left(I_{r},:\right)$ where
  $Y=N_{M}\Pi_{r}\Theta_{r}^{-1}\left(I_{r},I_{r}\right)N_{U}^{-1}\left(I_{r},I_{r}\right)$ with $N_{M}$ being an $n_{r}\times n_{r}$ diagonal matrix whose diagonal entries are positive. Meanwhile, $U_{*}\left(i,:\right)=U_{*}\left(\bar{i},:\right)$ if $\Pi_{r}\left(i,:\right)=\Pi_{r}\left(\bar{i},:\right)$ for $1\leq i,\bar{i}\leq n_{r}$.
- *$V=\Pi_{c}B_{c}$, where $B_{c}=V\left(I_{c},:\right)$. Meanwhile, $V\left(j,:\right)=V\left(\bar{j},:\right)$ if $\Pi_{c}\left(j,:\right)=\Pi_{c}\left(\bar{j},:\right)$ for $1\leq j,\bar{j}\leq n_{c}$.*

Lemma 1 says that the rows of *V* form a *K*-simplex in $R^{K}$ which we call the ideal simplex (IS), with the *K* rows of $B_{c}$ being the vertices. Such IS is also found in [24,27,35]. Lemma 1 also shows that the form of $U_{*}=YU_{*}\left(I_{r},:\right)$ is actually the ideal cone structure mentioned in [26]. Meanwhile, we remove the influence of $\theta_{r}$ by normalizing each row of *U* to have a unit norm in this paper. Using the idea of the entry-wise ratio in [8] also works, where ref. [24] develops their spectral algorithms to fit DCMM using the idea of entry-wise ratio. Designing algorithms based on the nonnegative matrix factorization [25] to fit DiDCMM by adding some constraints on Ω may also work. We leave the study of using these ideas to fit DiDCMM or its submodels for our future work.

For column nodes (recall that column nodes have no degree heterogeneities), since $B_{c}$ is full rank if *V* and $B_{c}$ are known in advance, ideally we can exactly recover $\Pi_{c}$ by setting $\Pi_{c}=VB_{c}^{\prime }{\left(B_{c}B_{c}^{\prime }\right)}^{-1}\equiv VB_{c}^{-1}$. For convenience, to transfer the ideal case to the real case, set $Z_{c}=VB_{c}^{-1}$. Since $Z_{c}\equiv \Pi_{c}$, we have

$$\begin{array}{c} \Pi_{c}\left(j,:\right)=\frac{Z_{c}\left(j,:\right)}{∥Z_{c}{\left(j,:\right)∥}_{1}},1\leq j\leq n_{c}. \end{array}$$

With given *V*, since it enjoys IS structure $V=\Pi_{c}B_{c}\equiv \Pi_{c}V\left(I_{c},:\right)$, as long as we can obtain $V(I_{c},:)$ (i.e., $B_{c}$), we can recover $\Pi_{c}$ exactly. As mentioned in [24,27], for such IS, the successive projection (SP) algorithm [54] (i.e., Algorithm A2 in the Appendix E) can be applied to *V* with *K* column communities to find the column corner matrix $B_{c}$. The above analysis gives how to recover $\Pi_{c}$ with given Ω and *K* under DiDCMM ideally.

Next, we aim to recover $\Pi_{r}$ from *U* with the given *K*. Since $\mathrm{rank}(U_{*})=K$, $\mathrm{rank}(U_{*}\left(I_{r},:\right))=K$. As $U_{*}\left(I_{r},:\right)\in R^{K\times K}$, the inverse of $U_{*}\left(I_{r},:\right)$ exists. Therefore, Lemma 1 also gives that

(9) $$\begin{array}{c} Y=U_{*}U_{*}^{-1}\left(I_{r},:\right). \end{array}$$

Equation (9) holds because $U_{*}=YU_{*}\left(I_{r},:\right)$ and $U_{*}\left(I_{r},:\right)$ is a nonsingular matrix. By Lemma 1, we know that for row nodes, their membership matrix $\Pi_{r}$ appears in the expression of *Y*. Therefore, we aim to use Equation (9) to find the exact expression of $\Pi_{r}$ using U,V, and Λ by putting *Y* at the left-hand side of equality. For our next step, we aim at finding $\Pi_{r}$ using Equation (9). Since $Y=N_{M}\Pi_{r}\Theta_{r}^{-1}\left(I_{r},I_{r}\right)N_{U}^{-1}\left(I_{r},I_{r}\right)$ by Lemma 1 and $U_{*}=N_{U}U$, using $N_{M}\Pi_{r}\Theta_{r}^{-1}\left(I_{r},I_{r}\right)N_{U}^{-1}\left(I_{r},I_{r}\right)$ and $N_{U}U$ to replace *Y* and $U_{*}$ in Equation (9), respectively, we have $N_{U}^{-1}N_{M}\Pi_{r}\Theta_{r}^{-1}\left(I_{r},I_{r}\right)N_{U}^{-1}\left(I_{r},I_{r}\right)=UU_{*}^{-1}\left(I_{r},:\right)$, which gives

(10) $$\begin{array}{c} N_{U}^{-1}N_{M}\Pi_{r}=UU_{*}^{-1}\left(I_{r},:\right)N_{U}\left(I_{r},I_{r}\right)\Theta_{r}\left(I_{r},I_{r}\right). \end{array}$$

From Equation (10), we have found the expression of $\Pi_{r}$ as a function of $U,U_{*},\Theta_{r},N_{U}$, and $I_{r}$, where we do not move $N_{U}^{-1}N_{M}$ to the right-hand side of Equation (10) because it is a diagonal matrix and does not influence the expression of $\Pi_{r}$, see our next step for details. When designing a spectral algorithm in the ideal case with given Ω and *K*, we aim at recovering $\Pi_{r}$ and $\Pi_{c}$ by taking advantage of the singular value decomposition of Ω. We find that though Equation (10) provides an expression for $\Pi_{r}$ by Ω’s SVD, there is a term $\Theta_{r}\left(I_{r},I_{r}\right)$ which relates to degree heterogeneity, and we aim at expressing $\Theta_{r}\left(I_{r},I_{r}\right)$ through Ω’s SVD. By the proof of Lemma 1, we know that $\Theta_{r}\left(I_{r},I_{r}\right)=\mathrm{diag}\left(U\left(I_{r},:\right)\Lambda V^{\prime }\left(I_{c},:\right)\right)$ when Condition (I1) holds. Thus, substituting $\mathrm{diag}(U\left(I_{r},:\right)\Lambda V^{\prime }\left(I_{c},:\right))$ for $\Theta_{r}\left(I_{r},I_{r}\right)$ in Equation (10), we obtain an expression of $\Pi_{r}$ such that this expression is directly related to Ω’s SVD and two index set $I_{r}$ and $I_{c}$. For convenience, set $J_{*}=N_{U}\left(I_{r},I_{r}\right)\Theta_{r}\left(I_{r},I_{r}\right)\equiv \mathrm{diag}\left(U_{*}\left(I_{r},:\right)\Lambda V^{\prime }\left(I_{c},:\right)\right),Z_{r}=N_{U}^{-1}N_{M}\Pi_{r},Y_{*}=UU_{*}^{-1}\left(I_{r},:\right)$. By Equation (10), we have

(11) $$\begin{array}{c} Z_{r}=Y_{*}J_{*}\equiv UU_{*}^{-1}\left(I_{r},:\right)\mathrm{diag}\left(U_{*}\left(I_{r},:\right)\Lambda V^{\prime }\left(I_{c},:\right)\right). \end{array}$$

Equation (11) looks similar to Equation (7) of [55]. However, Equation (11) is related to two index sets $I_{r}$ and $I_{c}$, while Equation (7) of [55] is only related to one index set because Equation (11) aims at designing a spectral algorithm for directed network generated under DiDCMM and Equation (7) of [55] aims at reviewing the generation of the SVM-cone-DCMMSB algorithm proposed in [26] for undirected network generated under DCMM. Meanwhile, since $N_{U}^{-1}N_{M}$ is an $n_{r}\times n_{r}$ positive diagonal matrix, we have

(12) $$\begin{array}{c} \Pi_{r}\left(i,:\right)=\frac{Z_{r}\left(i,:\right)}{∥Z_{r}{\left(i,:\right)∥}_{1}},1\leq i\leq n_{r}. \end{array}$$

With given Ω and *K*, we can obtain U,V; thus, the above analysis shows that once the two index sets $I_{r}$ and $I_{c}$ are known, we can exactly recover $\Pi_{r}$ by Equations (11) and (12). Meanwhile, from Equation (10), we see that it is important to express $\Theta_{r}\left(I_{r},I_{r}\right)$ as a combination of U,V,Λ, and the two index sets $I_{r}$ and $I_{c}$, where we successfully obtain an expression of $\Theta_{r}\left(I_{r},I_{r}\right)$ by Condition (I1), the unit diagonal constraint on *P*. Otherwise, if *P* has no unit diagonals, we cannot obtain an expression of $\Theta_{r}\left(I_{r},I_{r}\right)$ unless adding some nontrivial conditions on model parameters, just as analyzed in Remark 1. Similarly, references [24,26] also design their spectral algorithms to fit DCMM by using the unit diagonal constraint on *P* to obtain an expression of a sub-matrix of degree heterogeneity matrix, see Equations (6)–(8) of [55] as an example.

Given Ω and *K*, to recover $\Pi_{r}$ in the ideal case, we need to obtain $Z_{r}$ by Equation (11), which means that the only difficulty is in finding the index set $I_{r}$ since $V(I_{c},:)$ can be obtained by SP algorithm from the IS structure $V=\Pi_{c}V\left(I_{c},:\right)$. From Lemma 1, we know that $U_{*}=YU_{*}\left(I_{r},:\right)$ forms the IC structure. In [26], their SVM-cone algorithm (i.e., Algorithm A3 in the Appendix F) can exactly obtain the row nodes corner matrix $U_{*}\left(I_{r},:\right)$ from the ideal cone $U_{*}=YU_{*}\left(I_{r},:\right)$ as long as the Condition ${\left(U_{*}\left(I_{r},:\right)U_{*}^{\prime }\left(I_{r},:\right)\right)}^{-1}1>0$ holds (see Lemma 2).

**Lemma 2.**  Under $DiDCMM_{n_{r},n_{c}}\left(K,P,\Pi_{r},\Pi_{c},\Theta_{r}\right)$, ${\left(U_{*}\left(I_{r},:\right)U_{*}^{\prime }\left(I_{r},:\right)\right)}^{-1}1>0$ holds.

Based on the above analysis, we are now ready to give the following four-stage algorithm which we call ideal DiMSC. Input Ω,K. Output: $\Pi_{r}$ and $\Pi_{c}$.

- Let $\Omega =U\Lambda V^{\prime }$ be the compact SVD of Ω such that $U\in R^{n_{r}\times K},V\in R^{n_{c}\times K},\Lambda \in R^{K\times K},U^{\prime }U=I,V^{\prime }V=I$. Let $U_{*}=N_{U}U$, where $N_{U}$ is an $n_{r}\times n_{r}$ diagonal matrix whose *i*-th diagonal entry is $\frac{1}{{∥U\left(i,:\right)∥}_{F}}$ for $1\leq i\leq n_{r}$.
- Run the SP algorithm on *V* assuming that there are *K* column communities to obtain the column corner matrix $V(I_{c},:)$ (i.e.,$B_{c}$). Run the SVM-cone algorithm on $U_{*}$ assuming that there are *K* row communities to obtain $I_{r}$.
- Set $J_{*}=\mathrm{diag}\left(U_{*}\left(I_{r},:\right)\Lambda V^{\prime }\left(I_{c},:\right)\right),Y_{*}=UU_{*}^{-1}\left(I_{r},:\right),Z_{r}=Y_{*}J_{*}$ and $Z_{c}=VV^{-1}\left(I_{c},:\right)$.
- Recover $\Pi_{r}$ and $\Pi_{c}$ by setting $\Pi_{r}\left(i,:\right)=\frac{Z_{r}\left(i,:\right)}{∥Z_{r}{\left(i,:\right)∥}_{1}}$ for $1\leq i\leq n_{r}$, and $\Pi_{c}\left(j,:\right)=\frac{Z_{c}\left(j,:\right)}{∥Z_{c}{\left(j,:\right)∥}_{1}}$ for $1\leq j\leq n_{c}$.

The following theorem guarantees that ideal DiMSC exactly recovers nodes memberships, and this verifies the identifiability of DiDCMM in turn. Meanwhile, it should be noted that many spectral algorithms designed to fit identifiable statistical models in the community detection area can exactly recover node memberships for the ideal case. For example, the spectral clustering for *K* many clusters algorithm addressed in [5] under SBM, the regularized spectral clustering designed in [7] under DCSBM, the SCORE algorithm designed in [8] under DCSBM, the two algorithms designed and studied in [9] under SBM and DCSBM, the RSC-τ algorithm studied in [11] under SBM, the mixed-SCORE algorithm designed in [24] under DCMM, the DI-SIM algorithm designed in [30] under DCScBM, the D-SCORE algorithm studied in [31,32] under DCScBM, the SVM-cone-DCMMSB algorithm designed in [26] under DCMM, and the SPACL algorithm designed in [27] under MMSB can exactly recover membership matrices under respective models for the ideal case by using the population adjacency matrix to replace the adjacency matrix in the input of these algorithms. The fact that ideal cases for the above spectral algorithms can return community information also supports the identifiability of the above models.

**Theorem 1.**  *Under $DiDCMM_{n_{r},n_{c}}\left(K,P,\Pi_{r},\Pi_{c},\Theta_{r}\right)$, the ideal DiMSC exactly recovers the row nodes membership matrix $\Pi_{r}$ and the column nodes membership matrix $\Pi_{c}$.*

**Figure 2:**
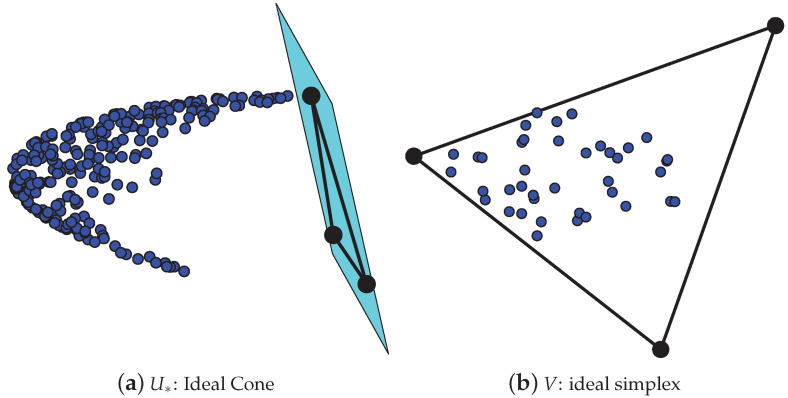
Panel (**a**): plot of $U_{*}$ and the hyperplane formed by $U_{*}\left(I_{r},:\right)$. Blue points denote rows respective to mixed row nodes of $U_{*}$, and black points denote the *K* rows of the corner matrix $U_{*}\left(I_{r},:\right)$. The plane in Panel (**a**) is the hyperplane formed by the triangle of the 3 rows of $U_{*}\left(I_{r},:\right)$. Panel (**b**): plot of *V* and the ideal simplex formed by $V(I_{c},:)$. Blue points denote rows respective to mixed column nodes of *V*, and black points denote the *K* rows of the corner matrix $V(I_{c},:)$. Since K=3, for visualization, we have projected these points from $R^{3}$ to $R^{2}$.

To demonstrate that $U_{*}$ has the ideal cone structure, we drew Panel (a) of Figure 2. The simulated data used for Panel (a) is generated from $DiDCMM_{n_{r},n_{c}}\left(K,P,\Pi_{r},\Pi_{c},\Theta_{r}\right)$ with $n_{r}=600,n_{c}=400,K=3$; each row (and column) community has 120 pure nodes. For the 240 mixed row nodes, we set $\Pi_{r}\left(i,1\right)=\mathrm{rand}\left(1\right)/2,\Pi_{r}\left(i,2\right)=\mathrm{rand}\left(1\right)/2,\Pi_{r}\left(i,3\right)=1-\Pi_{r}\left(j,1\right)-\Pi_{r}\left(j,2\right)$, where rand(1) is any random number in (0,1),

and *i* is a mixed row node. For the 40 mixed column nodes, set $\Pi_{c}\left(j,1\right)=\mathrm{rand}\left(1\right)/2$,

$\Pi_{c}\left(j,2\right)=\mathrm{rand}\left(1\right)/2,\Pi_{c}\left(j,3\right)=1-\Pi_{c}\left(j,1\right)-\Pi_{c}\left(j,2\right)$. For the degree heterogeneity parameter, set $\theta_{r}\left(i\right)=\mathrm{rand}\left(1\right)$ for all row nodes *i*. The matrix *P* is set as

$$P=\left[\begin{array}{ccc} 1 & 0.4 & 0.3\\ 0.2 & 1 & 0.1\\ 0.1 & 0.4 & 1 \end{array}\right].$$

Under such a setting, after computing Ω and obtaining $U_{*},V$ from Ω, we can plot Figure 2. Panel (a) shows that all rows respective to mixed row nodes of $U_{*}$ are located at one side of the hyperplane formed by the *K* rows of $U_{*}\left(I_{r},:\right)$, and this phenomenon occurs since each row of $U_{*}$ is a scaled convex combination of the *K* rows of $U_{*}\left(I_{r},:\right)$ guaranteed by the IC structure $U_{*}=YU_{*}\left(I_{r},;\right)$. Thus Panel (a) shows the existence of the ideal cone structure formed by $U_{*}$. Similarly, to demonstrate that *V* has the ideal simplex structure, we drew Panel (b) of Figure 2, where Panel (b) is obtained under the same setting as Panel (a). Panel (b) shows that rows respective to mixed column nodes of *V* are located inside of the simplex formed by the *K* rows of $V(I_{c},:)$, and this phenomenon occurs since each row of *V* is a convex linear combination of the *K* rows of $V(I_{c},:)$ guaranteed by the IS structure $V=\Pi_{c}V\left(I_{c},;\right)$. Thus Panel (b) shows the existence of the ideal simplex structure formed by *V*.

### 3.2. Dimsc Algorithm

We now extend the ideal case to the real case. Set $\tilde{A}=\hat{U}\hat{\Lambda }{\hat{V}}^{\prime }$ to be the top-*K*-dimensional SVD of *A* such that $\hat{U}\in R^{n_{r}\times K},\hat{V}\in R^{n_{c}\times K},\hat{\Lambda }\in R^{K\times K},{\hat{U}}^{\prime }\hat{U}=I_{K},{\hat{V}}^{\prime }\hat{V}=I_{K}$, and $\hat{\Lambda }$ contains the top *K* singular values of *A*. Let ${\hat{U}}_{*}$ be the row-wise normalization of $\hat{U}$ such that $\hat{U}=N_{\hat{U}}\hat{U}$, where $N_{\hat{U}}\in R^{n_{r}\times n_{r}}$ is a diagonal matrix whose *i*-th diagonal entry is $\frac{1}{∥\hat{U}{\left(i,:\right)∥}_{F}}$. For the real case, we use ${\hat{J}}_{*},{\hat{Y}}_{*},{\hat{Z}}_{r},{\hat{Z}}_{c},{\hat{\Pi }}_{r},{\hat{\Pi }}_{c}$ given in Algorithm 1 to estimate $J_{*},Y_{*},Z_{r},Z_{c},\Pi_{r},\Pi_{c}$, respectively. Algorithm 1 called directed mixed simplex and cone (DiMSC for short) algorithm is a natural extension of the ideal DiMSC to the real case.
**Algorithm 1:** Directed Mixed Simplex and Cone (DiMSC) algorithm**Require:** The adjacency matrix $A\in R^{n_{r}\times n_{c}}$ of a directed network, the number of row (column) communities *K*.
**Ensure:** The estimated $n_{r}\times K$ row membership matrix ${\hat{\Pi }}_{r}$ and the estimated $n_{c}\times K$ column membership matrix ${\hat{\Pi }}_{c}$.
1:Obtain $\tilde{A}=\hat{U}\hat{\Lambda }{\hat{V}}^{\prime }$, the top-*K*-dimensional SVD of *A*. Compute ${\hat{U}}_{*}$ from $\hat{U}$.2:Apply SP algorithm (i.e., Algorithm A2) on the rows of $\hat{V}$ assuming there are *K* column communities to obtain ${\hat{I}}_{c}$, the index set returned by SP algorithm.3:Similarly, apply SVM-cone algorithm (i.e., Algorithm 3) on the rows of ${\hat{U}}_{*}$ with *K* row communities to obtain ${\hat{I}}_{r}$, the index set returned by SVM-cone algorithm.4:Set ${\hat{J}}_{*}=\mathrm{diag}\left({\hat{U}}_{*}\left({\hat{I}}_{r},:\right)\hat{\Lambda }{\hat{V}}^{\prime }\left({\hat{I}}_{c},:\right)\right),{\hat{Y}}_{*}=\hat{U}{\hat{U}}_{*}^{-1}\left({\hat{I}}_{r},:\right),{\hat{Z}}_{r}={\hat{Y}}_{*}{\hat{J}}_{*}$ and ${\hat{Z}}_{c}=\hat{V}{\hat{V}}^{-1}\left({\hat{I}}_{c},:\right)$. Then, set ${\hat{Z}}_{r}=\max\left(0,{\hat{Z}}_{r}\right)$ and ${\hat{Z}}_{c}=\max\left(0,{\hat{Z}}_{c}\right)$.5:Estimate $\Pi_{r}\left(i,:\right)$ by ${\hat{\Pi }}_{r}\left(i,:\right)={\hat{Z}}_{r}\left(i,:\right)/{∥{\hat{Z}}_{r}\left(i,:\right)∥}_{1},1\leq i\leq n_{r}$ and estimate $\Pi_{c}\left(j,:\right)$ by ${\hat{\Pi }}_{c}\left(j,:\right)={\hat{Z}}_{c}\left(j,:\right)/{∥{\hat{Z}}_{c}\left(j,:\right)∥}_{1},1\leq j\leq n_{c}$.


**Figure 3:**
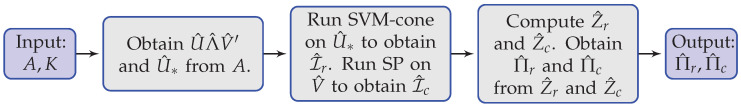
Flowchart of Algorithm 1.

In the third step, we set the negative entries of ${\hat{Z}}_{r}$ as 0 by setting ${\hat{Z}}_{r}=\max\left(0,{\hat{Z}}_{r}\right)$ for the reason that weights for any row node should be nonnegative, while there may exist some negative entries of ${\hat{Y}}_{*}{\hat{J}}_{*}$. A similar argument holds for ${\hat{Z}}_{c}$. The flowchart of DiMSC is displayed in Figure 3. Meanwhile, in community detection, researchers often use top-K-dimensional SVD of *A* or its variants such as Laplacian matrix or regularized Laplacian matrix to design their spectral clustering algorithms to fit identifiable statistical models such as spectral methods designed or studied in [5,7,8,9,11,24,26,27,29,31,33,35,56]. Furthermore, as discussed in [57], the ${\mathrm{SVS}}^{+}$ and ${\mathrm{SVS}}^{*}$ algorithms may be used as substitutions of the SP algorithm in our DiMSC for a better estimation of $\Pi_{r}$. When applying the entry-wise normalization idea developed in [8] to deal with *U*, as analyzed in [24], we obtain a simplex structure, and we can use the SP algorithm (or the combinatorial vertex search and sketched vertex search approaches developed in [24]) to hunt for the corners. The above ideas suggest that we can design different spectral algorithms to fit our model DiDCMM. We leave them for our future work. In particular, in this paper, we apply the SVM-cone algorithm to hunt for the corners of the cone structure inherent in $U_{*}$ mainly for the theoretical convenience of the SVM-cone algorithm because ref. [26] has developed a nice theoretical framework on the performance for the SVM-cone algorithm.

### 3.3. Computational Complexity

The computing cost of DiMSC mainly comes from SVD, SP, and SVM-cone. The computational complexity of SVD is $O(\max\left(n_{r},n_{c}\right)\min\left(n_{r}^{2},n_{c}^{2}\right))$. Since the adjacency matrix *A* for real-world network data sets is usually sparse, using the power method discussed in [58], the computation complexity for obtaining the top-*K*-dimensional SVD of *A* is only slightly larger than $O(\max\left(n_{r}^{2},n_{c}^{2}\right)K)$ [8,24]. The SP algorithm step in DiMSC has a complexity of $O(\max\left(n_{r},n_{c}\right)K^{2})$ [24]. The complexity of the one-class SVM step for SVM-cone algorithm is $O(\max\left(n_{r},n_{c}\right)K^{2})$ [26,59]. The complexity of the K-means step for SVM-cone algorithm is $O(\max\left(n_{r},n_{c}\right)K^{2})$ [60]. Since the number of communities *K* considered in this paper is much smaller than the network size, the total complexity of DiMSC is $O(\max\left(n_{r}^{2},n_{c}^{2}\right)K)$. Results in Section 5 show that, for a computer-generated network with 15,000 nodes under SBM, DiMSC takes hundreds of seconds to process a standard personal computer (Thinkpad X1 Carbon Gen 8) using MATLAB R2021b. Meanwhile, many spectral methods developed under models SBM, DCSBM, MMSB, ScBM, DCScBM, OCCAM, DCMM, and DiMMSB for community detection also have complexity $O(\max\left(n_{r}^{2},n_{c}^{2}\right)K)$, see spectral algorithms designed or studied in [5,7,8,9,11,24,26,27,29,30,31,33,35,61,62]. Researchers design spectral algorithms for community detection under various identifiable statistical models mainly for their convenience on building a theoretical guarantee of consistent estimation, and we also provide a theoretical guarantee on DiMSC’s estimation consistency in next section.

## 4. Consistency Results

In this section, we show the consistency of our algorithm for fitting the DiDCMM by proving that the sample-based estimates ${\hat{\Pi }}_{r}$ and ${\hat{\Pi }}_{c}$ concentrate around the true mixed membership matrices $\Pi_{r}$ and $\Pi_{c}$. Throughout this paper, *K* is a known positive integer. Set $\theta_{r,\max}=\max_{1\leq i\leq n_{r}}\theta_{r}\left(i\right)$ and $\theta_{r,\min}=\min_{1\leq i\leq n_{r}}\theta_{r}\left(i\right)$. Assume that

**Assumption 1.**  *$P_{\max}\max(∥\theta_{r}{∥}_{1},\theta_{r,\max}n_{c})\geq \log\left(n_{r}+n_{c}\right)$.*

Assumption 1 means that the network cannot be too sparse, and it also means that we allow $\theta_{r,\max}$ to go to zero with increasing numbers of row nodes and column nodes. When building theoretical guarantees on consistent estimation, controlling network sparsity is popular in the community detection area. For examples, Condition (2.9) of [8], Theorem 3.1 of [9], Condition (2.13) of [24], Assumption 3.1 of [27], and Assumption 2 of [31] all control network sparsity for their theoretical analysis. Especially, when DiDCMM reduces to SBM by letting $\Theta_{r}=\rho I,n=n_{r}=n_{c},\Pi_{r}=\Pi_{c}$, and all nodes are pure for ρ>0, Assumption A1 requires that ρn≫log(n), which is consistent with the sparsity requirement in [8,9,24,31]. As analyzed in [55], we know that our requirement on network sparsity is optimal since it matches the sharp threshold of obtaining a connected Erdös–Rényi (ER) random graph [63] when SBM reduces to an ER random graph by letting K=1.

For notation convenience, set $\varpi =\max(∥\hat{U}{\hat{U}}^{\prime }-UU^{\prime }{∥}_{2\to \infty },∥\hat{V}{\hat{V}}^{\prime }-VV^{\prime }{∥}_{2\to \infty }),{\hat{f}}_{r}$$=\max_{1\leq i\leq n_{r}}∥e_{i}^{\prime }\left({\hat{\Pi }}_{r}-\Pi_{r}P_{r}\right){∥}_{1},{\hat{f}}_{c}=\max_{1\leq j\leq n_{c}}{∥e_{j}^{\prime }\left({\hat{\Pi }}_{c}-\Pi_{c}P_{c}\right)∥}_{1}$, and $\pi_{r,\min}=\min_{1\leq k\leq K}$$1^{\prime }\Pi_{r}e_{k}$, where ϖ is the row-wise singular vector deviation which can be bounded by Theorem 4.4 of [64], ${\hat{f}}_{r}$ and ${\hat{f}}_{c}$ measures per node clustering error of DiMSC, and $\pi_{r,\min}$ measures the minimum summation of row nodes belonging to a certain row community. Increasing $\pi_{r,\min}$ makes the network tend to be more balanced and vice versa. Meanwhile, row-wise singular vector deviation is important when building a theoretical guarantee of spectral methods fitting models for a network with mixed memberships, for example, refs. [24,26,27,35] also consider ϖ when building consistent estimation for their spectral methods.

The next theorem gives theoretical bounds on estimations of memberships for both row and column nodes, which is the main theoretical result for our DiMSC method.

**Theorem 2.**  *Under $DiDCMM_{n_{r},n_{c}}\left(K,P,\Pi_{r},\Pi_{c},\Theta_{r}\right)$, let ${\hat{\Pi }}_{r}$ and ${\hat{\Pi }}_{c}$ be obtained from Algorithm 1, when Assumption 1 holds, suppose $\sigma_{K}\left(\Omega \right)\geq C\sqrt{\theta_{r,\max}P_{\max}\left(n_{r}+n_{c}\right)\log\left(n_{r}+n_{c}\right)}$, with probability at least $1-o({\left(n_{r}+n_{c}\right)}^{-3})$, we have*

$$\begin{array}{cc} \; & {\hat{f}}_{r}=O\left(\frac{K^{5.5}\theta_{r,\max}^{15}\varpi \kappa^{4.5}\left(\Pi_{r}^{\prime }\Pi_{r}\right)\kappa \left(\Pi_{c}\right)\lambda_{1}^{1.5}\left(\Pi_{r}^{\prime }\Pi_{r}\right)}{\theta_{r,\min}^{15}\pi_{r,\min}}\right),{\hat{f}}_{c}=O\left(\varpi K\kappa \left(\Pi_{c}^{\prime }\Pi_{c}\right)\sqrt{\lambda_{1}\left(\Pi_{c}^{\prime }\Pi_{c}\right)}\right). \end{array}$$

In Theorem 2, the Condition $\sigma_{K}\left(\Omega \right)\geq C\sqrt{\theta_{r,\max}P_{\max}\left(n_{r}+n_{c}\right)\log\left(n_{r}+n_{c}\right)}$ is necessary when applying Theorem 4.4 [64] to obtain a theoretical upper bound of ϖ. When building a theoretical guarantee on estimation consistency for spectral methods fitting models modeling network with mixed memberships, it is necessary to have a lower bound requirement on $\sigma_{K}\left(\Omega \right)$, see [24,26,27,35]. Actually, this requirement matches with the consistent requirement on $\frac{\sigma_{K}\left(P\right)}{\sqrt{P_{\max}}}$ obtained from the theoretical upper bound of error rates for a balanced network, see Remark 4 for details. Meanwhile, similar to [7,11,30], we can design a spectral algorithm via an application of regularized Laplacian matrix to fit DiDCMM.

The following corollary is obtained by adding conditions on model parameters similar to Corollary 3.1 in [27], where these conditions give a directed network in which each community has the same order of size, and each node has the same order of degree, i.e., a balanced network.

**Corollary 1.**  *Under $DiDCMM_{n_{r},n_{c}}\left(K,P,\Pi_{r},\Pi_{c},\Theta_{r}\right)$, when conditions of Theorem 2 hold, suppose $\lambda_{K}\left(\Pi_{r}^{\prime }\Pi_{r}\right)=O\left(\frac{n_{r}}{K}\right),\lambda_{K}\left(\Pi_{c}^{\prime }\Pi_{c}\right)=O\left(\frac{n_{c}}{K}\right),\pi_{r,\min}=O\left(\frac{n_{r}}{K}\right)$ and K=O(1), with probability at least $1-o({\left(n_{r}+n_{c}\right)}^{-3})$, we have*

$$\begin{array}{cc} \; & {\hat{f}}_{r}=O\left({\left(\frac{\theta_{r,\max}}{\theta_{r,\min}}\right)}^{15.5}\frac{1}{\sigma_{K}\left(P\right)}\sqrt{\frac{P_{\max}\log\left(n_{r}+n_{c}\right)}{\theta_{r,\min}n_{c}}}\right),{\hat{f}}_{c}=O\left({\left(\frac{\theta_{r,\max}}{\theta_{r,\min}}\right)}^{0.5}\frac{1}{\sigma_{K}\left(P\right)}\sqrt{\frac{P_{\max}\log\left(n_{r}+n_{c}\right)}{\theta_{r,\min}n_{r}}}\right). \end{array}$$

*Meanwhile,*

- *when $\theta_{r,\max}=O\left(\rho \right),\theta_{r,\min}=O\left(\rho \right)$ (i.e., $\frac{\theta_{r,\min}}{\theta_{r,\max}}=O\left(1\right)$), we have*
$$\begin{array}{cc} \; & {\hat{f}}_{r}=O\left(\frac{1}{\sigma_{K}\left(P\right)}\sqrt{\frac{P_{\max}\log\left(n_{r}+n_{c}\right)}{\rho n_{c}}}\right),{\hat{f}}_{c}=O\left(\frac{1}{\sigma_{K}\left(P\right)}\sqrt{\frac{P_{\max}\log\left(n_{r}+n_{c}\right)}{\rho n_{r}}}\right). \end{array}$$
- *when $n_{r}=O\left(n\right),n_{c}=O\left(n\right)$ and $\theta_{r,\max}=O\left(\rho \right),\theta_{r,\min}=O\left(\rho \right)$, we have*
$$\begin{array}{cc} \; & {\hat{f}}_{r}=O\left(\frac{1}{\sigma_{K}\left(P\right)}\sqrt{\frac{P_{\max}\log\left(n\right)}{\rho n}}\right),{\hat{f}}_{c}=O\left(\frac{1}{\sigma_{K}\left(P\right)}\sqrt{\frac{P_{\max}\log\left(n\right)}{\rho n}}\right). \end{array}$$

Consider a directed mixed membership network under the settings of Corollary 1 when $\theta_{r,\max}=O\left(\rho \right),\theta_{r,\min}=O\left(\rho \right)$ for ρ>0, to obtain consistent estimations for both row nodes and column nodes, by Corollary 1, $\frac{\sigma_{K}\left(P\right)}{\sqrt{P_{\max}}}$ should shrink slower than $\sqrt{\frac{\log(n_{r}+n_{c})}{\rho \min(n_{r},n_{c})}}$, where consistent estimation means that the theoretical upper bound of error rate goes to zero when increasing network size. Especially, when $n_{r}=O\left(n\right)$ and $n_{c}=O\left(n\right)$, $\frac{\sigma_{K}\left(P\right)}{\sqrt{P_{\max}}}$ should shrink slower than $\sqrt{\frac{\log(n)}{n}}$. We further assume that $P=\left(2-\beta \right)I_{K}+\left(\beta -1\right)11^{\prime }$ for β∈[1,2)∪(2,∞) and let $\tilde{P}=\rho P$ (note that for this *P*, we have $\sigma_{K}\left(P\right)=\left|\beta -2\right|$ and $P_{\max}=\max\left(1,\beta -1\right)$). So the diagonal elements for $\tilde{P}$ are ρ and non-diagonal elements are ρ(β−1). Set $p_{\mathrm{in}}$ as the diagonal entries of $\tilde{P}$, and $p_{\mathrm{out}}$ as the non-diagonal entries of $\tilde{P}$, we have $p_{\mathrm{in}}=\rho$, $p_{\mathrm{out}}=\rho \left(\beta -1\right)$, and $\frac{|p_{\mathrm{in}}-p_{\mathrm{out}}|}{\sqrt{\max(p_{\mathrm{in}},p_{\mathrm{out}})}}=\frac{\sqrt{\rho }\left|\beta -2\right|}{\sqrt{\max(1,\beta -1)}}=\frac{\sqrt{\rho }\sigma_{K}\left(P\right)}{\sqrt{P_{\max}}}$. Hence, for consistent estimation, we see that $\frac{|p_{\mathrm{in}}-p_{\mathrm{out}}|}{\sqrt{\max(p_{\mathrm{in}},p_{\mathrm{out}})}}$ should shrink slower than $\sqrt{\frac{\log(n_{r}+n_{c})}{\min(n_{r},n_{c})}}$ by Corollary 1 and should shrink slower than $\sqrt{\frac{\log(n)}{n}}$ when $n_{r}=O\left(n\right)$ and $n_{c}=O\left(n\right)$, where this result is consistent with classical separation condition for a standard network with two equal-sized clusters by applying the separation condition and sharp threshold criterion developed in [55].

**Remark 3.**  *When the network is undirected (i.e., $n_{r}=n_{c}=n,\Pi_{r}=\Pi_{c}$) with K=O(1) by setting $\theta_{r}\left(i\right)=\rho$ for $1\leq i\leq n_{r}$, DiDCMM degenerates to MMSB considered in [27], the upper bound of error rate for DiMSC is $O(\frac{1}{\sigma_{K}\left(P\right)}\sqrt{\frac{\log(n)}{\rho n}})$ when $P_{\max}=1$. Replacing the Θ in [24] by $\Theta =\sqrt{\rho }I$, their DCMM model degenerates to MMSB. Then, their conditions in Theorem 2.2 are our Assumption 1 and $\lambda_{K}\left(\Pi^{\prime }\Pi \right)=O\left(\frac{n}{K}\right)$, where $\Pi =\Pi_{r}=\Pi_{c}$ for MMSB. When K=O(1), the error bound in Theorem 2.2 in [24] is $O(\frac{1}{\sigma_{K}\left(P\right)}\sqrt{\frac{\log(n)}{\rho n}})$, which is consistent with ours.*

**Remark 4.**  *By Lemma A5 in the Appendix D, we know $\sigma_{K}\left(\Omega \right)\geq \theta_{r,\min}\sigma_{K}\left(P\right)\sigma_{K}\left(\Pi_{r}\right)\sigma_{K}\left(\Pi_{c}\right)$. To ensure the Condition $\sigma_{K}\left(\Omega \right)\geq C{\left(\theta_{r,\max}P_{\max}\left(n_{r}+n_{c}\right)\log\left(n_{r}+n_{c}\right)\right)}^{1/2}$ in Theorem 2 holds, we need*

(13) $$\begin{array}{c} \frac{\sigma_{K}\left(P\right)}{\sqrt{P_{\max}}}\geq C{\left(\frac{\theta_{r,\max}\left(n_{r}+n_{c}\right)\log\left(n_{r}+n_{c}\right)}{\theta_{r,\min}^{2}\lambda_{K}\left(\Pi_{r}^{\prime }\Pi_{r}\right)\lambda_{K}\left(\Pi_{c}^{\prime }\Pi_{c}\right)}\right)}^{1/2}. \end{array}$$

*When $K=O\left(1\right),n_{r}=O\left(n\right),n_{c}=O\left(n\right),\lambda_{K}\left(\Pi_{r}^{\prime }\Pi_{r}\right)=O\left(\frac{n_{r}}{K}\right),\lambda_{K}\left(\Pi_{c}^{\prime }\Pi_{c}\right)=O\left(\frac{n_{c}}{K}\right)$ and $\theta_{r,\max}=O\left(\rho \right),\theta_{r,\min}=O\left(\rho \right)$, Equation (13) gives that $\frac{\sigma_{K}\left(P\right)}{\sqrt{P_{\max}}}$ should shrink slower than $\sqrt{\frac{\log(n)}{\rho n}}$, which matches with the consistency requirement on $\frac{\sigma_{K}\left(P\right)}{\sqrt{P_{\max}}}$ of Corollary 1.*

For convenience, we need the following definition.

**Definition 2.**  *Let $DiDCMM(n,K,\Pi_{r},\Pi_{c},\alpha_{\mathrm{in}},\alpha_{\mathrm{out}})$ be a special case of $DiMMDF_{n_{r},n_{c}}\left(K,P,\Pi_{r},\Pi_{c},\Theta_{r}\right)$ when $\Theta_{r}=\rho I,n_{r}=n_{c}=n,\lambda_{K}\left(\Pi_{r}^{\prime }\Pi_{r}\right)=O\left(n/K\right),\lambda_{K}\left(\Pi_{c}^{\prime }\Pi_{c}\right)=O\left(n/K\right),\pi_{r,\min}=O\left(n/K\right),K=O\left(1\right)$, and $\tilde{P}=\rho P$ has diagonal entries $p_{\mathrm{in}}=\alpha_{\mathrm{in}}\frac{\log(n)}{n}$ and non-diagonal entries $p_{\mathrm{out}}=\alpha_{\mathrm{out}}\frac{\log(n)}{n}$.*

$DiDCMM(n,K,\Pi_{r},\Pi_{c},\alpha_{\mathrm{in}},\alpha_{\mathrm{out}})$ denotes a special directed network such that row communities have nearly equal sizes since $\lambda_{K}\left(\Pi_{r}^{\prime }\Pi_{r}\right)=O\left(n/K\right)$, and column communities also have nearly equal sizes. By Corollary 1, for consistent estimation, we need $\frac{|p_{\mathrm{in}}-p_{\mathrm{out}}|}{\sqrt{\max(p_{\mathrm{in}},p_{\mathrm{out}})}}\gg \sqrt{\frac{\log(n)}{n}}$ under $DiDCMM(n,K,\Pi_{r},\Pi_{c},\alpha_{\mathrm{in}},\alpha_{\mathrm{out}})$. Since $\frac{|p_{\mathrm{in}}-p_{\mathrm{out}}|}{\sqrt{\max(p_{\mathrm{in}},p_{\mathrm{out}})}}=\frac{|\alpha_{\mathrm{in}}-\alpha_{\mathrm{out}}|\sqrt{\frac{\log(n)}{n}}}{\sqrt{\max(\alpha_{\mathrm{in}},\alpha_{\mathrm{out}})}}$, for consistent estimation, we need

(14) $$\begin{array}{c} \frac{|\alpha_{\mathrm{in}}-\alpha_{\mathrm{out}}|}{\sqrt{\max(\alpha_{\mathrm{in}},\alpha_{\mathrm{out}})}}\gg 1 \end{array}$$

Our numerical results in Section 5 support that DiMSC can estimate memberships for both row and column nodes when the threshold $\frac{|\alpha_{\mathrm{in}}-\alpha_{\mathrm{out}}|}{\sqrt{\max(\alpha_{\mathrm{in}},\alpha_{\mathrm{out}})}}\gg 1$ holds under $DiDCMM(n,K,\Pi_{r},\Pi_{c},\alpha_{\mathrm{in}},\alpha_{\mathrm{out}})$.

**Remark 5.**  *When K=2, the network is undirected (i.e., $\Pi_{r}=\Pi_{c}$), all nodes are pure, and each community has an equal size, $DiDCMM(n,K,\Pi_{r},\Pi_{c},\alpha_{\mathrm{in}},\alpha_{\mathrm{out}})$ reduces to the SBM case such that nodes connect with probability $p_{\mathrm{in}}$ within clusters and $p_{\mathrm{out}}$ across clusters. This case has been well studied in recent years, see [50] and references therein. Especially, for this case, ref. [50] finds that exact recovery is possible if $|\sqrt{\alpha_{\mathrm{in}}}-\sqrt{\alpha_{\mathrm{out}}}|>\sqrt{2}$ and impossible if $|\sqrt{\alpha_{\mathrm{in}}}-\sqrt{\alpha_{\mathrm{out}}}|<\sqrt{2}$. For convenience, we use $SBM(n,p_{\mathrm{in}},p_{\mathrm{out}})$ to denote this case. Our numerical results in Section 5 show that DiMSC return consistent estimation under $SBM(n,p_{\mathrm{in}},p_{\mathrm{out}})$ when $\alpha_{\mathrm{in}}$ and $\alpha_{\mathrm{out}}$ are set in the impossible region of exact recovery but satisfy Equation (14).*

**Remark 6.**  *In information theory, Shannon entropy [65] quantifies the amount of information in a variable, and it is a measure of uncertainty information of a probability distribution. We use a node membership entropy (NME) derived from Shannon theory to measure the node’s uncertainty about the node and all communities [66,67]. For row node i with membership $\Pi_{r}\left(i,:\right)$, since $\sum_{k=1}^{K}\Pi_{r}\left(i,k\right)=1$ and $\Pi_{r}\left(i,k\right)$ can be seen as the probability that row node i belongs to row cluster k for 1≤k≤K, NME of row node i is the Shannon entropy related to $\Pi_{r}\left(i,:\right)$:*

(15) $$\begin{array}{c} NME\left(i\right)=-\sum_{k=1}^{K}\Pi_{r}\left(i,k\right)\log\left(\Pi_{r}\left(i,k\right)\right). \end{array}$$

*For column node j with membership $\Pi_{c}\left(j,:\right)$, we can also obtain its NME by Equation (15). In particular, if a node belongs to each cluster with equal probability $\frac{1}{K}$, its NME is log(K) which is the maximum among all NME; if a node belongs to two clusters with equal probability $\frac{1}{2}$, its NME is log(2) which is less than log(K) when K≥3. Generally, we see that recovering memberships for mixed nodes is harder than for pure nodes since NME is 0 for pure nodes, while NME is larger than 0 for mixed nodes by the definition of NME.*

## 5. Simulations

In this section, several experiments are conducted to investigate the performance of our DiMSC under DiDCMM. We compare our DiMSC with three model-based methods that can be thought of as special cases of our model DiDCMM. Model-based methods we compare include the DISIM algorithm proposed in [30], the DSCORE algorithm studied in [31], and the DiPCA algorithm which is obtained by using the adjacency matrix *A* to replace the regularized graph Laplacian matrix in the DISIM algorithm. Similar to [24,27], for simulations, we measure the errors for the inferred community membership matrices instead of simply each node. We measure the performance of DiMSC and its competitors by the mixed Hamming error rate (MHamm for short) defined below

(16) $$\begin{array}{cc} \; & \mathrm{MHamm}=\max(\frac{\min_{P\in S_{P}}{∥{\hat{\Pi }}_{r}P-\Pi_{r}∥}_{1}}{n_{r}},\frac{\min_{P\in S_{P}}{∥{\hat{\Pi }}_{c}P-\Pi_{c}∥}_{1}}{n_{c}}), \end{array}$$

where $S_{P}$ is the set of K×K permutation matrices.

For all simulations in this section, unless specified, we set the parameters $\left(n_{r},n_{c},K,P,\Pi_{r},\Pi_{c},\Theta_{r}\right)$ under DiDCMM as follows: let each row community and each column community have $n_{0}$ pure nodes; let all mixed row nodes (and mixed column nodes) have membership (1/K,1/K,…,1/K); for z≥1, we generate the degree parameters for row nodes as below: let ${\bar{\theta }}_{r}\in R^{n_{r}\times 1}$ such that $1/{\bar{\theta }}_{r}\left(i\right)\overset{iid}{∼}U\left(1,z\right)$ for $1\leq i\leq n_{r}$, where U(1,z) denotes the uniform distribution on [1,z], and set $\theta_{r}=\rho {\bar{\theta }}_{r}$, where we use ρ to control the sparsity of the network; when K=2, *P* is set as

$$\begin{array}{cc} P_{1} & =\left[\begin{array}{cc} 1 & 0.1\\ 0.2 & 1 \end{array}\right]\mathrm{or}\;P_{2}=\left[\begin{array}{cc} 0.8 & 0.1\\ 0.2 & 0.9 \end{array}\right]; \end{array}$$

when K=3,

$$\begin{array}{cc} P_{3} & =\left[\begin{array}{ccc} 1 & 0.1 & 0.3\\ 0.2 & 1 & 0.4\\ 0.5 & 0.2 & 1 \end{array}\right]\mathrm{or}\;P_{4}=\left[\begin{array}{ccc} 0.8 & 0.1 & 0.3\\ 0.2 & 0.9 & 0.4\\ 0.5 & 0.2 & 1 \end{array}\right]; \end{array}$$

where $P_{2}$ and $P_{4}$ have non-unit diagonals, and we consider the two cases because we want to investigate DiMSC’s sensitivity when *P* has non-unit diagonals such that *P* disobeys Condition (I1).

After obtaining $P,\Pi_{r},\Pi_{c},\theta_{r}$, similar to the five simulation steps in [8], each simulation experiment contains the following steps:

(a) Let $\Theta_{r}$ be the $n_{r}\times n_{r}$ diagonal matrix such that $\Theta_{r}\left(i,i\right)=\theta_{r}\left(i\right),1\leq i\leq n_{r}$. Set $\Omega =\Theta_{r}\Pi_{r}P\Pi_{c}^{\prime }$.

(b) Let *W* be an $n_{r}\times n_{c}$ matrix such that W(i,j) are independent centered-Bernoulli with parameters Ω(i,j). Let $\tilde{A}=\Omega +W$.

(c) Set ${\tilde{S}}_{r}=\left\{i:\sum_{j=1}^{n_{c}}\tilde{A}\left(i,j\right)=0\right\}$ and ${\tilde{S}}_{c}=\left\{j:\sum_{i=1}^{n_{r}}\tilde{A}\left(i,j\right)=0\right\}$, i.e., ${\tilde{S}}_{r}$ (${\tilde{S}}_{c}$) is the set of row (column) nodes with 0 edges. Let *A* be the adjacency matrix obtained by removing rows respective to nodes in ${\tilde{S}}_{r}$ and removing columns respective to nodes in ${\tilde{S}}_{c}$ from $\tilde{A}$. Similarly, update $\Pi_{r}$ by removing nodes in ${\tilde{S}}_{r}$ and update $\Pi_{c}$ by removing nodes in ${\tilde{S}}_{c}$.

(d) Apply the DiMSC algorithm (and its competitors) to *A*. Record MHamm under investigations.

(e) Repeat (b)–(d) 50 times, and report the averaged MHamm over the 50 repetitions.

Let $n_{r,A}$ be the number of rows of *A* and $n_{c,A}$ be the number of columns of *A*. In our experiments, $n_{r,A}$ and $n_{c,A}$ are usually very close to $n_{r}$ and $n_{c}$; therefore we do not report the exact values of $n_{r,A}$ and $n_{c,A}$. After providing the above steps about how to generate *A* numerically under DiDCMM and how to record the error rates, now we describe our experiments in detail. We consider six experiments here. In experiments 1–6, we study the influence of the fraction of pure nodes, degree heterogeneity, connectivity across communities, sparsity, phase transition, and network size on performances of these methods, respectively.

**Figure 4:**
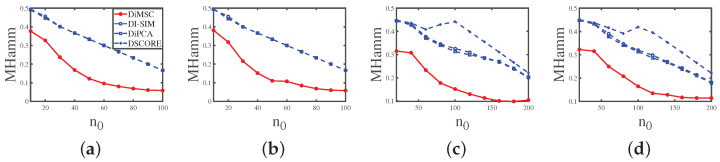
Errors against increasing $n_{0}$. y-axis: MHamm. Panel (**a**): Experiment 1 (a); Panel (**b**): Experiment 1 (b); Panel (**c**): Experiment 1 (c); Panel (**d**): Experiment 1 (d).

Experiment 1 (a): Fraction of pure nodes. Set $n_{r}=200,n_{c}=300,z=5,\rho =1$ and *P* as $P_{1}$. Let $n_{0}$ range in {10,20,30,…,100}. The numerical results are shown in Panel (a) of Figure 4. The results show that as the fraction of pure nodes increases for both row and column communities, all approaches perform better. Meanwhile, DiMSC performs best among all methods in Experiment 1 (a).

Experiment 1 (b): Fraction of pure nodes. All parameters are set the same as Experiment 1 (a) except that we set *P* as $P_{2}$ here. The numerical results are shown in Panel (b) of Figure 4. The results show that all methods perform better as $n_{0}$ increases, DiMSC outperforms its competitors, and DiMSC enjoys satisfactory performance even when *P* has non-unit diagonals.

Experiment 1 (c): Fraction of pure nodes. Set $n_{r}=600,n_{c}=900,z=5$, ρ=1, and *P* as $P_{3}$. Let $n_{0}$ range in {20,40,60,…,200}. The numerical results are shown in Panel (c) of Figure 4, and we see that all methods perform better when there are more pure nodes and our DiMSC performs best.

Experiment 1 (d): Fraction of pure nodes. All parameters are set the same as Experiment 1 (c) except that we set *P* as $P_{4}$ here. The numerical results are shown in Panel (d) of Figure 4, and the analysis is similar to that of Experiment 1 (b).

**Figure 5:**
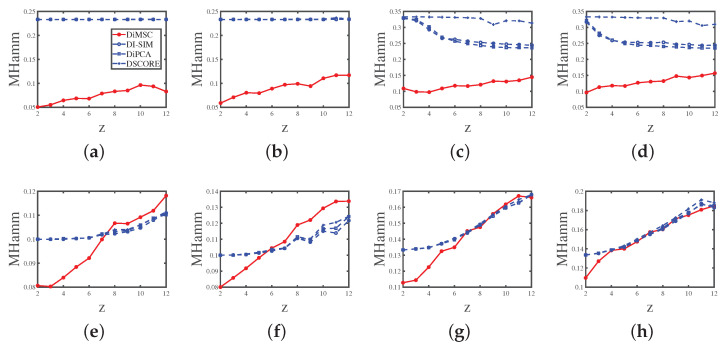
Errors against increasing *z*. *y*-axis: MHamm. Panel (**a**): Experiment 2 (a); Panel (**b**): Experiment 2 (b); Panel (**c**): Experiment 2 (c); Panel (**d**): Experiment 2 (d); Panel (**e**): Experiment 2 (e); Panel (**f**): Experiment 2 (f); Panel (**g**): Experiment 2 (g); Panel (**h**): Experiment 2 (h).

Experiment 2 (a): Degree heterogeneity. Set $n_{r}=200,n_{c}=300,n_{0}=80,\rho =1$, and *P* as $P_{1}$. Let *z* range in {2,3,4,…,12}. A lager *z* generates lesser edges. The results are displayed in Panel (a) of Figure 5. The results suggest that the error rates of DiMSC for both row and column nodes tend to increase as *z* increases. This phenomenon happens because decreasing degree heterogeneities for row nodes lowers the number of edges in the directed network; thus the network becomes harder to be detected for both row and column nodes. Meanwhile, DiMSC outperforms its competitors in this experiment, and it is interesting to see that the error rates of DI-SIM, DiPCA, and DSCORE are almost the same for this experiment.

Experiment 2 (b): Degree heterogeneity. All parameters are set the same as Experiment 2 (a) except that we set *P* as $P_{2}$ here. The results are displayed in Panel (b) of Figure 5, and we see that DiMSC performs satisfactorily when the directed network is not too sparse (i.e., a small *z* case) even when *P* has non-unit diagonals. Meanwhile, DiMSC significantly outperforms its competitors in this experiment.

Experiment 2 (c): Degree heterogeneity. Set $n_{r}=600,n_{c}=900,n_{0}=150$, ρ=1, and *P* as $P_{3}$. Let *z* range in {2,3,4,…,12}. The results are shown in Panel (c) of Figure 5 and can be analyzed similarly to Experiment 2 (a).

Experiment 2 (d): Degree heterogeneity. All parameters are set the same as Experiment 2 (c) except that we set *P* as $P_{4}$ here. The results are displayed in Panel (d) of Figure 5 and are similar to that of Experiment 2 (b).

Experiment 2 (e): Degree heterogeneity. All parameters are set the same as Experiment 2(a) except that we set $n_{0}=0$ (so there are no pure nodes in both row and column communities), and all mixed row nodes have two different memberships (0.9, 0.1) and (0.1, 0.9), each with $\frac{n_{r}}{K}=100$ number of row nodes, and all mixed column nodes also have the above two memberships, each with $\frac{n_{c}}{K}=150$ number of column nodes. Panel (e) of Figure 5 shows the results, and we see that DiMSC performs satisfactorily for a small *z* even for the case when there are no pure nodes for both row and column communities. Meanwhile, DiMSC performs better than its competitors when z<7, and it perform poorer than its competitors when z≥8 for this experiment. Furthermore, compared with numerical results of Experiment 2 (a), we see that DI-SIM, DiPCA, and DSCORE have better performances in Experiment 2 (e). The possible reason is the memberships (0.9,0.1) and (0.1,0.9) are close to (1,0) and (0,1) somewhat.

Experiment 2 (f): Degree heterogeneity. All parameters are set the same as Experiment 2 (b) except that we set $\Pi_{r}$ and $\Pi_{c}$ the same as Experiment 2 (e). The results are shown in Panel (f) of Figure 5 and are similar to that of Experiment 2 (e).

Experiment 2 (g): Degree heterogeneity. All parameters are set the same as Experiment 2 (c) except that we set $n_{0}=0$, all mixed row nodes have three different memberships (0.8, 0.1, 0.1), (0.1, 0.8, 0.1), and (0.1,0.1,0.8), each with $\frac{n_{r}}{K}=200$ number of row nodes, and all mixed column nodes also have the above four memberships, each with $\frac{n_{c}}{K}=300$ number of column nodes. The results are displayed in Panel (g) of Figure 5 and are similar to that of Experiment 2 (e).

Experiment 2 (h): Degree heterogeneity. All parameters are set the same as Experiment 2 (d) except that we set $\Pi_{r}$ and $\Pi_{c}$ the same as Experiment 2 (g). The results are shown in Panel (h) of Figure 5 and are similar to that of Experiment 2 (e).

**Figure 6:**
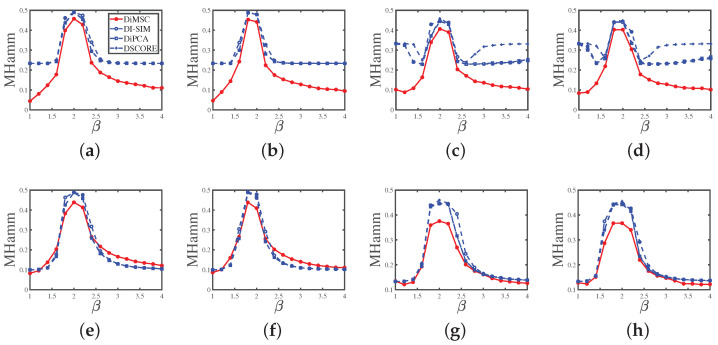
Errors against increasing β. *y*-axis: MHamm. Panel (**a**): Experiment 3 (a); Panel (**b**): Experiment 3 (b); Panel (**c**): Experiment 3 (c); Panel (**d**): Experiment 3 (d); Panel (**e**): Experiment 3 (e); Panel (**f**): Experiment 3 (f); Panel (**g**): Experiment 3 (g); Panel (**h**): Experiment 3 (h).

Experiment 3 (a): Connectivity across communities. Set $n_{r}=200,n_{c}=300$, $n_{0}=80$, z=5,ρ=1. Set

$$P=\left[\begin{array}{cc} 1 & \beta -1\\ \beta -1 & 1 \end{array}\right].$$

and let β range in {1,1.2,1.4,…,4}. Decreasing |β−2| increases the hardness of detecting such directed networks. Note that $P\left(A\left(i,j\right)=1\right)=\Omega \left(i,j\right)=\theta_{r}\left(i\right)\Pi_{r}\left(i,:\right)P\Pi_{c}^{\prime }\left(j,:\right)$ gives $\max_{i,j}\Omega \left(i,j\right)=\theta_{r,\max}P_{\max}$ should be no larger than 1. Since $P_{\max}$ may be larger than one in this experiment, after obtaining $\theta_{r}$, we need to update $\theta_{r}$ as $\theta_{r}/P_{\max}$. The results are displayed in Panel (a) of Figure 6, and they support the arguments given after Corollary 1 such that DiMSC performs better when |β−2| increases and vice versa. Meanwhile, our DiMSC outperforms its competitors in this experiment.

Experiment 3 (b): Connectivity across communities. All parameters are set the same as Experiment 3 (a) except that we set

$$P=\left[\begin{array}{cc} 0.8 & \beta -1\\ \beta -1 & 0.9 \end{array}\right].$$

The results are displayed in Panel (b) of Figure 6, and we see that DiMSC performs better when |β−2| increases even for the case that *P* has non-unit diagonals.Meanwhile, our DiMSC performs better than its competitors here.

Experiment 3 (c): Connectivity across communities. Set $n_{r}=600,n_{c}=900,n_{0}=150,z=5,\rho =1$. Set

$$P=\left[\begin{array}{ccc} 1 & \beta -1 & \beta -1\\ \beta -1 & 1 & \beta -1\\ \beta -1 & \beta -1 & 1 \end{array}\right].$$

and let β range in {1,1.2,1.4,…,4}. The results are displayed in Panel (c) of Figure 6 and can be analyzed similarly to Experiment 3 (a).

Experiment 3 (d): Connectivity across communities. All parameters are set the same as Experiment 3(c) except that we set

$$P=\left[\begin{array}{ccc} 0.8 & \beta -1 & \beta -1\\ \beta -1 & 0.9 & \beta -1\\ \beta -1 & \beta -1 & 1 \end{array}\right].$$

The results are displayed in Panel (d) of Figure 6 and can be analyzed similarly to Experiment 3 (b).

Experiment 3 (e): Connectivity across communities. All parameters are set the same as Experiment 3(a) except that we let $\Pi_{r}$ and $\Pi_{c}$ be the same as that of Experiment 2 (e) (so there are no pure nodes in both row and column communities.). Panel (e) of Figure 6 shows the results, and we see that DiMSC enjoys better performance when |β−2| increases even in the case that there are no pure nodes for both row and column communities. Meanwhile, all methods have competitive performances for this experiment, and the possible reason that DiMSC’s competitors enjoy better performances here than in Experiment 3 (a) is analyzed in Experiment 2 (e).

Experiment 3 (f): Connectivity across communities. All parameters are set the same as Experiment 3 (b) except that we set $\Pi_{r}$ and $\Pi_{c}$ the same as Experiment 2 (e). The results are displayed in Panel (f) of Figure 6 and can be analyzed similarly to Experiment 3 (e).

Experiment 3 (g): Connectivity across communities. All parameters are set the same as Experiment 3 (c) except that we let $\Pi_{r}$ and $\Pi_{c}$ be the same as that of Experiment 2 (g) (so there are no pure nodes). Panel (g) of Figure 6 shows the results, and the analysis is similar to that of Experiment 3 (b).

Experiment 3 (h): Connectivity across communities. All parameters are set the same as Experiment 3 (d) except that we set $\Pi_{r}$ and $\Pi_{c}$ the same as Experiment 2 (g). Panel (h) of Figure 6 shows the results, and the analysis is similar to that of Experiment 3 (b).

**Figure 7:**
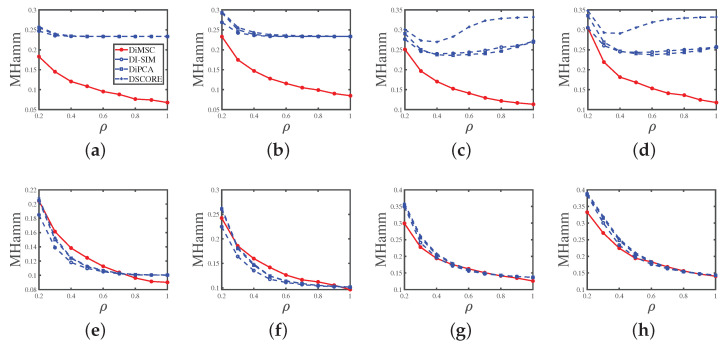
Errors against increasing ρ. *y*-axis: MHamm: Panel (**a**): Experiment 4 (a); Panel (**b**): Experiment 4 (b); Panel (**c**): Experiment 4 (c); Panel (**d**): Experiment 4 (d); Panel (**e**): Experiment 4 (e); Panel (**f**): Experiment 4 (f); Panel(**g**): Experiment 4 (g); Panel (**h**): Experiment 4 (h).

Experiment 4 (a): Sparsity. Set $n_{r}=200,n_{c}=300,n_{0}=80,z=5,$ and *P* as $P_{1}$. Let ρ range in {0.2,0.3,…,1}. A larger ρ indicates a denser network. Panel (a) in Figure 7 displays the simulation results of this experiment. We see that DiMSC performs better as the simulated directed network becomes denser, and DiMSC significantly outperforms its competitors in this experiment.

Experiment 4 (b): Sparsity. All parameters are set the same as Experiment 4 (a) except that *P* is set as $P_{2}$. Panel (b) of Figure 7 shows the results, and the analysis is similar to that of Experiment 2 (b).

Experiment 4 (c): Sparsity. Set $n_{r}=600,n_{c}=900,n_{0}=150,z=5,$ and *P* as $P_{3}$. Let ρ range in {0.2,0.3,…,1}. Panel (c) of Figure 7 shows the results, and the analysis is similar to that of Experiment 4 (a).

Experiment 4 (d): Sparsity. All parameters are set the same as Experiment 4 (c) except that *P* is set as $P_{4}$. Panel (d) of Figure 7 displays the results, and the analysis is similar to that of Experiment 4 (b).

Experiment 4 (e): Sparsity. All parameters are set the same as Experiment 4 (a) except that we let $\Pi_{r}$ and $\Pi_{c}$ be the same as that of Experiment 2 (e). Panel (e) of Figure 7 shows the results, and we see that DiMSC’s error rates decrease for a denser directed network even when all nodes are mixed. Meanwhile, all methods enjoy similar performances in this experiment.

Experiment 4 (f): Sparsity. All parameters are set the same as Experiment 4 (b) except that we set $\Pi_{r}$ and $\Pi_{c}$ the same as Experiment 2(e). Panel (f) of Figure 7 shows the results, and the analysis is similar to that of Experiment 4 (e).

Experiment 4 (g): Sparsity. All parameters are set the same as Experiment 4 (c) except that we let $\Pi_{r}$ and $\Pi_{c}$ be the same as that of Experiment 2 (g). Panel (g) of Figure 7 shows the results, and the analysis is similar to that of Experiment 4 (e).

Experiment 4 (h): Sparsity. All parameters are set the same as Experiment 4 (d) except that we set $\Pi_{r}$ and $\Pi_{c}$ the same as Experiment 2(g). Panel (h) of Figure 7 shows the results, and the analysis is similar to that of Experiment 4 (e).

**Figure 8:**
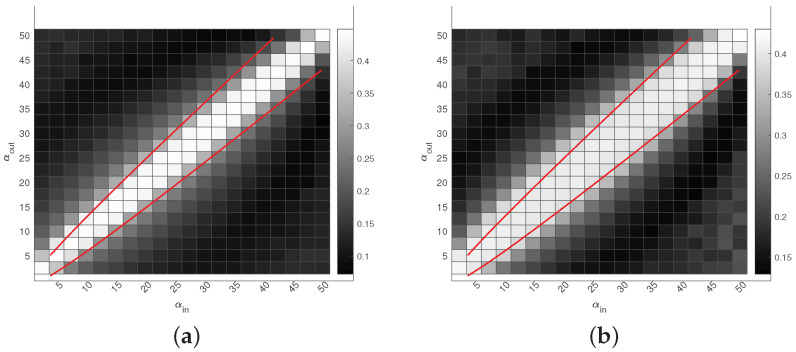
Phase transition for DiMSC: darker pixels represent lower error rates. The red lines represent $\frac{|\alpha_{\mathrm{in}}-\alpha_{\mathrm{out}}|}{\sqrt{\max(\alpha_{\mathrm{in}},\alpha_{\mathrm{out}})}}=1$. Panel (**a**): Experiment 5 (a); Panel (**b**): Experiment 5 (b).

Experiment 5 (a): Phase transition. Under $DiDCMM(n,K,\Pi_{r},\Pi_{c},\alpha_{\mathrm{in}},\alpha_{\mathrm{out}})$, set $K=2,n=n_{r}=n_{c}=300$. Let each row community have 100 pure nodes, each column community have 120 pure nodes, and all mixed nodes have membership (1/2,1/2). Since $\max\left(p_{\mathrm{in}},p_{\mathrm{out}}\right)=\max\left(\alpha_{\mathrm{in}},\alpha_{\mathrm{out}}\right)\frac{\log(n)}{n}\leq 1$, $\alpha_{\mathrm{in}}$ and $\alpha_{\mathrm{out}}$ should be set in $\left(0,\frac{n}{\log(n)}\right]$. We let $\alpha_{\mathrm{in}}$ and $\alpha_{\mathrm{out}}$ be in the range of {2.5,5,7.5,…,50}. Panel (a) of Figure 8 displays the results. We see that DiMSC performs satisfactorily when $\alpha_{\mathrm{in}}$ and $\alpha_{\mathrm{out}}$ satisfy Equation (14), and this means that DiMSC achieves the threshold provided in Equation (14) under $DiDCMM(n,K,\Pi_{r},\Pi_{c},\alpha_{\mathrm{in}},\alpha_{\mathrm{out}})$.

Experiment 5 (b): Phase transition. Under $DiDCMM(n,K,\Pi_{r},\Pi_{c},\alpha_{\mathrm{in}},\alpha_{\mathrm{out}})$, set $K=3,n=n_{r}=n_{c}=300$. Let each row community have 60 pure nodes, each column community have 80 pure nodes, and all mixed nodes have membership (1/3,1/3,1/3). We also let $\alpha_{\mathrm{in}}$ and $\alpha_{\mathrm{out}}$ be in the range of {2.5,5,7.5,…,50}. Panel (b) of Figure 8 displays the results, and the analysis is similar to that of Experiment 5 (a).

For Experiments 1–5, we can conclude that DiMSC outperforms its competitors, and this supports our analysis in Remark 6 because DiMSC is designed to estimate mixed memberships, while its competitors are designed for community partition of pure nodes.

**Figure 9:**
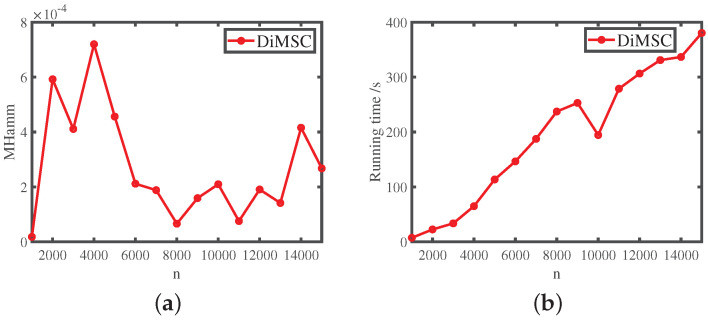
Numerical results for Experiment 6. Panel (**a**): MHamm; Panel (**b**): running time.

Experiment 6: Network size. Under $SBM(n,p_{\mathrm{in}},p_{\mathrm{out}})$, let $\alpha_{\mathrm{in}}=2$ and $\alpha_{\mathrm{out}}=0.0001$. On the one hand, we have $\sqrt{\alpha_{\mathrm{in}}}-\sqrt{\alpha_{\mathrm{out}}}=\sqrt{2}-0.01<\sqrt{2}$, i.e., $\alpha_{\mathrm{in}}$ and $\alpha_{\mathrm{out}}$ locates in the impossible region of exact recovery introduced in [50]. On the other hand, we have $\frac{\alpha_{\mathrm{in}}-\alpha_{\mathrm{out}}}{\sqrt{\alpha_{\mathrm{in}}}}>1$, i.e., $\alpha_{\mathrm{in}}$ and $\alpha_{\mathrm{out}}$ satisfy Equation (14) for DiMSC’s consistent estimation. Let *n* range in {1000,2000,3000,…,15000}. For each *n* in this experiment, we report the averaged error rate and running time of DiMSC over 10 independent repetitions. The results are shown in Figure 9. From Panel (a) of Figure 9, we see that DiMSC enjoys satisfactory performance with a small error rate for this experiment. Panels (b) of Figure 9 says that DiMSC processes computer-generated networks of up to 15,000 nodes within hundreds of seconds.

**Remark 7.**  *For visuality, we provide some examples of different types of directed networks generated under DiDCMM in this remark. Let $\theta_{r}\left(i\right)=0.9+\frac{i^{2}}{9n_{r}^{2}}$ for $1\leq i\leq n_{r}$. Let each row community has $n_{r,0}$ pure nodes, and each column community has $n_{c,0}$ pure nodes. Let all mixed nodes have membership (1/K,…,1/K). For the setting of P, we set it as*

$$\begin{array}{cc} P_{a} & =\left[\begin{array}{cc} 0.9 & 0.05\\ 0.1 & 0.95 \end{array}\right]\;\mathrm{or}\;P_{b}=\left[\begin{array}{cc} 0.1 & 0.95\\ 0.9 & 0.05 \end{array}\right]\mathrm{or}\;P_{c}=\left[\begin{array}{cc} 12 & 1\\ 0 & 12 \end{array}\right]\frac{\log(n_{r})}{n_{r}}\;\mathrm{or}\;P_{d}=\left[\begin{array}{cc} 0 & 12\\ 12 & 1 \end{array}\right]\frac{\log(n_{r})}{n_{r}}\;\mathrm{or}\;\\ P_{e} & =\left[\begin{array}{ccc} 12 & 1 & 0\\ 0 & 12 & 0\\ 1 & 0 & 12 \end{array}\right]\frac{\log(n_{r})}{n_{r}}\;\mathrm{or}\;P_{f}=\left[\begin{array}{ccc} 1 & 0 & 12\\ 12 & 0 & 0\\ 0 & 12 & 1 \end{array}\right]\frac{\log(n_{r})}{n_{r}}, \end{array}$$

*where K=2 when P is $P_{a},P_{b},P_{c}$ or $P_{d}$, and K=3 when P is $P_{e}$ or $P_{f}$. Meanwhile, we can generate different types of directed networks under DiDCMM by considering the above six different settings of P, where these different types are also considered in Experiments 1–6, and we mainly provide the visuality for these directed networks with different structures provided in different P for this remark. Note that we allow P to have non-unit diagonals here because Condition (I1) is mainly for our theoretical buildings, and results for previous experiments show that DiMSC performs stable even when P has non-unit diagonals. We consider below eight settings.*

**Figure 10:**
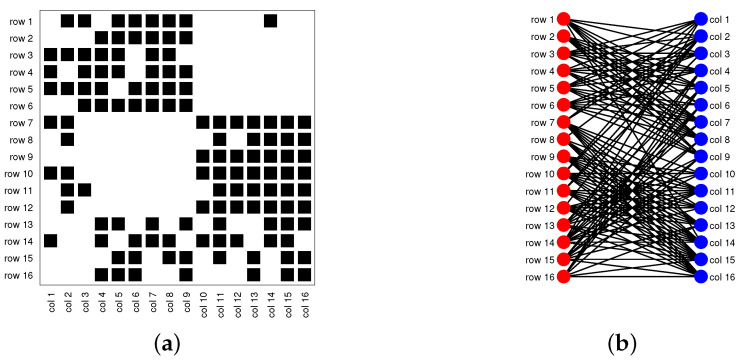
Illustration for a directed network under Model Setup 1. Panel (**a**): Adjacency matrix of N, where black square denotes 1; Panel (**b**): directed network N, where red (blue) points indicate row (column) nodes. The error rate MHamm defined in Equation (16) of our DiSMC algorithm for this directed network N is 0.0377.

Model Setup 1*: Set $n_{r}=16,n_{r,0}=6,n_{c}=16,n_{c,0}=7$, and P as $P_{a}$. For this setup, a directed network with 16 row nodes and 16 column nodes is generated from DiDCMM. Figure 10 shows a directed network N generated under Model Setup 1, where we also report DiMSC’s error rate. Figure 10 says that there are more directed edges sent from row nodes 1–6 to column nodes 1–7 than from row nodes 7–12 to column nodes 1–7 for $P_{a}$. With given adjacency matrix A and known memberships $\Pi_{r}$ and $\Pi_{c}$ for this setup, readers can apply our DiMSC directly to A given in Panel (a) of Figure 10 to check the effectiveness of DiMSC.*

**Figure 11:**
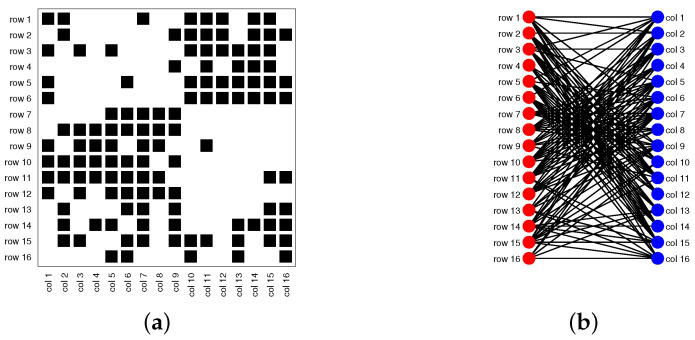
Illustration for a directed network under Model Setup 2. Panel (**a**): Adjacency matrix *A*; Panel (**b**): directed network N. MHamm of DiMSC for this directed network N is 0.0424.

Model Setup 2*: All settings are the same as Model Setup 1 except that we let P be $P_{b}$. The directed network N and its adjacency matrix are shown in Figure 11. We see that there are more directed edges sent from row nodes 1–6 to column nodes 10–16 than from row nodes 7–12 to column nodes 10–16 for $P_{b}$, which means that directed network generated using $P_{b}$ and directed network from $P_{a}$ has different structures.*

**Figure 12:**
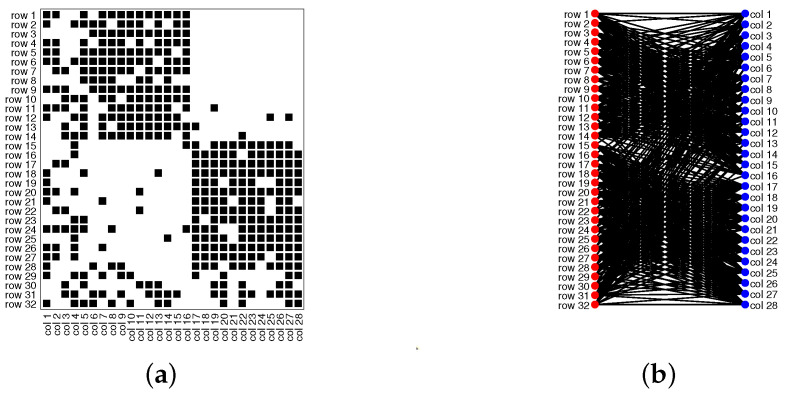
Illustration for a bipartite network under Model Setup 3. Panel (**a**): Adjacency matrix *A*; Panel (**b**): bipartite network N. MHamm of DiMSC for this bipartite network N is 0.0313.

Model Setup 3*: Set $n_{r}=32,n_{r,0}=14,n_{c}=28,n_{c,0}=12$, and P as $P_{a}$. For this setup, a bipartite network with 32 row nodes and 28 column nodes are generated from DiDCMM. Figure 12 shows this bipartite network and its adjacency matrix.*

**Figure 13:**
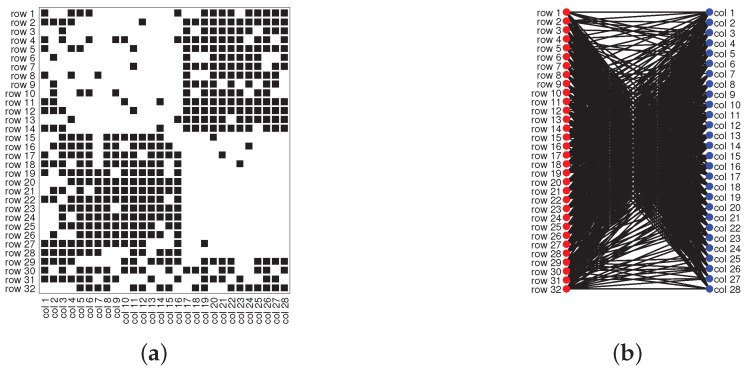
Illustration for a bipartite network under Model Setup 4. Panel (**a**): Adjacency matrix *A*; Panel (**b**): bipartite network N. MHamm of DiMSC for this bipartite network N is 0.0320.

Model Setup 4*: All settings are the same as Model Setup 3 except that we let P be $P_{b}$. Figure 13 displays the results, and we see that the bipartite network from $P_{b}$ also has a different structure compared with the one generated from using $P_{a}$ under DiDCMM.*

**Figure 14:**
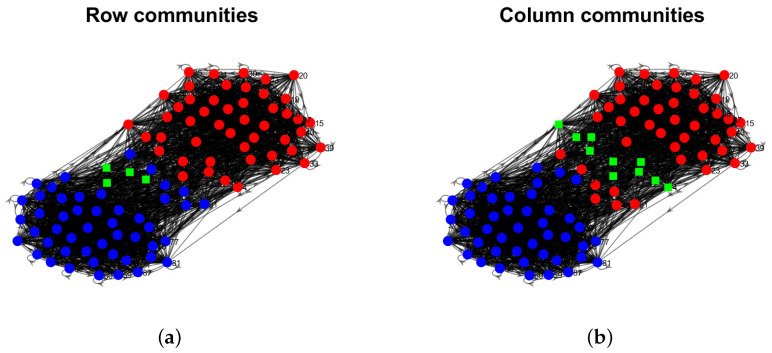
Illustration for a directed network under Model Setup 5. Panels (**a**,**b**) show the row and column communities, respectively. In these two panels, dots in the same color are pure nodes in the same communities, and a square indicates mixed nodes. MHamm of DiMSC for this directed network N is 0.0181.

Model Setup 5*: Set $n_{r}=100,n_{r,0}=48,n_{c}=100,n_{c,0}=45$, and P as $P_{c}$. Figure 14 shows the row and column communities for a directed network generated from Setup 5 under DiDCMM, where we plot the directed network directly.*

**Figure 15:**
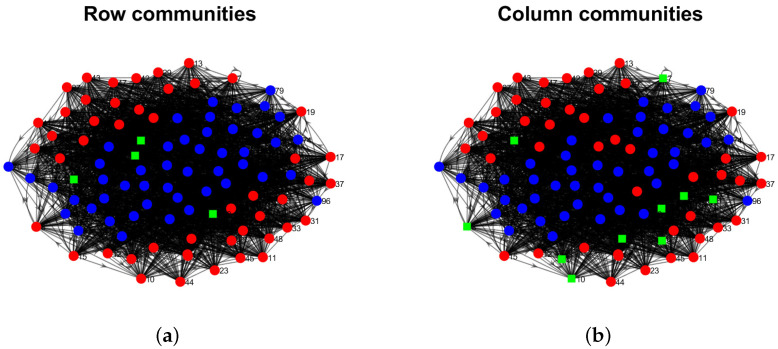
Illustration for a directed network under Model Setup 6. Panels (**a**,**b**) show the row and column communities, respectively. MHamm of DiMSC for this directed network N is 0.0185.

Model Setup 6*: All settings are the same as Model Setup 5 except that we let P be $P_{d}$. Figure 15 shows a directed network obtained from this setup, and we see that the structure of the directed network from $P_{d}$ in Figure 15 differs a lot from that of the directed network from $P_{c}$ shown in Figure 14.*

**Figure 16:**
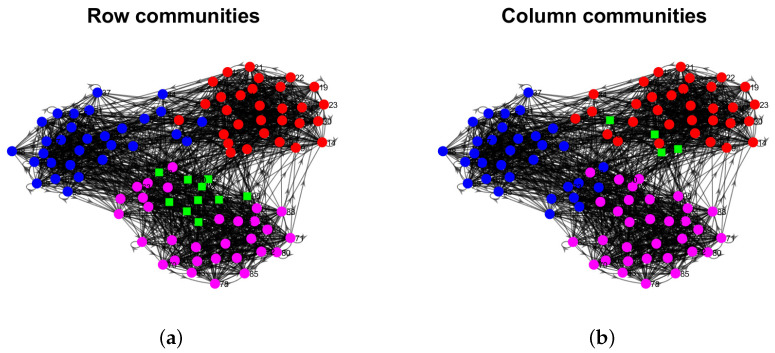
Illustration for a directed network under Model Setup 7. Panels (**a**,**b**) show the row and column communities, respectively. MHamm of DiMSC for this directed network N is 0.0266.

Model Setup 7*: Set $n_{r}=100,n_{r,0}=30,n_{c}=100,n_{c,0}=32$, and P as $P_{e}$. Figure 16 shows a directed network generated from this setup.*

**Figure 17:**
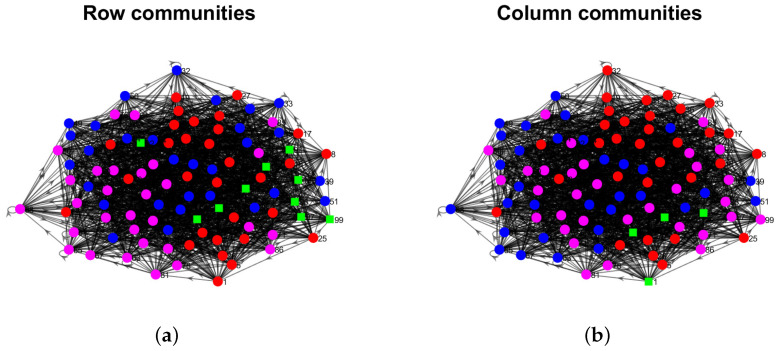
Illustration for a directed network under Model Setup 8. Panels (**a**,**b**) show the row and column communities, respectively. MHamm of DiMSC for this directed network N is 0.0279.

Model Setup 8*: All settings are the same as Model Setup 7 except that we let P be $P_{f}$. Figure 17 displays a directed network generated from this setup, and we see that directed networks from $P_{f}$ and $P_{e}$ have different structures by comparing Figure 16 and Figure 17.*

## 6. Application to Real-World Directed Networks

For the empirical directed networks considered here, row nodes are always the same as column nodes, so we let $n_{r}=n_{c}=n$. For ${\hat{\Pi }}_{r}$, we call node *i* highly mixed node if $0.8\geq \max_{1\leq k\leq K}{\hat{\Pi }}_{r}\left(i,k\right)$, similar for ${\hat{\Pi }}_{c}$. A highly mixed node tells us whether a node has mixed memberships and belongs to multiple communities. Let $\tau_{r}=\frac{|i:0.8\geq \max_{1\leq k\leq K}{\hat{\Pi }}_{r}\left(i,k\right)|}{n}$ be the proportion of highly mixed nodes among all nodes to measure the mixability of all row communities. Define $\tau_{c}$ similar to $\tau_{r}$. Let ${\hat{\ell }}_{r}$ be a vector such that ${\hat{\ell }}_{r}\left(i\right)={\mathrm{argmax}}_{1\leq k\leq K}{\hat{\Pi }}_{r}\left(i,k\right)$ for 1≤i≤n, where we use ${\hat{\ell }}_{r}\left(i\right)$ to denote the home base row community of node *i*. Define ${\hat{\ell }}_{c}$ similar to ${\hat{\ell }}_{r}$. To measure the asymmetric structure of a directed network, we use

$$\begin{array}{c} {\mathrm{Hamm}}_{rc}=\frac{\min_{P\in S_{P}}{∥{\hat{\Pi }}_{c}P-{\hat{\Pi }}_{r}∥}_{1}}{n}, \end{array}$$

where a large ${\mathrm{Hamm}}_{rc}$ means that the structure of row clusters differs a lot from that of column clusters. For 1≤i≤n, let $d_{r}\left(i\right)=\sum_{j=1}^{n}A\left(i,j\right)$ be the number of edges sent by node *i*, $d_{c}\left(i\right)=\sum_{j=1}^{n}A\left(j,i\right)$ be the number of edges received by node *i*, where $d_{r}\left(i\right)$ (and $d_{c}\left(i\right)$) is the out degree (in degree) of node *i*. Since there are many nodes with zero in degree or out degree for real-world directed network, we need the below pre-processing: for any directed network N, we let $A_{m}$ be its adjacency matrix for any positive integer *m* such that $A_{m}$ is connected, and every node has at least *m* in degree and *m* out degree in $A_{m}$.

We apply DiMSC to the following real-world directed networks to discover their mixability, asymmetries, and directional communities.

**Political blogs:** This is a directed network of hyperlinks between weblogs on US politics [68]. In this data, node means a blog, and edge means a hyperlink. This data can be downloaded from http://www-personal.umich.edu/~mejn/netdata/ (accessed on 28 August 2022). It is well-known that there are two parties, “liberal” and “conservative”, so K=2 for this data. The are 1490 nodes in the original data. After pre-processing, $A_{1}\in {\left\{0,1\right\}}^{813\times 813},A_{3}\in {\left\{0,1\right\}}^{495\times 495},A_{6}\in {\left\{0,1\right\}}^{285\times 285},A_{9}\in {\left\{0,1\right\}}^{158\times 158}$, where we focus on the cases when m=1,3,6,9 for this data here. Meanwhile, we use political blogs $A_{m}$ to denote this network when its adjacency matrix is $A_{m}$, where every node has a degree at least *m*. Similar notations hold for other real-world directed networks used in this paper.

**Figure 18:**
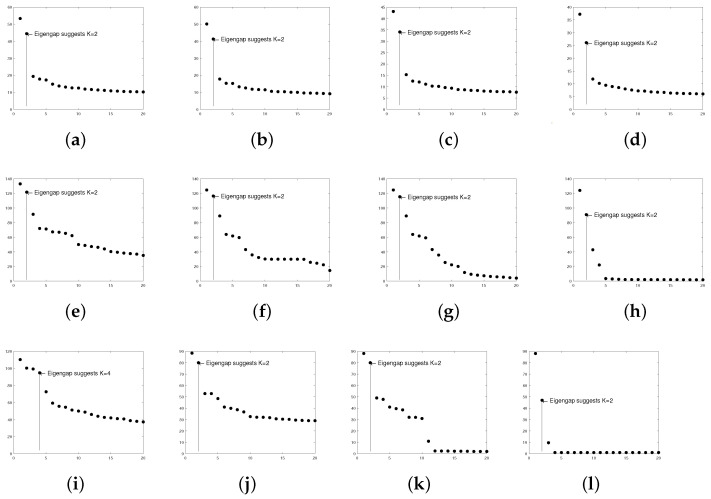
Leading 20 singular values of real-world directed networks used in this paper. Panel (**a**): political blogs $A_{1}$; Panel (**b**): political blogs $A_{3}$; Panel (**c**): political blogs $A_{6}$; Panel (**d**): political blogs $A_{9}$; Panel (**e**): Wikipedia links (gan) $A_{1}$; Panel (**f**): Wikipedia links (gan) $A_{30}$; Panel (**g**): Wikipedia links (gan) $A_{60}$; Panel (**h**): Wikipedia links (gan) $A_{90}$; Panel (**i**): Wikipedia links (nah) $A_{1}$; Panel (**j**): Wikipedia links (nah) $A_{20}$; Panel (**k**): Wikipedia links (nah) $A_{30}$; Panel (**l**): Wikipedia links (nah) $A_{40}$.

**Wikipedia links (gan)**: This directed network consists of the Wikilinks of Wikipedia in the Gan Chinese language (gan). In this data, node means an article, and the directed edge is a Wikilink [69]. This data can be downloaded from http://konect.cc/networks/wikipedia_link_gan (accessed on 28 August 2022). There are 9189 nodes in the original data. After pre-processing, $A_{1}\in {\left\{0,1\right\}}^{6012\times 6012},A_{30}\in {\left\{0,1\right\}}^{820\times 820},A_{60}\in {\left\{0,1\right\}}^{559\times 569},A_{90}\in {\left\{0,1\right\}}^{240\times 240}$, where we study the cases m=1,30,60,90 for this data. The leading 20 singular values of $A_{1},A_{30},A_{60},A_{90}$ shown in Panels (e)–(h) of Figure 18 suggest K=2 for these four adjacency matrices, where [30] also uses eigengap to estimate *K*.

**Wikipedia links (nah)**: This network consists of the Wikilinks of the N $\bar{a}$huatl language (nah) [69] and can be downloaded from http://konect.cc/networks/wikipedia_link_nah/ (accessed on 28 August 2022). The original data has 10285 nodes. After pre-processing, $A_{1}\in {\left\{0,1\right\}}^{6924\times 6924},A_{20}\in {\left\{0,1\right\}}^{1057\times 1057},A_{30}\in {\left\{0,1\right\}}^{486\times 486},A_{40}\in {\left\{0,1\right\}}^{136\times 136}$. Panel (i) of Figure 18 suggests K=4 for $A_{1}$, and Panels (j)–(l) of Figure 18 suggest K=2 for $A_{20},A_{30}$, and $A_{40}$. Note that it only takes around 4 seconds for DiMSC to estimate memberships of Wikipedia links (nah) $A_{1}$.

**Figure 19:**
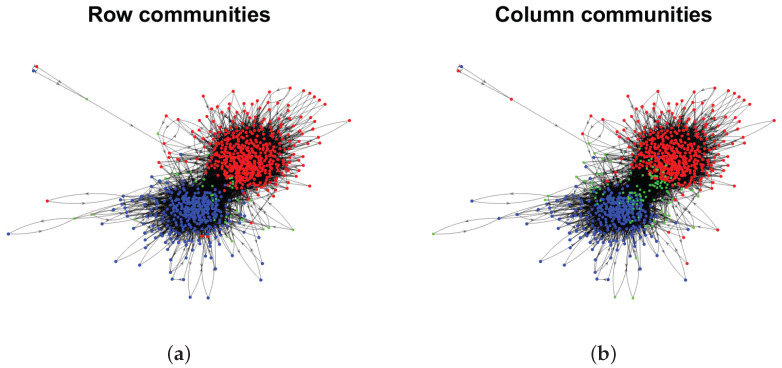

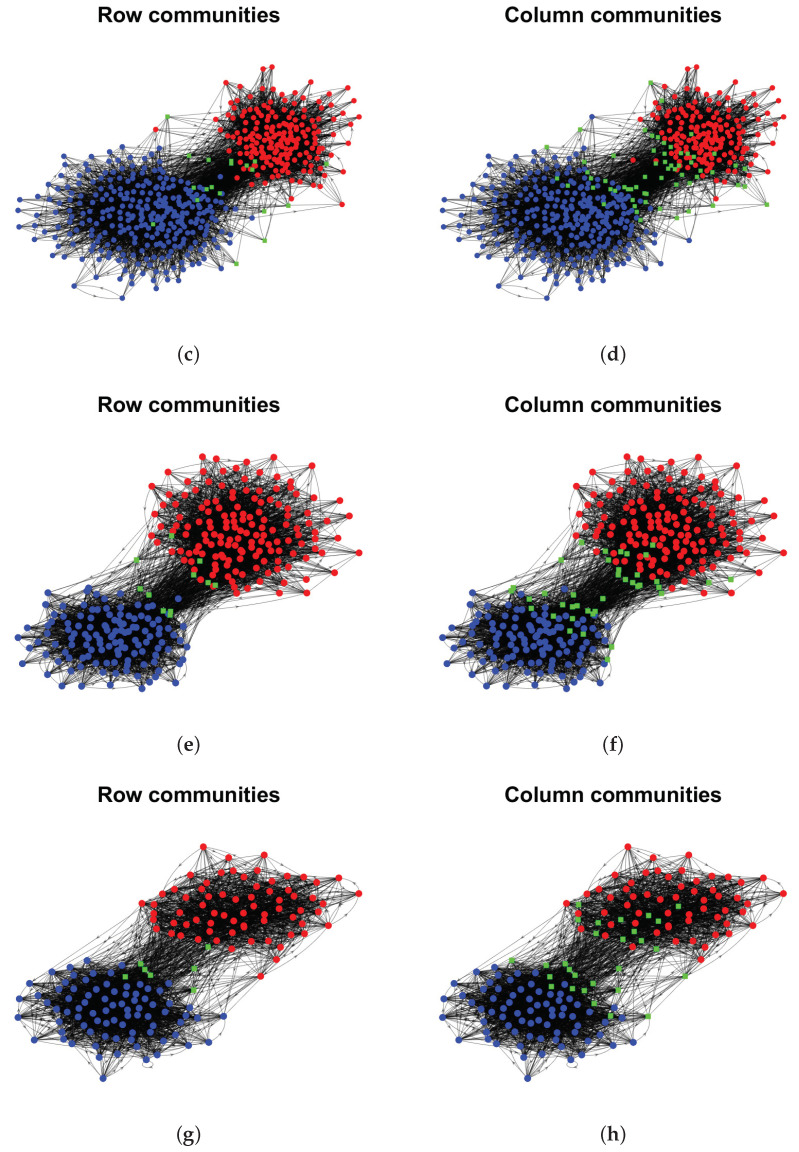
Row and column communities detected by DiMSC for political blogs. Colors indicate clusters, and a green square indicates highly mixed nodes, where the row and column communities are obtained from ${\hat{\ell }}_{r}$ and ${\hat{\ell }}_{c}$, respectively. Panel (**a**): political blogs $A_{1}$; Panel (**b**): political blogs $A_{1}$; Panel (**c**): political blogs $A_{3}$; Panel (**d**): political blogs $A_{3}$; Panel (**e**): political blogs $A_{6}$; Panel (**f**): political blogs $A_{6}$; Panel (**g**): political blogs $A_{9}$; Panel (**h**): political blogs $A_{9}$.

**Figure 20:**
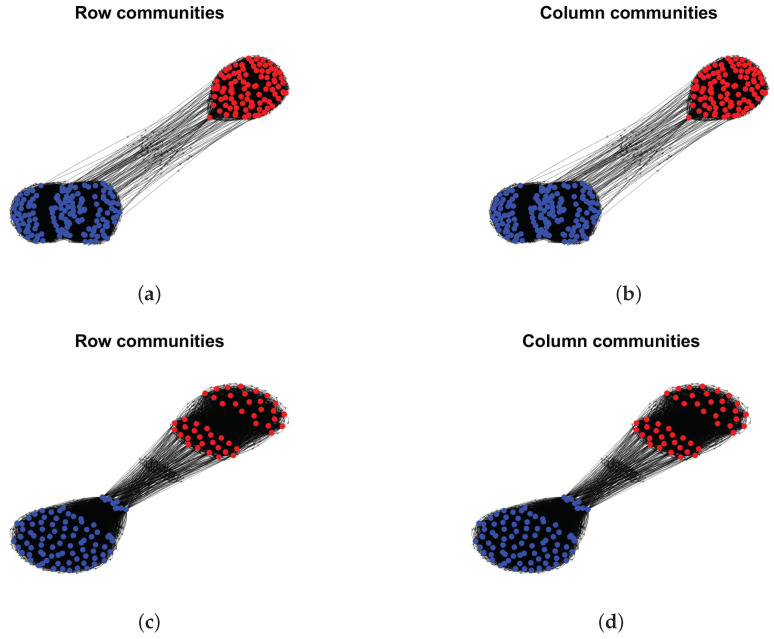
Row and column communities detected by DiMSC for Wikipedia links (gan) $A_{90}$ and Wikipedia links (nah) $A_{40}$. Colors indicate clusters, where the row and column communities are obtained from ${\hat{\ell }}_{r}$ and ${\hat{\ell }}_{c}$, respectively. Panel (**a**): Wikipedia links (gan) $A_{90}$; Panel (**b**): Wikipedia links (gan) $A_{90}$; Panel (**c**): Wikipedia links (nah) $A_{40}$; Panel (**d**): Wikipedia links (nah) $A_{40}$.

The proportions of highly mixed nodes and ${\mathrm{Hamm}}_{rc}$ when applying DiMSC on the above real-world directed networks are reported in Table 1. For the political blogs network, small $\tau_{r},\tau_{c}$, and ${\mathrm{Hamm}}_{rc}$ indicate that there are only a few highly mixed nodes, and the structure of row communities is similar to that of column communities, i.e., there is a slight asymmetry for this data. For Wikipedia links (gan) $A_{1}$ and Wikipedia links (nah) $A_{1}$, they have a large proposition of highly mixed nodes in both row and column communities, and the row communities differ a lot from column communities, suggesting heavy asymmetric structure between row and column communities for these two data. For Wikipedia links (gan) $A_{30},A_{60}$, and Wikipedia links (nah) $A_{20}$, we see that the proportion of highly mixed nodes for row (column) communities is small (large), and there is a slight asymmetric for these data. For Wikipedia links (gan) $A_{90}$ and Wikipedia links (nah) $A_{30},A_{40}$, there is no highly mixed node, and the structure of row clusters is similar to that of column clusters. For visualization, we plot the row and column communities as well as highly mixed nodes by applying DiMSC to some of these directed networks in Figure 19 and Figure 20.

## 7. Discussion and Conclusions

In this paper, we propose a novel directed degree corrected mixed membership (DiDCMM) model. DiDCMM models a directed network with mixed memberships for row nodes with degree heterogeneities and column nodes without degree heterogeneities. DiDCMM is identifiable when the two well-used Conditions (I1) and (I2) hold. It should be mentioned that a model modeling a directed network with mixed memberships for both row and column nodes with degree heterogeneities is unidentifiable unless considering some nontrivial conditions. To fit the model, we propose a provably consistent spectral algorithm called DiMSC to infer community memberships for both row and column nodes in a directed network generated by DiDCMM. DiMSC is designed based on the SVD of the adjacency matrix, where we apply the SP algorithm to hunt for the corners in the simplex structure and the SVM-cone algorithm to hunt for the corners in the cone structure. The theoretical results of DiMSC show that it consistently recovers memberships of both row nodes and column nodes under mild conditions. Meanwhile, when DiDCMM degenerates to MMSB, our theoretical results match that of Theorem 2.2 [24] when their DCMM degenerates to MMSB under mild conditions. Experiments conducted on synthetic directed networks generated from DiDCMM verify the effectiveness and the stability of Conditions (I1) and (I2) of DiMSC. Results for real-world directed networks show that DiMSC reveals highly mixed nodes and asymmetries in the structure of row and column communities. The model DiDCMM and the algorithm DiMSC developed in this paper are useful to discover asymmetry for a directed network with mixed memberships. DiDCMM can also generate an artificially directed network with mixed memberships as a benchmark directed network for research purposes. We wish that DiDCMM and DiMSC can be widely applied in social network analysis.

The proposed model DiDCMM and the algorithm DiMSC can be extended in many ways. Similar to [24,57], we may obtain an ideal simplex from *U* using the idea of the entry-wise ratio proposed in [8]. Meanwhile, DiMSC is designed based on the SVD of the adjacency matrix, and similar to [5,7,11,30], we may design spectral algorithms based on the regularized Laplacian matrix under DiDCMM. Extending DiDCMM from an un-weighted directed network to a weighted directed network with an application of the distribution-free idea introduced in [62] is one of our future research directions. The SVD step of DiMSC can be accelerated by the random projection and random sampling ideas introduced in [70] to process large-scale directed networks. Instead of simply using eigengap to find *K*, in our future work, it is worth focusing on estimating the number of communities in a directed network generated under ScBM (and DCScBM) [30] and DiDCMM. Ref. [46] proposes an algorithm to uncover boundary nodes that spread information between communities in undirected social networks. It is an interesting topic to extend works in [46] to directed networks generated from ScBM, DCScBM, and DiDCMM. We leave them for our future work.

## Figures and Tables

**Table 1:** $\tau_{r},\tau_{c}$, and ${\mathrm{Hamm}}_{rc}$ obtained from DiMSC for real-world directed networks used in this paper.

Data	τ ${}_{r}$	τ ${}_{c}$	Hamm${}_{\mathrm{rc}}$
Political blogs $A_{1}$	0.0455	0.1353	0.0893
Political blogs $A_{3}$	0.0481	0.1570	0.0705
Political blogs $A_{6}$	0.0386	0.1368	0.0662
Political blogs $A_{9}$	0.0443	0.1772	0.0771
Wikipedia links (gan) $A_{1}$	0.1505	0.6051	0.3528
Wikipedia links (gan) $A_{30}$	0.0817	0.1902	0.0547
Wikipedia links (gan) $A_{60}$	0.0054	0.1145	0.0664
Wikipedia links (gan) $A_{90}$	0	0	0.0203
Wikipedia links (nah) $A_{1}$	0.2718	0.3521	0.2065
Wikipedia links (nah) $A_{20}$	0.0937	0.1722	0.0488
Wikipedia links (nah) $A_{30}$	0	0	0.0046
Wikipedia links (nah) $A_{40}$	0	0	0

## Data Availability

Not applicable.

## Abbreviations

    The following abbreviations are used in this manuscript:

SBM	Stochastic Blockmodel
DCSBM	Degree Corrected Stochastic Blockmodel
MMSB	Mixed Membership Stochastic Blockmodel
DCMM	Degree Corrected Mixed Membership model
OCCAM	Overlapping Continuous Community Assignment model
ScBM	Stochastic co-Blockmodel
DC-ScBM	Degree Corrected Stochastic co-Blockmodel
DiMMSB	Directed Mixed Membership Stochastic Blockmodel
DiDCMM	Directed Degree Corrected Mixed Membership model
SP	Successive projection algorithm
SVD	Singular value decomposition
DiMSC	Directed Mixed Simplex & Cone algorithm

## Appendix A. Proof for Identifiability

### Appendix A.1. Proof of Proposition 1

**Proof.** Let $\Omega =U\Lambda V^{\prime }$ be the compact singular value decomposition of Ω. Lemma 1 gives $V=\Pi_{c}B_{c}\equiv \Pi_{c}V\left(I_{c},:\right)$. Since $\Omega =\tilde{\Omega }$, *V* also equals to ${\tilde{\Pi }}_{c}V\left(I_{c},:\right)$, which gives that $\Pi_{c}={\tilde{\Pi }}_{c}$. Since $\Omega \left(I_{r},I_{c}\right)=\Theta_{r}\left(I_{r},I_{r}\right)\Pi_{r}\left(I_{r},:\right)P\Pi_{c}^{\prime }\left(I_{c},:\right)=\Theta_{r}\left(I_{r},I_{r}\right)P=U\left(I_{r},:\right)\Lambda V^{\prime }\left(I_{c},:\right)$ by Condition (I2), we have $\Theta_{r}\left(I_{r},I_{r}\right)P=U\left(I_{r},:\right)\Lambda V^{\prime }\left(I_{c},:\right)$, which gives that $\Theta_{r}\left(I_{r},I_{r}\right)=\mathrm{diag}\left(U\left(I_{r},:\right)\Lambda V^{\prime }\left(I_{c},:\right)\right)$. From this step, we see that if *P*’s diagonal entries are not ones, we cannot obtain $\Theta_{r}\left(I_{r},I_{r}\right)=\mathrm{diag}\left(U\left(I_{r},:\right)\Lambda V^{\prime }\left(I_{c},:\right)\right)$ which leads to a consequence that $\Theta_{r}\left(I_{r},I_{r}\right)$ does not equal to ${\tilde{\Theta }}_{r}\left(I_{r},I_{r}\right)$; hence Condition (I1) is necessary by Condition (I1). Since $\Omega =\tilde{\Omega }$, we also have ${\tilde{\Theta }}_{r}\left(I_{r},I_{r}\right)=\mathrm{diag}\left(U\left(I_{r},:\right)\Lambda V^{\prime }\left(I_{c},:\right)\right)$, which gives that $\Theta_{r}\left(I_{r},I_{r}\right)={\tilde{\Theta }}_{r}\left(I_{r},I_{r}\right)$. Since ${\tilde{\Theta }}_{r}\left(I_{r},I_{r}\right)\tilde{P}$ also equals to $U\left(I_{r},:\right)\Lambda V^{\prime }\left(I_{c},:\right)$, we have $P=\tilde{P}$. Lemma 1 gives that $U=\Theta_{r}\Pi_{r}B_{r}$, where $B_{r}=\Theta_{r}^{-1}\left(I_{r},I_{r}\right)U\left(I_{r},:\right)$. Since $\Omega =\tilde{\Omega }$, we also have $U={\tilde{\Theta }}_{r}{\tilde{\Pi }}_{r}{\tilde{B}}_{r}$. Since ${\tilde{B}}_{r}={\tilde{\Theta }}_{r}^{-1}\left(I_{r},I_{r}\right)U\left(I_{r},:\right)=\Theta_{r}^{-1}\left(I_{r},I_{r}\right)U\left(I_{r},:\right)$, we have ${\tilde{B}}_{r}=B_{r}$. Since $U=\Theta_{r}\Pi_{r}B_{r}={\tilde{\Theta }}_{r}{\tilde{\Pi }}_{r}{\tilde{B}}_{r}={\tilde{\Theta }}_{r}{\tilde{\Pi }}_{r}B_{r}$, we have $\Theta_{r}\Pi_{r}={\tilde{\Theta }}_{r}{\tilde{\Pi }}_{r}$. Since each row of $\Pi_{r}$ or ${\tilde{\Pi }}_{r}$ is a PMF, $\Theta_{r}={\tilde{\Theta }}_{r},\Pi_{r}={\tilde{\Pi }}_{r}$, and the claim follows.    □

## Appendix B. Ideal Simplex, Ideal Cone

### Appendix B.1. Proof of Lemma 1

**Proof.** First, we consider *U* and *V*. Since $\Omega =U\Lambda V^{\prime }$, we have $U=\Omega V\Lambda^{-1}$ since $V^{\prime }V=I_{K}$. Recall that $\Omega =\Theta_{r}\Pi_{r}P\Pi_{c}^{\prime }$, we have $U=\Theta_{r}\Pi_{r}P\Pi_{c}^{\prime }V\Lambda^{-1}=\Theta_{r}\Pi_{r}B_{r}$, where we set $B_{r}=P\Pi_{c}^{\prime }V\Lambda^{-1}$ and sure it is unique. Since $U\left(I_{r},:\right)=\Theta_{r}\left(I_{r},I_{r}\right)\Pi_{r}\left(I_{r},:\right)B_{r}=\Theta_{r}\left(I_{r},I_{r}\right)B_{r}$, we have $B_{r}=\Theta_{r}^{-1}\left(I_{r},I_{r}\right)U\left(I_{r},:\right)$. Similarly, since $\Omega =U\Lambda V^{\prime }$, we have $V^{\prime }=\Lambda^{-1}U^{\prime }\Omega$ since $U^{\prime }U=I_{K}$, hence $V=\Omega^{\prime }U\Lambda^{-1}$. Recall that $\Omega =\Theta_{r}\Pi_{r}P\Pi_{c}^{\prime }$, we have $V={\left(\Theta_{r}\Pi_{r}P\Pi_{c}^{\prime }\right)}^{\prime }U\Lambda^{-1}=\Pi_{c}P^{\prime }\Pi_{r}^{\prime }\Theta_{r}U\Lambda^{-1}=\Pi_{c}B_{c}$, where we set $B_{c}=P^{\prime }\Pi_{r}^{\prime }\Theta_{r}U\Lambda^{-1}$ and sure it is unique. Since $V\left(I_{c},:\right)=\Pi_{c}\left(I_{c},:\right)B_{c}=B_{c}$, we have $B_{c}=V\left(I_{c},:\right)$. Meanwhile, for $1\leq j\leq n_{c}$, we have $V\left(j,:\right)=e_{j}^{\prime }\Pi_{c}B_{c}=\Pi_{c}\left(j,:\right)B_{c}$. Hence, we have $V\left(j,:\right)=V\left(\bar{j},:\right)$ as long as $\Pi_{c}\left(j,:\right)=\Pi_{c}\left(\bar{j},:\right)$. Now, we show the ideal cone structure that appears in $U_{*}$. For convenience, set $M=\Pi_{r}B_{r}$, hence $U=\Theta_{r}\Pi_{r}B_{r}$ gives $U=\Theta_{r}M$. Hence, we have $U\left(i,:\right)=e_{i}^{\prime }U=\Theta_{r}\left(i,i\right)M\left(i,:\right)$. Therefore, $U_{*}\left(i,:\right)=\frac{U(i,:)}{{∥U\left(i,:\right)∥}_{F}}=\frac{M(i,:)}{{∥M\left(i,:\right)∥}_{F}}$, combine it with the fact that $B_{r}=\Theta_{r}^{-1}\left(I_{r},I_{r}\right)U\left(I_{r},:\right)$, we have

$$\begin{array}{cc} U_{*} & =\left[\begin{array}{cccc} \frac{1}{{∥M\left(1,:\right)∥}_{F}} & & & \\ & \frac{1}{{∥M\left(2,:\right)∥}_{F}} & & \\ & & \ddots & \\ & & & \frac{1}{∥M\left(n_{r},:\right){∥}_{F}} \end{array}\right]\Pi_{r}B_{r}\\ & =\left[\begin{array}{c} \Pi_{r}\left(1,:\right)/{∥M\left(1,:\right)∥}_{F}\\ \Pi_{r}\left(2,:\right)/{∥M\left(2,:\right)∥}_{F}\\ \vdots \\ \Pi_{r}\left(n_{r},:\right)/{∥M\left(n_{r},:\right)∥}_{F} \end{array}\right]\Theta_{r}^{-1}\left(I_{r},I_{r}\right)N_{U}^{-1}\left(I_{r},I_{r}\right)U_{*}\left(I_{r},:\right). \end{array}$$

Therefore, we have

$$\begin{array}{c} Y=\left[\begin{array}{c} \Pi_{r}\left(1,:\right)/{∥M\left(1,:\right)∥}_{F}\\ \Pi_{r}\left(2,:\right)/{∥M\left(2,:\right)∥}_{F}\\ \vdots \\ \Pi_{r}\left(n_{r},:\right)/{∥M\left(n_{r},:\right)∥}_{F} \end{array}\right]\Theta_{r}^{-1}\left(I_{r},I_{r}\right)N_{U}^{-1}\left(I_{r},I_{r}\right)=N_{M}\Pi_{r}\Theta_{r}^{-1}\left(I_{r},I_{r}\right)N_{U}^{-1}\left(I_{r},I_{r}\right), \end{array}$$

where $N_{M}$ is a diagonal matrix with $N_{M}\left(i,i\right)=\frac{1}{{∥M\left(i,:\right)∥}_{F}}$ for $1\leq i\leq n_{r}$. All entries of *Y* are nonnegative, and since we assume that each community has at least one pure node, no row of *Y* is 0. Then, we prove that $U_{*}\left(i,:\right)=U_{*}\left(\bar{i},:\right)$ when $\Pi_{r}\left(i,:\right)=\Pi_{r}\left(\bar{i},:\right)$. For $1\leq i\leq n_{r}$, we have

$$\begin{array}{cc} U_{*}\left(i,:\right) & =e_{i}^{\prime }U_{*}=\frac{1}{{∥M\left(i,:\right)∥}_{F}}e_{i}^{\prime }M=\frac{1}{∥\Pi_{r}\left(i,:\right)B_{r}{∥}_{F}}\Pi_{r}\left(i,:\right)B_{r}, \end{array}$$

and the claim follows immediately.    □

### Appendix B.2. Proof of Lemma 2

**Proof.** Since $I=U^{\prime }U=B_{r}^{\prime }\Pi_{r}^{\prime }\Theta_{r}^{2}\Pi_{r}B_{r}=U^{\prime }\left(I_{r},:\right)\Theta_{r}^{-1}\left(I_{r},I_{r}\right)\Pi_{r}^{\prime }\Theta_{r}^{2}\Pi_{r}\Theta^{-1}\left(I_{r},I_{r}\right)U\left(I_{r},:\right)$ and $\mathrm{rank}(U\left(I_{r},:\right))=K$ (i.e., the inverse of $U(I_{r},:)$ exists), we have ${\left(U\left(I_{r},:\right)U^{\prime }\left(I_{r},:\right)\right)}^{-1}=\Theta_{r}^{-1}\left(I_{r},I_{r}\right)\Pi_{r}^{\prime }\Theta_{r}^{2}\Pi_{r}\Theta_{r}^{-1}\left(I_{r},I_{r}\right)$. Since $U_{*}\left(I_{r},:\right)=N_{U}\left(I_{r},I_{r}\right)U\left(I_{r},:\right)$, we have

$$\begin{array}{c} {\left(U_{*}\left(I_{r},:\right)U_{*}^{\prime }\left(I_{r},:\right)\right)}^{-1}=N_{U}^{-1}\left(I_{r},I_{r}\right)\Theta^{-1}\left(I_{r},I_{r}\right)\Pi_{r}^{\prime }\Theta_{r}^{2}\Pi_{r}\Theta_{r}^{-1}\left(I_{r},I_{r}\right)N_{U}^{-1}\left(I_{r},I_{r}\right). \end{array}$$

Since all entries of $N_{U}^{-1}\left(I_{r},I_{r}\right),\Pi_{r},\Theta_{r}$ and nonnegative and $N,\Theta_{r}$ are diagonal matrices, we see that all entries of ${\left(U_{*}\left(I_{r},:\right)U_{*}^{\prime }\left(I_{r},:\right)\right)}^{-1}$ are nonnegative, and its diagonal entries are strictly positive, hence we have ${\left(U_{*}\left(I_{r},:\right)U_{*}^{\prime }\left(I_{r},:\right)\right)}^{-1}1>0$.    □

### Appendix B.3. Proof of Theorem 1

**Proof.** For column nodes, Remark A1 guarantees that SP algorithm returns $I_{c}$ when the input is *V* with *K* column communities, hence ideal DiMSC recovers $\Pi_{c}$ exactly. For row nodes, Remark A2 guarantees that SVM-cone algorithm returns $I_{r}$ when the input is $U_{*}$ with *K* row communities, hence ideal DiMSC recovers $\Pi_{r}$ exactly, and this theorem follows.    □

## Appendix C. Equivalence Algorithm

In this subsection, we design one algorithm DiMSC-equivalence which returns the same estimations as DiMSC. Set $U_{2}=UU^{\prime }\in R^{n_{r}\times n_{r}},{\hat{U}}_{2}=\hat{U}{\hat{U}}^{\prime }\in R^{n_{r}\times n_{r}},V_{2}=VV^{\prime }\in R^{n_{c}\times n_{c}},{\hat{V}}_{2}=\hat{V}{\hat{V}}^{\prime }\in R^{n_{c}\times n_{c}}$. Set $U_{*,2}\in R^{n_{r}\times n_{r}}$ as $U_{*,2}\left(i,:\right)=\frac{U_{2}\left(i,:\right)}{∥U_{2}{\left(i,:\right)∥}_{F}}$ for $1\leq i\leq n_{r}$. ${\hat{U}}_{*,2}$ is defined similarly. The next lemma guarantees that $V_{2}$ enjoys IS structure, and $U_{*,2}$ enjoys IC structure.

**Lemma A1.** 
*Under $DiDCMM_{n_{r},n_{c}}\left(K,P,\Pi_{r},\Pi_{c},\Theta_{r}\right)$, we have $V_{2}=\Pi_{c}V_{2}\left(I_{c},:\right)$, and $U_{*,2}=YU_{*,2}\left(I_{r},:\right)$.*

**Proof.** By Lemma 1, we know that $V=\Pi_{c}V\left(I_{c},:\right)$, which gives that $V_{2}=VV^{\prime }=\Pi_{c}V\left(I_{c},:\right)V^{\prime }=\Pi_{c}\left(VV^{\prime }\right)\left(I_{c},:\right)=\Pi_{c}V_{2}\left(I_{c},:\right)$. For *U*, since $U=\Theta_{r}\Pi_{r}\Theta_{r}^{-1}\left(I_{r},I_{r}\right)U\left(I_{r},:\right)$ by Lemma 1, we have $U_{2}=UU^{\prime }=\Theta_{r}\Pi_{r}\Theta_{r}^{-1}\left(I_{r},I_{r}\right)U\left(I_{r},:\right)U^{\prime }=\Theta_{r}\Pi_{r}\Theta_{r}^{-1}\left(I_{r},I_{r}\right)\left(UU^{\prime }\right)\left(I_{r},:\right)=\Theta_{r}\Pi_{r}\Theta_{r}^{-1}\left(I_{r},I_{r}\right)U_{2}\left(I_{r},:\right)$. Set $M_{2}=\Pi_{r}\Theta_{r}^{-1}\left(I_{r},I_{r}\right)U_{2}\left(I_{r},:\right)$, we have $U_{2}=\Theta_{r}M_{2}$. Then, follow similar proof as Lemma 1, we have $U_{*,2}=Y_{2}U_{*,2}\left(I_{r},:\right)$, where $Y_{2}=N_{M_{2}}\Pi_{r}\Theta_{r}^{-1}\left(I_{r},I_{r}\right)N_{U_{2}}^{-1}\left(I_{r},I_{r}\right)$, and $N_{M_{2}},N_{U_{2}}$ are $n_{r}\times n_{r}$ diagonal matrices whose *i*-th diagonal entries are $\frac{1}{∥M_{2}{\left(i,:\right)∥}_{F}},\frac{1}{∥U_{2}{\left(i,:\right)∥}_{F}}$, respectively. Since $∥U_{2}{\left(i,:\right)∥}_{F}=∥U\left(i,:\right)U^{\prime }{∥}_{F}={∥U\left(i,:\right)∥}_{F}$, we have $N_{U_{2}}=N_{U}$. Since $∥M_{2}{\left(i,:\right)|}_{F}=∥\Pi_{r}\Theta_{r}^{-1}\left(I_{r},I_{r}\right)U_{2}\left(I_{r},:\right){∥}_{F}=∥\Pi_{r}\Theta_{r}^{-1}\left(I_{r},I_{r}\right)U\left(I_{r},:\right)U^{\prime }{∥}_{F}={∥M\left(i,:\right)∥}_{F}$, we have $N_{M_{2}}=N_{M}$. Hence, $Y_{2}\equiv Y$ and the claim follows.    □

Since $U_{*,2}\left(I_{r},:\right)\in R^{K\times n_{r}}$ and $V_{2}\left(I_{c},:\right)\in R^{K\times n_{c}}$, $U_{*,2}\left(I_{r},:\right)$ and $V_{2}\left(I_{c},:\right)$ are singular matrix with rank *K* by Condition (I1), while the inverses of $U_{*,2}\left(I_{r},:\right)U_{*,2}^{\prime }\left(I_{r},:\right)$ and $V_{2}\left(I_{c},:\right)V_{2}^{\prime }\left(I_{c},:\right)$ exist. Therefore, Lemma A1 gives that

$$\begin{array}{c} Y=U_{*,2}U_{*,2}^{\prime }\left(I_{r},:\right){\left(U_{*,2}\left(I_{r},:\right)U_{*,2}^{\prime }\left(I_{r},:\right)\right)}^{-1},\Pi_{c}=V_{2}V_{2}^{\prime }\left(I_{c},:\right){\left(V_{2}\left(I_{c},:\right)V_{2}^{\prime }\left(I_{c},:\right)\right)}^{-1}. \end{array}$$

Since $U_{*,2}=N_{U}U_{2}$ and $Y=N_{M}\Pi_{r}\Theta_{r}^{-1}\left(,I_{r},I_{r}\right)N_{U}^{-1}\left(I_{r},I_{r}\right)$, we see that $Y_{*}$ also equals to $U_{2}U_{*,2}^{\prime }\left(I_{r},:\right){\left(U_{*,2}\left(I_{r},:\right)U_{*,2}^{\prime }\left(I_{r},:\right)\right)}^{-1}$ by basic algebra.

Based on the above analysis, we are now ready to give the ideal DiMSC-equivalence. Input Ω. Output: $\Pi_{r}$ and $\Pi_{c}$.

- Obtain $U,\Lambda ,V,U_{*,2},V_{2}$ from Ω.
- Run SP algorithm on $V_{2}$ with *K* column communities to obtain $V_{2}\left(I_{c},:\right)$. Run SVM-cone algorithm on $U_{*,2}$ with *K* row communities to obtain $I_{r}$.
- Set $J_{*}=\mathrm{diag}\left(U_{*}\left(I_{r},:\right)\Lambda V^{\prime }\left(I_{c},:\right)\right),Y_{*}=U_{2}U_{*,2}^{\prime }\left(I_{r},:\right){\left(U_{*,2}\left(I_{r},:\right)U_{*,2}^{\prime }\left(I_{r},:\right)\right)}^{-1},Z_{r}=Y_{*}J_{*}$ and $Z_{c}=V_{2}V_{2}^{\prime }\left(I_{c},:\right){\left(V_{2}\left(I_{c},:\right)V_{2}^{\prime }\left(I_{c},:\right)\right)}^{-1}$.
- Recover $\Pi_{r}$ and $\Pi_{c}$ by setting $\Pi_{r}\left(i,:\right)=\frac{Z_{r}\left(i,:\right)}{∥Z_{r}{\left(i,:\right)∥}_{1}}$ for $1\leq i\leq n_{r}$, and $\Pi_{c}\left(j,:\right)=\frac{Z_{c}\left(j,:\right)}{∥Z_{c}{\left(j,:\right)∥}_{1}}$ for $1\leq j\leq n_{c}$.

For the real case, set ${\hat{U}}_{2}=\hat{U}{\hat{U}}^{\prime },{\hat{V}}_{2}=\hat{V}{\hat{V}}^{\prime },{\hat{U}}_{*,2}=N_{\hat{U}}{\hat{U}}_{2}$. We now extend the ideal case to the real one given by Algorithm A1.

**Algorithm A1:** DiMSC-equivalence
**Require:** The adjacency matrix $A\in R^{n_{r}\times n_{c}}$ of a directed network, the number of row communities (column communities) *K*. **Ensure:** The estimated $n_{r}\times K$ row membership matrix ${\hat{\Pi }}_{r,2}$ and the estimated $n_{c}\times K$ column membership matrix ${\hat{\Pi }}_{c,2}$. 1: Obtain $\tilde{A}=\hat{U}\hat{\Lambda }{\hat{V}}^{\prime }$, the top-*K*-dimensional SVD of *A*. Compute ${\hat{U}}_{*},{\hat{U}}_{2},{\hat{V}}_{2},{\hat{U}}_{*,2}$. 2: Apply SP algorithm on the rows of ${\hat{V}}_{2}$ assuming there are *K* column communities to obtain ${\hat{I}}_{c,2}$, the index set returned by SP algorithm. 3: Apply SVM-cone algorithm on the rows of ${\hat{U}}_{*,2}$ with *K* row communities to obtain ${\hat{I}}_{r,2}$, the index set returned by SVM-cone algorithm. 4: Set ${\hat{J}}_{*,2}=\mathrm{diag}\left({\hat{U}}_{*}\left({\hat{I}}_{r,2},:\right)\hat{\Lambda }{\hat{V}}^{\prime }\left({\hat{I}}_{c,2},:\right)\right),{\hat{Y}}_{*,2}={\hat{U}}_{2}{\hat{U}}_{*,2}^{\prime }\left({\hat{I}}_{r,2},:\right){\left({\hat{U}}_{*,2}\left({\hat{I}}_{r,2},:\right){\hat{U}}_{*,2}^{\prime }\left({\hat{I}}_{r,2},:\right)\right)}^{-1},{\hat{Z}}_{r,2}={\hat{Y}}_{*,2}{\hat{J}}_{*,2}$ and ${\hat{Z}}_{c,2}={\hat{V}}_{2}{\hat{V}}_{2}^{\prime }\left({\hat{I}}_{c,2},:\right){\left({\hat{V}}_{2}\left({\hat{I}}_{c,2},:\right){\hat{V}}_{2}^{\prime }\left({\hat{I}}_{c,2},:\right)\right)}^{-1}$. Then, set ${\hat{Z}}_{r,2}=\max\left(0,{\hat{Z}}_{r,2}\right)$ and ${\hat{Z}}_{c,2}=\max\left(0,{\hat{Z}}_{c,2}\right)$. 5: Estimate $\Pi_{r}\left(i,:\right)$ by ${\hat{\Pi }}_{r,2}\left(i,:\right)={\hat{Z}}_{r,2}\left(i,:\right)/{∥{\hat{Z}}_{r,2}\left(i,:\right)∥}_{1},1\leq i\leq n_{r}$ and estimate $\Pi_{c}\left(j,:\right)$ by ${\hat{\Pi }}_{c,2}\left(j,:\right)={\hat{Z}}_{c,2}\left(j,:\right)/{∥{\hat{Z}}_{c,2}\left(j,:\right)∥}_{1},1\leq j\leq n_{c}$.

**Lemma A2.** *(Equivalence). For the empirical case, we have ${\hat{I}}_{r,2}\equiv {\hat{I}}_{r},{\hat{I}}_{c,2}\equiv {\hat{I}}_{c},{\hat{U}}_{*,2}\left({\hat{I}}_{r,2},:\right){\hat{U}}_{*,2}^{\prime }\left({\hat{I}}_{r,2},:\right)\equiv {\hat{U}}_{*}\left({\hat{I}}_{r},:\right){\hat{U}}_{*}^{\prime }\left({\hat{I}}_{r},:\right),{\hat{Y}}_{*,2}\equiv {\hat{Y}}_{*},{\hat{J}}_{*,2}\equiv {\hat{J}}_{*},{\hat{Z}}_{r,2}\equiv {\hat{Z}}_{r},{\hat{Z}}_{c,2}\equiv {\hat{Z}}_{c},{\hat{\Pi }}_{r,2}\equiv {\hat{\Pi }}_{r}$ and ${\hat{\Pi }}_{c,2}\equiv {\hat{\Pi }}_{c}$.*

**Proof.** For column nodes, Lemma 3.2 [27] gives ${\hat{I}}_{c}={\hat{I}}_{c,2}$ (i.e., SP algorithm will return the same indices on both $\hat{V}$ and ${\hat{V}}_{2}$.), which gives that ${\hat{V}}_{2}{\hat{V}}_{2}^{\prime }\left({\hat{I}}_{c,2},:\right)={\hat{V}}_{2}{\hat{V}}_{2}^{\prime }\left({\hat{I}}_{c},:\right)=\hat{V}{\hat{V}}^{\prime }{\left(\left(\hat{V}{\hat{V}}^{\prime }\right)\left({\hat{I}}_{c},:\right)\right)}^{\prime }=\hat{V}{\hat{V}}^{\prime }{\left(\hat{V}\left({\hat{I}}_{c},:\right){\hat{V}}^{\prime }\right)}^{\prime }=\hat{V}{\hat{V}}^{\prime }\hat{V}{\hat{V}}^{\prime }\left({\hat{I}}_{c},:\right)=\hat{V}{\hat{V}}^{\prime }\left({\hat{I}}_{c},:\right)$, and ${\hat{V}}_{2}\left({\hat{I}}_{c,2},:\right){\hat{V}}_{2}^{\prime }\left({\hat{I}}_{c,2},:\right)={\hat{V}}_{2}\left({\hat{I}}_{c},:\right){\hat{V}}_{2}^{\prime }\left({\hat{I}}_{c},:\right)=\hat{V}\left({\hat{I}}_{c},:\right){\hat{V}}^{\prime }{\left(\hat{V}\left({\hat{I}}_{c},:\right){\hat{V}}^{\prime }\right)}^{\prime }=\hat{V}\left({\hat{I}}_{c},:\right){\hat{V}}^{\prime }\left({\hat{I}}_{c},:\right)$. Therefore, we have ${\hat{Z}}_{c,2}={\hat{Z}}_{c},{\hat{\Pi }}_{c,2}={\hat{\Pi }}_{c}$. For row nodes, Lemma G.1 [26] guarantees that ${\hat{I}}_{r}={\hat{I}}_{r,2}$ (i.e., SVM-cone algorithm will return the same indices on both ${\hat{U}}_{*}$ and ${\hat{U}}_{*,2}$.), so immediately we have ${\hat{J}}_{*,2}={\hat{J}}_{*}$. Since ${\hat{U}}_{*,2}\left({\hat{I}}_{r,2},:\right)={\hat{U}}_{*,2}\left({\hat{I}}_{r},:\right)=N_{\hat{U}}\left({\hat{I}}_{r},{\hat{I}}_{r}\right){\hat{U}}_{2}\left({\hat{I}}_{r},:\right)=N_{\hat{U}}\left({\hat{I}}_{r},{\hat{I}}_{r}\right)\hat{U}\left({\hat{I}}_{r},:\right){\hat{U}}^{\prime }={\hat{U}}_{*}\left({\hat{I}}_{r},:\right){\hat{U}}^{\prime }$, we have ${\hat{U}}_{2}{\hat{U}}_{*,2}^{\prime }\left({\hat{I}}_{r,2},:\right)={\hat{U}}_{2}{\hat{U}}_{*,2}^{\prime }\left({\hat{I}}_{r},:\right)=\hat{U}{\hat{U}}^{\prime }\hat{U}{\hat{U}}_{*}^{\prime }\left({\hat{I}}_{r},:\right)=\hat{U}{\hat{U}}_{*}^{\prime }\left({\hat{I}}_{r},:\right)$ and ${\left({\hat{U}}_{*,2}\left({\hat{I}}_{r,2},:\right){\hat{U}}_{*,2}^{\prime }\left({\hat{I}}_{r,2},:\right)\right)}^{-1}={\left({\hat{U}}_{*,2}\left({\hat{I}}_{r},:\right){\hat{U}}_{*,2}^{\prime }\left({\hat{I}}_{r},:\right)\right)}^{-1}={\left({\hat{U}}_{*}\left({\hat{I}}_{r},:\right){\hat{U}}_{*}^{\prime }\left({\hat{I}}_{r},:\right)\right)}^{-1}$, which give that ${\hat{Y}}_{*,2}={\hat{Y}}_{*}$, and the claim follows immediately.    □

Lemma A2 guarantees that the DiMSC and DiMSC-equivalence return same estimations for both row and column nodes’s memberships. In this article, we introduce the DiMSC-equivalence algorithm since it is helpful to build a theoretical framework for DiMSC, see Remark A3 and A4 for detail.

## Appendix D. Basic Properties of Ω

**Lemma A3.** 
*Under $DiDCMM_{n_{r},n_{c}}\left(K,P,\Pi_{r},\Pi_{c},\Theta_{r}\right)$, we have*

$$\begin{array}{cc} \; & \frac{\theta_{r,\min}}{\theta_{r,\max}\sqrt{K\lambda_{1}\left(\Pi_{r}^{\prime }\Pi_{r}\right)}}\leq {∥U\left(i,:\right)∥}_{F}\leq \frac{\theta_{r,\max}}{\theta_{r,\min}\sqrt{\lambda_{K}\left(\Pi_{r}^{\prime }\Pi_{r}\right)}},\;1\leq i\leq n_{r},\\ \; & \sqrt{\frac{1}{K\lambda_{1}\left(\Pi_{c}^{\prime }\Pi_{c}\right)}}\leq {∥V\left(j,:\right)∥}_{F}\leq \sqrt{\frac{1}{\lambda_{K}\left(\Pi_{c}^{\prime }\Pi_{c}\right)}},\;1\leq j\leq n_{c}. \end{array}$$

**Proof.** Since $I=U^{\prime }U=U^{\prime }\left(I_{r},:\right)\Theta_{r}^{-1}\left(I_{r},I_{r}\right)\Pi_{r}^{\prime }\Theta_{r}^{2}\Pi_{r}\Theta^{-1}\left(I_{r},I_{r}\right)U\left(I_{r},:\right)$, we have

$$\begin{array}{c} (\left(\Theta_{r}^{-1}\left(I_{r},I_{r}\right)U\left(I_{r},:\right)\right){\left({\left(\Theta_{r}^{-1}\left(I_{r},I_{r}\right)U\left(I_{r},:\right)\right)}^{\prime }\right)}^{-1}=\Pi_{r}^{\prime }\Theta_{r}^{2}\Pi_{r}, \end{array}$$

which gives that

$$\begin{array}{cc} \max_{k}{∥e_{k}^{\prime }\left(\Theta_{r}^{-1}\left(I_{r},I_{r}\right)U\left(I_{r},:\right)\right)∥}_{F}^{2} & =\max_{k}e_{k}^{\prime }\left(\Theta_{r}^{-1}\left(I_{r},I_{r}\right)U\left(I_{r},:\right)\right){\left(\Theta_{r}^{-1}\left(I_{r},I_{r}\right)U\left(I_{r},:\right)\right)}^{\prime }e_{k}\\ \; & \leq \max_{{∥x∥}_{F}=1}x^{\prime }\left(\Theta_{r}^{-1}\left(I_{r},I_{r}\right)U\left(I_{r},:\right)\right){\left(\Theta_{r}^{-1}\left(I_{r},I_{r}\right)U\left(I_{r},:\right)\right)}^{\prime }x\\ \; & =\lambda_{1}\left(\left(\Theta_{r}^{-1}\left(I_{r},I_{r}\right)U\left(I_{r},:\right)\right){\left(\Theta_{r}^{-1}\left(I_{r},I_{r}\right)U\left(I_{r},:\right)\right)}^{\prime }\right)\\ \; & =\frac{1}{\lambda_{K}\left(\Pi_{r}^{\prime }\Theta_{r}^{2}\Pi_{r}\right)}\leq \frac{1}{\theta_{r,\min}^{2}\lambda_{K}\left(\Pi_{r}^{\prime }\Pi_{r}\right)}. \end{array}$$

Similarly, we have

$$\begin{array}{c} \min_{k}{∥e_{k}^{\prime }\left(\Theta_{r}^{-1}\left(I_{r},I_{r}\right)U\left(I_{r},:\right)\right)∥}_{F}^{2}\geq \frac{1}{\lambda_{1}\left(\Pi_{r}^{\prime }\Theta_{r}^{2}\Pi_{r}\right)}\geq \frac{1}{\theta_{r,\max}^{2}\lambda_{1}\left(\Pi_{r}^{\prime }\Pi_{r}\right)}. \end{array}$$

Since $U\left(i,:\right)=e_{i}^{\prime }U=e_{i}^{\prime }\Theta_{r}\Pi_{r}\Theta_{r}^{-1}\left(I_{r},I_{r}\right)U\left(I_{r},:\right)=\theta_{r}\left(i\right)\Pi_{r}\left(i,:\right)\Theta_{r}^{-1}\left(I_{r},I_{r}\right)U\left(I_{r},:\right)$ for $1\leq i\leq n_{r}$, we have

$$\begin{array}{cc} {∥U\left(i,:\right)∥}_{F} & =∥\theta_{r}\left(i\right)\Pi_{r}\left(i,:\right)\Theta_{r}^{-1}\left(I_{r},I_{r}\right)U\left(I_{r},:\right){∥}_{F}=\theta_{r}\left(i\right){∥\Pi_{r}\left(i,:\right)\Theta_{r}^{-1}\left(I_{r},I_{r}\right)U\left(I_{r},:\right)∥}_{F}\\ \; & \leq \theta_{r}\left(i\right)\max_{i}∥\Pi_{r}{\left(i,:\right)∥}_{F}\max_{i}{∥e_{i}^{\prime }\left(\Theta_{r}^{-1}\left(I_{r},I_{r}\right)U\left(I_{r},:\right)\right)∥}_{F}\\ \; & \leq \theta_{r}\left(i\right)\max_{i}{∥e_{i}^{\prime }\left(\Theta_{r}^{-1}\left(I_{r},I_{r}\right)U\left(I_{r},:\right)\right)∥}_{F}\leq \frac{\theta_{r,\max}}{\theta_{r,\min}\sqrt{\lambda_{K}\left(\Pi_{r}^{\prime }\Pi_{r}\right)}}. \end{array}$$

Similarly, we have

$$\begin{array}{cc} {∥U\left(i,:\right)∥}_{F} & \geq \theta_{r}\left(i\right)\min_{i}∥\Pi_{r}{\left(i,:\right)∥}_{F}\min_{i}{∥e_{i}^{\prime }\left(\Theta_{r}^{-1}\left(I_{r},I_{r}\right)U\left(I_{r},:\right)\right)∥}_{F}\\ \; & \geq \theta_{r}\left(i\right)\min_{i}{∥e_{i}^{\prime }\left(\Theta_{r}^{-1}\left(I_{r},I_{r}\right)U\left(I_{r},:\right)\right)∥}_{F}/\sqrt{K}\geq \frac{\theta_{r,\min}}{\theta_{r,\max}\sqrt{K\lambda_{1}\left(\Pi_{r}^{\prime }\Pi_{r}\right)}}. \end{array}$$

For ${∥V\left(j,:\right)∥}_{F}$, since $V=\Pi_{c}B_{c}$, we have

$$\begin{array}{cc} \min_{j}{∥e_{j}^{\prime }V∥}_{F}^{2} & =\min_{j}e_{j}^{\prime }VV^{\prime }e_{j}=\min_{j}\Pi_{c}\left(j,:\right)B_{c}B_{c}^{\prime }\Pi_{c}^{\prime }\left(j,:\right)\\ \; & =\min_{j}{∥\Pi_{c}\left(j,:\right)∥}_{F}^{2}\frac{\Pi_{c}\left(j,:\right)}{∥\Pi_{c}{\left(j,:\right)∥}_{F}}B_{c}B_{c}^{\prime }\frac{\Pi_{c}^{\prime }\left(j,:\right)}{∥\Pi_{c}{\left(j,:\right)∥}_{F}}\\ \; & \geq \min_{j}∥\Pi_{c}{\left(j,:\right)∥}_{F}^{2}\min_{{∥x∥}_{F}=1}x^{\prime }B_{c}B_{c}^{\prime }x=\min_{j}{∥\Pi_{c}\left(j,:\right)∥}_{F}^{2}\lambda_{K}\left(B_{c}B_{c}^{\prime }\right)\\ \; & \overset{\mathrm{By}\;\mathrm{Lemma}A4\;}{=}\frac{\min_{j}{∥\Pi_{c}\left(j,:\right)∥}_{F}^{2}}{\lambda_{1}\left(\Pi_{c}^{\prime }\Pi_{c}\right)}\geq \frac{1}{K\lambda_{1}\left(\Pi_{c}^{\prime }\Pi_{c}\right)}. \end{array}$$

Meanwhile,

$$\begin{array}{cc} \max_{j}{∥e_{j}^{\prime }V∥}_{F}^{2} & =\max_{j}{∥\Pi_{c}\left(j,:\right)∥}_{F}^{2}\frac{\Pi_{c}\left(j,:\right)}{∥\Pi_{c}{\left(j,:\right)∥}_{F}}B_{c}B_{c}^{\prime }\frac{\Pi_{c}^{\prime }\left(j,:\right)}{∥\Pi_{c}{\left(j,:\right)∥}_{F}}\\ \; & \leq \max_{j}∥\Pi_{c}{\left(j,:\right)∥}_{F}^{2}\max_{{∥x∥}_{F}=1}x^{\prime }B_{c}B_{c}^{\prime }x=\max_{j}{∥\Pi_{c}\left(j,:\right)∥}_{F}^{2}\lambda_{K}\left(B_{c}B_{c}^{\prime }\right)\\ \; & \overset{\mathrm{By}\;\mathrm{Lemma}A4\;}{=}\frac{\max_{j}{∥\Pi_{c}\left(j,:\right)∥}_{F}^{2}}{\lambda_{K}\left(\Pi_{c}^{\prime }\Pi_{c}\right)}\leq \frac{1}{\lambda_{K}\left(\Pi_{c}^{\prime }\Pi_{c}\right)}. \end{array}$$

   □

**Lemma A4.** 
*Under $DiDCMM_{n_{r},n_{c}}\left(K,P,\Pi_{r},\Pi_{c},\Theta_{r}\right)$, we have*

$$\begin{array}{cc} \; & \frac{\theta_{r,\min}^{2}\lambda_{K}\left(\Pi_{r}^{\prime }\Pi_{r}\right)}{\theta_{r,\max}^{2}\lambda_{1}\left(\Pi_{r}^{\prime }\Pi_{r}\right)}\leq \lambda_{K}\left(U_{*}\left(I_{r},:\right)U_{*}^{\prime }\left(I_{r},:\right)\right),\lambda_{1}\left(U_{*}\left(I_{r},:\right)U_{*}^{\prime }\left(I_{r},:\right)\right)\leq \frac{\theta_{r,\max}^{2}K\lambda_{1}\left(\Pi_{r}^{\prime }\Pi_{r}\right)}{\theta_{r,\min}^{2}\lambda_{K}\left(\Pi_{r}^{\prime }\Pi_{r}\right)},\\ \; & \mathrm{and}\;\lambda_{1}\left(B_{c}B_{c}^{\prime }\right)=\frac{1}{\lambda_{K}\left(\Pi_{c}^{\prime }\Pi_{c}\right)},\lambda_{K}\left(B_{c}B_{c}^{\prime }\right)=\frac{1}{\lambda_{1}\left(\Pi_{c}^{\prime }\Pi_{c}\right)}. \end{array}$$

**Proof.** Recall that $V=\Pi_{c}B_{c}$ and $V^{\prime }V=I$, we have $I=B_{c}^{\prime }\Pi_{c}^{\prime }\Pi_{c}B_{c}$. As $B_{c}$ is full rank, we have $\Pi_{c}^{\prime }\Pi_{c}={\left(B_{c}B_{c}^{\prime }\right)}^{-1}$, which gives

$$\begin{array}{c} \lambda_{1}\left(B_{c}B_{c}^{\prime }\right)=\frac{1}{\lambda_{K}\left(\Pi_{c}^{\prime }\Pi_{c}\right)},\lambda_{K}\left(B_{c}B_{c}^{\prime }\right)=\frac{1}{\lambda_{1}\left(\Pi_{c}^{\prime }\Pi_{c}\right)}. \end{array}$$

By the proof of Lemma 2, we know that

$$\begin{array}{c} {\left(U_{*}\left(I_{r},:\right)U_{*}^{\prime }\left(I_{r},:\right)\right)}^{-1}=N_{U}^{-1}\left(I_{r},I_{r}\right)\Theta^{-1}\left(I_{r},I_{r}\right)\Pi_{r}^{\prime }\Theta_{r}^{2}\Pi_{r}\Theta_{r}^{-1}\left(I_{r},I_{r}\right)N_{U}^{-1}\left(I_{r},I_{r}\right), \end{array}$$

which gives that

$$\begin{array}{c} U_{*}\left(I_{r},:\right)U_{*}^{\prime }\left(I_{r},:\right)=N_{U}\left(I_{r},I_{r}\right)\Theta \left(I_{r},I_{r}\right){\left(\Pi_{r}^{\prime }\Theta_{r}^{2}\Pi_{r}\right)}^{-1}\Theta_{r}\left(I_{r},I_{r}\right)N_{U}\left(I_{r},I_{r}\right). \end{array}$$

Then, we have

$$\begin{array}{cc} & \lambda_{1}\left(U_{*}\left(I_{r},:\right)U_{*}^{\prime }\left(I_{r},:\right)\right)=\lambda_{1}\left(N_{U}\left(I_{r},I_{r}\right)\Theta \left(I_{r},I_{r}\right){\left(\Pi_{r}^{\prime }\Theta_{r}^{2}\Pi_{r}\right)}^{-1}\Theta_{r}\left(I_{r},I_{r}\right)N_{U}\left(I_{r},I_{r}\right)\right)\\ \; & =\lambda_{1}\left(N_{U}^{2}\left(I_{r},I_{r}\right)\Theta_{r}^{2}\left(I_{r},I_{r}\right){\left(\Pi_{r}^{\prime }\Theta_{r}^{2}\Pi_{r}\right)}^{-1}\right)\leq \lambda_{1}^{2}\left(N_{U}\left(I_{r},I_{r}\right)\Theta_{r}\left(I_{r},I_{r}\right)\right)\lambda_{1}\left({\left(\Pi_{r}^{\prime }\Theta_{r}^{2}\Pi_{r}\right)}^{-1}\right)\\ & =\lambda_{1}^{2}\left(N_{U}\left(I_{r},I_{r}\right)\Theta_{r}\left(I_{r},I_{r}\right)\right)/\lambda_{K}\left(\Pi_{r}^{\prime }\Theta_{r}^{2}\Pi_{r}\right)\leq (\max_{i\in I_{r}}\theta_{r}\left(i\right)/∥U\left(i,:\right){{∥}_{F})}^{2}/\lambda_{K}\left(\Pi_{r}^{\prime }\Theta_{r}^{2}\Pi_{r}\right)\\ & \leq \frac{\theta_{r,\max}^{2}K\lambda_{1}\left(\Pi_{r}^{\prime }\Pi_{r}\right)}{\lambda_{K}\left(\Pi_{r}^{\prime }\Theta_{r}^{2}\Pi_{r}\right)}\leq \frac{\theta_{r,\max}^{2}K\lambda_{1}\left(\Pi_{r}^{\prime }\Pi_{r}\right)}{\theta_{r,\min}^{2}\lambda_{K}\left(\Pi_{r}^{\prime }\Pi_{r}\right)}. \end{array}$$

Similarly, we have

$$\begin{array}{cc} & \lambda_{K}\left(U_{*}\left(I_{r},:\right)U_{*}^{\prime }\left(I_{r},:\right)\right)=\lambda_{K}\left(N_{U}\left(I_{r},I_{r}\right)\Theta \left(I_{r},I_{r}\right){\left(\Pi_{r}^{\prime }\Theta_{r}^{2}\Pi_{r}\right)}^{-1}\Theta_{r}\left(I_{r},I_{r}\right)N_{U}\left(I_{r},I_{r}\right)\right)\\ \; & =\lambda_{K}\left(N_{U}^{2}\left(I_{r},I_{r}\right)\Theta_{r}^{2}\left(I_{r},I_{r}\right){\left(\Pi_{r}^{\prime }\Theta_{r}^{2}\Pi_{r}\right)}^{-1}\right)\geq \lambda_{K}^{2}\left(N_{U}\left(I_{r},I_{r}\right)\Theta_{r}\left(I_{r},I_{r}\right)\right)\lambda_{K}\left({\left(\Pi_{r}^{\prime }\Theta_{r}^{2}\Pi_{r}\right)}^{-1}\right)\\ & =\lambda_{K}^{2}\left(N_{U}\left(I_{r},I_{r}\right)\Theta_{r}\left(I_{r},I_{r}\right)\right)/\lambda_{1}\left(\Pi_{r}^{\prime }\Theta_{r}^{2}\Pi_{r}\right)\geq (\min_{i\in I_{r}}\theta_{r}\left(i\right)/∥U\left(i,:\right){{∥}_{F})}^{2}/\lambda_{1}\left(\Pi_{r}^{\prime }\Theta_{r}^{2}\Pi_{r}\right)\\ & \geq \frac{\theta_{r,\min}^{2}\lambda_{K}\left(\Pi_{r}^{\prime }\Pi_{r}\right)}{\lambda_{1}\left(\Pi_{r}^{\prime }\Theta_{r}^{2}\Pi_{r}\right)}\geq \frac{\theta_{r,\min}^{2}\lambda_{K}\left(\Pi_{r}^{\prime }\Pi_{r}\right)}{\theta_{r,\max}^{2}\lambda_{1}\left(\Pi_{r}^{\prime }\Pi_{r}\right)}. \end{array}$$

   □

**Lemma A5.** 
*Under $DiDCMM_{n_{r},n_{c}}\left(K,P,\Pi_{r},\Pi_{c},\Theta_{r}\right)$, we have*

$$\begin{array}{c} \sigma_{K}\left(\Omega \right)\geq \theta_{r,\min}\sigma_{K}\left(P\right)\sigma_{K}\left(\Pi_{r}\right)\sigma_{K}\left(\Pi_{c}\right),\sigma_{1}\left(\Omega \right)\leq \theta_{r,\max}\sigma_{1}\left(P\right)\sigma_{1}\left(\Pi_{r}\right)\sigma_{1}\left(\Pi_{c}\right). \end{array}$$

**Proof.** For $\sigma_{K}\left(\Omega \right)$, we have

$$\begin{array}{cc} \; & \sigma_{K}^{2}\left(\Omega \right)=\lambda_{K}\left(\Omega \Omega^{\prime }\right)=\lambda_{K}\left(\Theta_{r}\Pi_{r}P\Pi_{c}^{\prime }\Pi_{c}P^{\prime }\Pi_{r}^{\prime }\Theta_{r}\right)=\lambda_{K}\left(\Theta_{r}^{2}\Pi_{r}P\Pi_{c}^{\prime }\Pi_{c}P^{\prime }\Pi_{r}^{\prime }\right)\\ \; & \geq \theta_{r,\min}^{2}\lambda_{K}\left(\Pi_{r}^{\prime }\Pi_{r}P\Pi_{c}^{\prime }\Pi_{c}P^{\prime }\right)\geq \theta_{r,\min}^{2}\lambda_{K}\left(\Pi_{r}^{\prime }\Pi_{r}\right)\lambda_{K}\left(P\Pi_{c}^{\prime }\Pi_{c}P^{\prime }\right)=\theta_{r,\min}^{2}\lambda_{K}\left(\Pi_{r}^{\prime }\Pi_{r}\right)\lambda_{K}\left(\Pi_{c}^{\prime }\Pi_{c}P^{\prime }P\right)\\ \; & \geq \theta_{r,\min}^{2}\lambda_{K}\left(\Pi_{r}^{\prime }\Pi_{r}\right)\lambda_{K}\left(\Pi_{c}^{\prime }\Pi_{c}\right)\lambda_{K}\left(PP^{\prime }\right)=\theta_{r,\min}^{2}\sigma_{K}^{2}\left(\Pi_{r}\right)\sigma_{K}^{2}\left(\Pi_{c}\right)\sigma_{K}^{2}\left(P\right), \end{array}$$

where we have used the fact for any matrices X,Y, the nonzero eigenvalues of XY are the same as the nonzero eigenvalues of YX. Following a similar analysis, the lemma follows.    □

**Lemma A6.** 
*Under $DiDCMM_{n_{r},n_{c}}\left(K,P,\Pi_{r},\Pi_{c},\Theta_{r}\right)$, when Assumption A1 holds, with probability at least $1-o({\left(n_{r}+n_{c}\right)}^{-3})$, we have*

$$\begin{array}{c} ∥A-\Omega ∥=O(\sqrt{P_{\max}\max(∥\theta_{r}{∥}_{1},\theta_{r,\max}n_{c})\log\left(n_{r}+n_{c}\right)}). \end{array}$$

**Proof.** Since the proof is similar to that of Lemma 7 [35], we omit most of the details. Let $e_{i}$ be an $n_{r}\times 1$ vector, where $e_{i}\left(i\right)=1$ and 0 elsewhere, for row nodes $1\leq i\leq n_{r}$, and ${\tilde{e}}_{j}$ be an $n_{c}\times 1$ vector, where ${\tilde{e}}_{j}\left(j\right)=1$ and 0 elsewhere, for column nodes $1\leq j\leq n_{c}$. Set $W=\sum_{i=1}^{n^{r}}\sum_{j=1}^{n_{c}}W\left(i,j\right)e_{i}{\tilde{e}}_{j}^{\prime }$, where W=A−Ω. Set $W^{\left(i,j\right)}=W\left(i,j\right)e_{i}{\tilde{e}}_{j}^{\prime }$, for $1\leq i\leq n_{r},1\leq j\leq n_{c}$. Then, we have $E(W^{\left(i,j\right)})=0$. For $1\leq i\leq n_{r},1\leq j\leq n_{c}$, we have

$$\begin{array}{cc} ∥W^{\left(i,j\right)}∥ & =∥W\left(i,j\right)e_{i}{\tilde{e}}_{j}^{\prime }∥=|A\left(i,j\right)-\Omega \left(i,j\right)|\leq 1. \end{array}$$

Next, we consider the variance parameter

$$\begin{array}{c} \sigma^{2}:=\max(∥\sum_{i=1}^{n_{r}}\sum_{j=1}^{n_{c}}E\left(W^{\left(i,j\right)}{\left(W^{\left(i,j\right)}\right)}^{\prime }\right)∥,∥\sum_{i=1}^{n_{r}}\sum_{j=1}^{n_{c}}E\left({\left(W^{\left(i,j\right)}\right)}^{\prime }W^{\left(i,j\right)}\right)∥). \end{array}$$

Since

$$\begin{array}{c} E\left(W^{2}\left(i,j\right)\right)=E\left({\left(A\left(i,j\right)-\Omega \left(i,j\right)\right)}^{2}\right)=\mathrm{Var}\left(A\left(i,j\right)\right), \end{array}$$

where Var(A(i,j)) denotes the variance of the Bernoulli random variable A(i,j), we have

$$\begin{array}{cc} E(W^{2}\left(i,j\right)) & =\mathrm{Var}(A(i,j))=P(A(i,j)=1)(1-P(A(i,j)=1))\\ \; & \leq P\left(A\left(i,j\right)=1\right)=\Omega \left(i,j\right)=e_{i}^{\prime }\Theta_{r}\Pi_{r}P\Pi_{c}^{\prime }{\tilde{e}}_{j}=\theta_{r}\left(i\right)e_{i}^{\prime }\Pi_{r}P\Pi_{c}^{\prime }{\tilde{e}}_{j}\leq \theta_{r}\left(i\right)P_{\max}. \end{array}$$

Since $e_{i}e_{i}^{\prime }$ is an $n_{r}\times n_{r}$ diagonal matrix with (i,i)-th entry being one and other entries being zero, we have

$$\begin{array}{c} ∥\sum_{i=1}^{n_{r}}\sum_{j=1}^{n_{c}}E\left(W^{\left(i,j\right)}{\left(W^{\left(i,j\right)}\right)}^{\prime }\right)∥=∥\sum_{i=1}^{n_{r}}\sum_{j=1}^{n_{c}}E\left(W^{2}\left(i,j\right)\right)e_{i}e_{i}^{\prime }∥=\max_{1\leq i\leq n_{r}}\left|\sum_{j=1}^{n_{c}}E\left(W^{2}\left(i,j\right)\right)\right|\leq \theta_{r,\max}P_{\max}n_{c}. \end{array}$$

Similarly, we have $∥\sum_{i=1}^{n_{r}}\sum_{j=1}^{n_{c}}E\left({\left(W^{\left(i,j\right)}\right)}^{\prime }W^{\left(i,j\right)}\right)∥\leq P_{\max}{∥\theta_{r}∥}_{1}$, which gives that

$$\begin{array}{c} \sigma^{2}=\max(∥\sum_{i=1}^{n_{r}}\sum_{j=1}^{n_{c}}E\left(W^{\left(i,j\right)}{\left(W^{\left(i,j\right)}\right)}^{\prime }\right)∥,∥\sum_{i=1}^{n_{r}}\sum_{j=1}^{n_{c}}E\left({\left(W^{\left(i,j\right)}\right)}^{\prime }W^{\left(i,j\right)}\right)∥)\leq P_{\max}\max(∥\theta_{r}{∥}_{1},\theta_{r,\max}n_{c}). \end{array}$$

By the rectangular version of the Bernstein inequality [71], combining with $\sigma^{2}\leq P_{\max}\max(∥\theta_{r}{∥}_{1},\theta_{r,\max}n_{c}),R=1,d_{1}+d_{2}=n_{r}+n_{c}$, set $t=\frac{\alpha +1+\sqrt{\alpha^{2}+20\alpha +19}}{3}\sqrt{P_{\max}\max(∥\theta_{r}{∥}_{1},\theta_{r,\max}n_{c})\log\left(n_{r}+n_{c}\right)}$ for any α>0, we have

$$\begin{array}{cc} \; & P(∥W∥\geq t)=P(∥\sum_{i=1}^{n^{r}}\sum_{j=1}^{n_{c}}W^{\left(i,j\right)}∥\geq t)\leq \left(n_{r}+n_{c}\right)\exp\left(-\frac{t^{2}/2}{\sigma^{2}+\frac{Rt}{3}}\right)\\ \; & \leq \left(n_{r}+n_{c}\right)\exp\left(-\frac{t^{2}/2}{P_{\max}\max(∥\theta_{r}{∥}_{1},\theta_{r,\max}n_{c})+t/3}\right)\\ \; & =\left(n_{r}+n_{c}\right)\exp\left(-\left(\alpha +1\right)\log\left(n_{r}+n_{c}\right)\cdot \frac{1}{\frac{18}{{\left(\sqrt{\alpha +19}+\sqrt{\alpha +1}\right)}^{2}}+\frac{2\sqrt{\alpha +1}}{\sqrt{\alpha +19}+\sqrt{\alpha +1}}\sqrt{\frac{\log(n_{r}+n_{c})}{P_{\max}\max(∥\theta_{r}{∥}_{1},\theta_{r,\max}n_{c})}}}\right)\\ \; & \leq \left(n_{r}+n_{c}\right)\exp\left(-\left(\alpha +1\right)\log\left(n_{r}+n_{c}\right)\right)=\frac{1}{{\left(n_{r}+n_{c}\right)}^{\alpha }}, \end{array}$$

where we have used Assumption 1 in the last inequality. Set α=3, and the claim follows.    □

## Appendix E. Proof of Consistency for DiMSC

Similar to [24,26,27], for our DiMSC, the main theoretical results (i.e., Theorem 2) rely on the row-wise singular vector deviation bounds for the singular eigenvectors of the adjacency matrix.

**Lemma A7.** *(Row-wise singular vector deviation) Under $DiDCMM_{n_{r},n_{c}}\left(K,P,\Pi_{r},\Pi_{c},\Theta_{r}\right)$, when Assumption 1 holds, suppose $\sigma_{K}\left(\Omega \right)\geq C\sqrt{\theta_{r,\max}\left(n_{r}+n_{c}\right)\log\left(n_{r}+n_{c}\right)}$, with probability at least $1-o({\left(n_{r}+n_{c}\right)}^{-3})$, we have*

$$\begin{array}{cc} \; & \max(∥\hat{U}{\hat{U}}^{\prime }-UU^{\prime }{∥}_{2\to \infty },∥\hat{V}{\hat{V}}^{\prime }-VV^{\prime }{∥}_{2\to \infty })=O\left(\frac{\sqrt{P_{\max}\theta_{r,\max}K\log\left(n_{r}+n_{c}\right)}}{\theta_{r,\min}\sigma_{K}\left(P\right)\sigma_{K}\left(\Pi_{r}\right)\sigma_{K}\left(\Pi_{c}\right)}\right). \end{array}$$

**Proof.** Let $H_{\hat{U}}={\hat{U}}^{\prime }U$, and $H_{\hat{U}}=U_{H_{\hat{U}}}\Sigma_{H_{\hat{U}}}V_{H_{\hat{U}}}^{\prime }$ be the SVD decomposition of $H_{\hat{U}}$ with $U_{H_{\hat{U}}},V_{H_{\hat{U}}}\in R^{n_{r}\times K}$, where $U_{H_{\hat{U}}}$ and $V_{H_{\hat{U}}}$ represent, respectively, the left and right singular matrices of $H_{\hat{U}}$. Define $\mathrm{sgn}\left(H_{\hat{U}}\right)=U_{H_{\hat{U}}}V_{H_{\hat{U}}}^{\prime }$; $\mathrm{sgn}(H_{\hat{V}})$ is defined similarly. Since E(A(i,j)−Ω(i,j))=0, $E\left[{\left(A\left(i,j\right)-\Omega \left(i,j\right)\right)}^{2}\right]\leq \theta_{r}\left(i\right)P_{\max}\leq \theta_{r,\max}P_{\max}$ by the proof of Lemma A6, $\frac{1}{\sqrt{\theta_{r,\max}P_{\max}\min\left(n_{r},n_{c}\right)/\left(\mu \log\left(n_{r}+n_{c}\right)\right)}}\leq O\left(1\right)$ holds by Assumption 1, where μ is the incoherence parameter defined as $\mu =\max(\frac{n_{r}{∥U∥}_{2\to \infty }^{2}}{K},\frac{n_{c}{∥V∥}_{2\to \infty }^{2}}{K})$. By Theorem 4.4 [64], with high probability, we have below row-wise singular vector deviation

(A1) $$\begin{array}{cc} \; & \max(∥\hat{U}\mathrm{sgn}\left(H_{\hat{U}}\right)-U{∥}_{2\to \infty },∥\hat{V}\mathrm{sgn}\left(H_{\hat{V}}\right)-V{∥}_{2\to \infty })\\ \; & \leq C\frac{\sqrt{P_{\max}\theta_{r,\max}K}\left(\kappa \left(\Omega \right)\sqrt{\frac{\max(n_{r},n_{c})\mu }{\min(n_{r},n_{c})}}+\sqrt{\log(n_{r}+n_{c})}\right)}{\sigma_{K}\left(\Omega \right)}\\ \; & \leq C\frac{\sqrt{P_{\max}\theta_{r,\max}K\log\left(n_{r}+n_{c}\right)}}{\sigma_{K}\left(\Omega \right)}, \end{array}$$

provided that $c_{1}\sigma_{K}\left(\Omega \right)\geq \sqrt{\theta_{r,\max}P_{\max}\left(n_{r}+n_{c}\right)\log\left(n_{r}+n_{c}\right)}$ for some sufficiently small constant $c_{1}$, and here we set $\sqrt{\frac{\max(n_{r},n_{c})\mu }{\min(n_{r},n_{c})}}=O\left(1\right)$ for convenience since this term has little effect on the error bounds of DiMSC, especially for the case when $\frac{n_{r}}{n_{c}}=O\left(1\right)$. Since $U^{\prime }U=I,{\hat{U}}^{\prime }\hat{U}=I$, we have $∥\hat{U}{\hat{U}}^{\prime }-UU^{\prime }{∥}_{2\to \infty }\leq 2{∥U-\hat{U}\mathrm{sgn}\left(H_{\hat{U}}\right)∥}_{2\to \infty }$ by basic algebra. Now, we are ready to bound $∥\hat{U}{\hat{U}}^{\prime }-UU^{\prime }{∥}_{2\to \infty }$:

$$\begin{array}{cc} \; & ∥\hat{U}{\hat{U}}^{\prime }-UU^{\prime }{∥}_{2\to \infty }=\max_{1\leq i\leq n_{r}}∥e_{i}^{\prime }\left(UU^{\prime }-\hat{U}{\hat{U}}^{\prime }\right){∥}_{F}\leq 2{∥U-\hat{U}\mathrm{sgn}\left(H_{\hat{U}}\right)∥}_{2\to \infty }\\ \; & \leq C\frac{\sqrt{P_{\max}\theta_{r,\max}K\log\left(n_{r}+n_{c}\right)}}{\sigma_{K}\left(\Omega \right)}\overset{\mathrm{By}\;\mathrm{Lemma}A5\;}{\leq }C\frac{\sqrt{P_{\max}\theta_{r,\max}K\log\left(n_{r}+n_{c}\right)}}{\theta_{r,\min}\sigma_{K}\left(P\right)\sigma_{K}\left(\Pi_{r}\right)\sigma_{K}\left(\Pi_{c}\right)}. \end{array}$$

The lemma holds by following similar proof for $∥\hat{V}{\hat{V}}^{\prime }-VV^{\prime }{∥}_{2\to \infty }$.    □

When $\Theta_{r}=\rho I,n_{r}=n_{c},\Pi_{r}=\Pi_{c}=\Pi ,P_{\max}=O\left(1\right)$, and DiCCMM degenerates to MMSB, the bound in Lemma A7 is $O(\frac{\sqrt{K\log(n)}}{\sigma_{K}\left(P\right)\sqrt{\rho }\lambda_{K}\left(\Pi^{\prime }\Pi \right)})$. if we further assume that $\lambda_{K}\left(\Pi^{\prime }\Pi \right)=O\left(\frac{n}{K}\right)$ and K=O(1), the bound is of order $O(\frac{1}{\sigma_{K}\left(P\right)}\frac{1}{\sqrt{n}}\sqrt{\frac{\log(n)}{\rho n}})$. Set the Θ in [24] as $\sqrt{\rho }I$, their DCMM degenerates to MMSB, their assumptions are translated to our $\lambda_{K}\left(\Pi^{\prime }\Pi \right)=O\left(\frac{n}{K}\right)$, when K=O(1), the row-wise singular vector deviation bound in the fourth bullet of Lemma 2.1 [24] is $O(\frac{1}{\sigma_{K}\left(P\right)}\frac{1}{\sqrt{n}}\sqrt{\frac{\log(n)}{\rho n}})$, which is consistent with ours. Meanwhile, if we further assume that $\sigma_{K}\left(P\right)=O\left(1\right)$, the bound is of order $\frac{1}{\sqrt{n}}\sqrt{\frac{\log(n)}{\rho n}}$.

The next lemma is the cornerstone to characterizing the behaviors of DiMSC.

**Lemma A8.** 
*Under $DiDCMM_{n_{r},n_{c}}\left(K,P,\Pi_{r},\Pi_{c},\Theta_{r}\right)$, when conditions of Lemma A7 hold, there exist two permutation matrices $P_{r},P_{c}\in R^{K\times K}$ such that with probability at least $1-o({\left(n_{r}+n_{c}\right)}^{-3})$, we have*

$$\begin{array}{cc} \; & \max_{1\leq k\leq K}{∥e_{k}^{\prime }\left({\hat{U}}_{*,2}\left({\hat{I}}_{r},:\right)-P_{r}^{\prime }U_{*,2}\left(I_{r},:\right)\right)∥}_{F}=O\left(\frac{K^{3}\theta_{r,\max}^{11}\varpi \kappa^{3}\left(\Pi_{r}^{\prime }\Pi_{r}\right)\lambda_{1}^{1.5}\left(\Pi_{r}^{\prime }\Pi_{r}\right)}{\theta_{r,\min}^{11}\pi_{r,\min}}\right),\\ \; & \max_{1\leq k\leq K}{∥e_{k}^{\prime }\left({\hat{V}}_{2}\left({\hat{I}}_{c},:\right)-P_{c}^{\prime }V_{2}\left(I_{c},:\right)\right)∥}_{F}=O\left(\varpi \kappa \left(\Pi_{c}^{\prime }\Pi_{c}\right)\right). \end{array}$$

**Proof.** First, we consider column nodes. The detail of the SP algorithm is in Algorithm A2.

**Algorithm A2** Successive Projection (SP) [54]
**Require:** Near-separable matrix $Y_{sp}=S_{sp}M_{sp}+Z_{sp}\in R_{+}^{m\times n}$, where $S_{sp},M_{sp}$ should satisfy Assumption 1 [54], the number *r* of columns to be extracted. **Ensure:** Set of indices K such that Y(K,:)≈S (up to permutation) 1: Compute ${\hat{U}}_{r}\in R^{n_{r}\times K_{r}}$ and ${\hat{U}}_{c}\in R^{n_{c}\times K_{r}}$ from the top-$K_{r}$-dimensional SVD of *A*. 2: Let $R=Y_{sp},K=\left\{\right\},k=1$. 3: **While**R≠0 and k≤r **do** 4: $k_{*}={\mathrm{argmax}}_{k}{∥R\left(k,:\right)∥}_{F}$. 5: $u_{k}=R\left(k_{*},:\right)$. 6: $R\leftarrow (I-\frac{u_{k}u_{k}^{\prime }}{∥u_{k}{∥}_{F}^{2}})R$. 7: $K=K\cup \{k_{*}\}$. 8: k=k+1. 9: **end while**

Based on Algorithm A2, the following theorem is Theorem 1.1 in [54]. **Theorem A1.** *Fix m≥r and n≥r. Consider a matrix $Y_{sp}=S_{sp}M_{sp}+Z_{sp}$, where $S_{sp}\in R^{m\times r}$ has a full column rank, $M_{sp}\in R^{r\times n}$ is a nonnegative matrix such that the sum of each column is at most 1, and $Z_{sp}=\left[Z_{sp,1},\ldots ,Z_{sp,n}\right]\in R^{m\times n}$. Suppose $M_{sp}$ has a submatrix equal to $I_{r}$. Write $\epsilon \leq \max_{1\leq i\leq n}{∥Z_{sp,i}∥}_{F}$. Suppose $\epsilon =O(\frac{\sigma_{\min}\left(S_{sp}\right)}{\sqrt{r}\kappa^{2}\left(S_{sp}\right)})$, where $\sigma_{\min}\left(S_{sp}\right)$ and $\kappa (S_{sp})$ are the minimum singular value and condition number of $S_{sp}$, respectively. If we apply the SP algorithm to columns of $Y_{sp}$, then it outputs an index set K⊂{1,2,…,n} such that |K|=r and $\max_{1\leq k\leq r}\min_{j\in K}{∥S_{sp}\left(:,k\right)-Y_{sp}\left(:,j\right)∥}_{F}=O\left(\epsilon \kappa^{2}\left(S_{sp}\right)\right)$, where $S_{sp}\left(:,k\right)$ is the k-th column of $S_{sp}$.* Let $m=K,r=K,n=n_{c},Y_{sp}={\hat{V}}_{2}^{\prime },Z_{sp}={\hat{V}}_{2}^{\prime }-V_{2}^{\prime },S_{sp}=V_{2}^{\prime }\left(I_{c},:\right),$ and $M_{sp}=\Pi_{c}^{\prime }$. By Condition (I2), $M_{sp}$ has an identity submatrix $I_{K}$. By Lemma A7, we have

$$\begin{array}{c} \epsilon_{c}=\max_{1\leq j\leq n_{c}}∥{\hat{V}}_{2}\left(j,:\right)-V_{2}{\left(j,:\right)∥}_{F}={∥{\hat{V}}_{2}\left(j,:\right)-V_{2}\left(j,:\right)∥}_{2\to \infty }\leq \varpi . \end{array}$$

By Theorem A1, there exists a permutation matrix $P_{c}$ such that

$$\begin{array}{c} \max_{1\leq k\leq K}{∥e_{k}^{\prime }\left({\hat{V}}_{2}\left({\hat{I}}_{c},:\right)-P_{c}^{\prime }V_{2}\left(I_{c},:\right)\right)∥}_{F}=O\left(\epsilon_{c}\kappa^{2}\left(V_{2}\left(I_{c},:\right)\right)\sqrt{K}\right)=O\left(\varpi \kappa^{2}\left(V_{2}\left(I_{c},:\right)\right)\right). \end{array}$$

Since $\kappa^{2}\left(V_{2}\left(I_{c},:\right)\right)=\kappa \left(V_{2}\left(I_{c},:\right)V_{2}^{\prime }\left(I_{c},:\right)\right)=\kappa \left(V\left(I_{c},:\right)V^{\prime }\left(I_{c},:\right)\right)=\kappa \left(\Pi_{c}^{\prime }\Pi_{c}\right)$, where the last equality holds by Lemma A4, we have

$$\begin{array}{c} \max_{1\leq k\leq K}{∥e_{k}^{\prime }\left({\hat{V}}_{2}\left({\hat{I}}_{c},:\right)-P_{c}^{\prime }V_{2}\left(I_{c},:\right)\right)∥}_{F}=O\left(\varpi \kappa \left(\Pi_{c}^{\prime }\Pi_{c}\right)\right). \end{array}$$

**Remark A1.** *For the ideal case, let $m=K,r=K,n=n_{c},Y_{sp}=V^{\prime },Z_{sp}=V^{\prime }-V^{\prime }\equiv 0,S_{sp}=V^{\prime }\left(I_{c},:\right),$ and $M_{sp}=\Pi_{c}^{\prime }$. Then, we have $\max_{1\leq j\leq n_{c}}{∥V\left(j,:\right)-V\left(j,:\right)∥}_{F}=0$. By Theorem A1, SP algorithm returns $I_{c}$ when the input is V assuming there are K column communities.*
Now, we consider row nodes. From Lemma 2, we see that $U_{*}\left(I_{r},:\right)$ satisfies Condition 1 in [26]. Meanwhile, since ${\left(U_{*}\left(I_{r},:\right)U_{*}^{\prime }\left(I_{r},:\right)\right)}^{-1}1>0$, we have ${\left(U_{*}\left(I_{r},:\right)U_{*}^{\prime }\left(I_{r},:\right)\right)}^{-1}1\geq \eta 1$, hence $U_{*}\left(I_{r},:\right)$ satisfies Condition 2 in [26]. Now, we give a lower bound for η to show that η is strictly positive. By the proof of Lemma A4, we have

$$\begin{array}{cc} {\left(U_{*}\left(I_{r},:\right)U_{*}^{\prime }\left(I_{r},:\right)\right)}^{-1} & =N_{U}^{-1}\left(I_{r},I_{r}\right)\Theta^{-1}\left(I_{r},I_{r}\right)\Pi_{r}^{\prime }\Theta_{r}^{2}\Pi_{r}\Theta_{r}^{-1}\left(I_{r},I_{r}\right)N_{U}^{-1}\left(I_{r},I_{r}\right)\\ \; & \geq \frac{\theta_{r,\min}^{2}}{\theta_{r,\max}^{2}N_{U,\max}^{2}}\Pi_{r}^{\prime }\Pi_{r}\geq \frac{\theta_{r,\min}^{4}}{\theta_{r,\max}^{4}K\lambda_{1}\left(\Pi_{r}^{\prime }\Pi_{r}\right)}\Pi_{r}^{\prime }\Pi_{r}, \end{array}$$

where we set $N_{U,\max}=\max_{1\leq i\leq n_{r}}N_{U}\left(i,i\right)$, and we have used the facts that $N_{U},\Theta_{r}$ are diagonal matrices, and $N_{U,\max}\leq \frac{\theta_{r,\max}\sqrt{K\lambda_{1}\left(\Pi_{r}^{\prime }\Pi_{r}\right)}}{\theta_{r,\min}}$ by Lemma A3. Then, we have

$$\begin{array}{cc} \; & \eta =\min_{1\leq k\leq K}\left({\left(U_{*}\left(I_{r},:\right)U_{*}^{\prime }\left(I_{r},:\right)\right)}^{-1}1\right)\left(k\right)\geq \frac{\theta_{r,\min}^{4}}{\theta_{r,\max}^{4}K\lambda_{1}\left(\Pi_{r}^{\prime }\Pi_{r}\right)}\min_{1\leq k\leq K}e_{k}^{\prime }\Pi_{r}^{\prime }\Pi_{r}1\\ \; & =\frac{\theta_{r,\min}^{4}}{\theta_{r,\max}^{4}K\lambda_{1}\left(\Pi_{r}^{\prime }\Pi_{r}\right)}\min_{1\leq k\leq K}e_{k}^{\prime }\Pi_{r}^{\prime }1=\frac{\theta_{r,\min}^{4}\pi_{r,\min}}{\theta_{r,\max}^{4}K\lambda_{1}\left(\Pi_{r}^{\prime }\Pi_{r}\right)}, \end{array}$$

i.e., η is strictly positive. Since $U_{*,2}\left(I_{r},:\right)U_{*,2}^{\prime }\left(I_{r},:\right)\equiv U_{*}\left(I_{r},:\right)U_{*}^{\prime }\left(I_{r},:\right)$, we have $U_{*,2}\left(I_{r},:\right)$ also satisfies Conditions 1 and 2 in [26]. The above analysis shows that we can directly apply Lemma F.1 of [26] since the ideal DiMSC algorithm satisfies Conditions 1 and 2 in [26], therefore there exists a permutation matrix $P_{r}\in R^{K\times K}$ such that

$$\begin{array}{c} \max_{1\leq k\leq K}{∥e_{k}^{\prime }\left({\hat{U}}_{*,2}\left({\hat{I}}_{r},:\right)-P_{r}^{\prime }U_{*,2}\left(I_{r},:\right)\right)∥}_{F}=O\left(\frac{\sqrt{K}\zeta \epsilon_{r}}{\lambda_{K}^{1.5}\left(U_{*,2}\left(I_{r},:\right)\right)U_{*,2}^{\prime }\left(I_{r},:\right)}\right), \end{array}$$

where $\zeta \leq \frac{4K}{\eta \lambda_{K}^{1.5}\left(U_{*,2}\left(I_{r},:\right)U_{*,2}^{\prime }\left(I_{r},:\right)\right)}=O\left(\frac{K}{\eta \lambda_{K}^{1.5}\left(U_{*}\left(I_{r},:\right)U_{*}^{\prime }\left(I_{r},:\right)\right)}\right)$, and $\epsilon_{r}=\max_{1\leq i\leq n_{r}}∥{\hat{U}}_{*,2}\left(i,:\right)-U_{*,2}\left(i,:\right)∥$. Next, we bound $\epsilon_{r}$ as below

$$\begin{array}{cc} & ∥{\hat{U}}_{*,2}\left(i,:\right)-U_{*,2}{\left(i,:\right)∥}_{F}={∥\frac{{\hat{U}}_{2}\left(i,:\right)∥U_{2}{\left(i,:\right)∥}_{F}-U_{2}\left(i,:\right){∥{\hat{U}}_{2}\left(i,:\right)∥}_{F}}{∥{\hat{U}}_{2}{\left(i,:\right)∥}_{F}{∥U_{2}\left(i,:\right)∥}_{F}}∥}_{F}\leq \frac{2∥{\hat{U}}_{2}\left(i,:\right)-U_{2}{\left(i,:\right)∥}_{F}}{∥U_{2}{\left(i,:\right)∥}_{F}}\\ \; & \leq \frac{2∥{\hat{U}}_{2}-U_{2}{∥}_{2\to \infty }}{∥U_{2}{\left(i,:\right)∥}_{F}}\leq \frac{2\varpi }{∥U_{2}{\left(i,:\right)∥}_{F}}=\frac{2\varpi }{∥\left(UU^{\prime }\right)\left(i,:\right){∥}_{F}}=\frac{2\varpi }{∥U\left(i,:\right)U^{\prime }{∥}_{F}}=\frac{2\varpi }{{∥U\left(i,:\right)∥}_{F}}\\ \; & \leq 2\varpi \frac{\theta_{r,\max}\sqrt{K\lambda_{1}\left(\Pi_{r}^{\prime }\Pi_{r}\right)}}{\theta_{r,\min}}, \end{array}$$

where the last inequality holds by Lemma A3. Then, we have $\epsilon_{r}=O\left(\varpi \frac{\theta_{r,\max}\sqrt{K\lambda_{1}\left(\Pi_{r}^{\prime }\Pi_{r}\right)}}{\theta_{r,\min}}\right)$. Finally, by Lemma A4, we have

$$\begin{array}{c} \max_{1\leq k\leq K}{∥e_{k}^{\prime }\left({\hat{U}}_{*,2}\left({\hat{I}}_{r},:\right)-P_{r}^{\prime }U_{*,2}\left(I_{r},:\right)\right)∥}_{F}=O\left(\frac{K^{3}\theta_{r,\max}^{11}\varpi \kappa^{3}\left(\Pi_{r}^{\prime }\Pi_{r}\right)\lambda_{1}^{1.5}\left(\Pi_{r}^{\prime }\Pi_{r}\right)}{\theta_{r,\min}^{11}\pi_{r,\min}}\right). \end{array}$$

**Remark A2.** *For the ideal case, when setting $U_{*}$ as the input of the SVM-cone algorithm assuming there are K row communities, since $∥U_{*}-U_{*}{∥}_{2\to \infty }=0$, Lemma F.1; [26] guarantees that SVM-cone algorithm returns $I_{r}$ exactly. Meanwhile, another view to see that the SVM-cone algorithm exactly obtains $I_{r}$ when the input is $U_{*}$ (also $U_{2,*}$) is given in Appendix F, which focuses on following the three steps of SVM-cone algorithm to show that it returns $I_{r}$ with input $U_{*}$ (also $U_{*,2}$), instead of simply applying Lemma F.1. [26].*
   □

**Lemma A9.** 
*Under $DiDCMM_{n_{r},n_{c}}\left(K,P,\Pi_{r},\Pi_{c},\Theta_{r}\right)$, when conditions of Lemma A7 hold, with probability at least $1-o({\left(n_{r}+n_{c}\right)}^{-3})$, we have*

$$\begin{array}{cc} \; & \max_{1\leq i\leq n_{r}}{∥e_{i}^{\prime }\left({\hat{Z}}_{r}-Z_{r}P_{r}\right)∥}_{F}=O\left(\frac{K^{5}\theta_{r,\max}^{15}\varpi \kappa^{4.5}\left(\Pi_{r}^{\prime }\Pi_{r}\right)\kappa \left(\Pi_{c}\right)\lambda_{1}^{1.5}\left(\Pi_{r}^{\prime }\Pi_{r}\right)}{\theta_{r,\min}^{14}\pi_{r,\min}}\right),\\ \; & \max_{1\leq j\leq n_{c}}{∥e_{j}^{\prime }\left({\hat{Z}}_{c}-Z_{c}P_{c}\right)∥}_{F}=O\left(\varpi \kappa \left(\Pi_{c}^{\prime }\Pi_{c}\right)\sqrt{K\lambda_{1}\left(\Pi_{c}^{\prime }\Pi_{c}\right)}\right). \end{array}$$

**Proof.** First, we consider column nodes. Recall that $V\left(I_{c},:\right)=B_{c}$. For convenience, set $\hat{V}\left({\hat{I}}_{c},:\right)={\hat{B}}_{c},V_{2}\left(I_{c},:\right)=B_{2c},{\hat{V}}_{2}\left({\hat{I}}_{c},:\right)={\hat{B}}_{2c}$. We bound $∥e_{j}^{\prime }\left({\hat{Z}}_{c}-Z_{c}P_{c}\right){∥}_{F}$ when the input is $\hat{V}$ in the SP algorithm. Recall that $Z_{c}=\max\left(VV^{\prime }\left(I_{c},:\right){\left(V\left(I_{c},:\right)V^{\prime }\left(I_{c},:\right)\right)}^{-1},0\right)\equiv \Pi_{c}$, for $1\leq j\leq n_{c}$, we have

$$\begin{array}{cc} & ∥e_{j}^{\prime }\left({\hat{Z}}_{c}-Z_{c}P_{c}\right){∥}_{F}={∥e_{j}^{\prime }\left(\max\left(0,\hat{V}{\hat{B}}_{c}^{\prime }{\left({\hat{B}}_{c}{\hat{B}}_{c}^{\prime }\right)}^{-1}\right)-VB_{c}^{\prime }{\left(B_{c}B_{c}^{\prime }\right)}^{-1}P_{c}\right)∥}_{F}\\ \; & \leq ∥e_{j}^{\prime }\left(\hat{V}{\hat{B}}_{c}^{\prime }{\left({\hat{B}}_{c}{\hat{B}}_{c}^{\prime }\right)}^{-1}-VB_{c}^{\prime }{\left(B_{c}B_{c}^{\prime }\right)}^{-1}P_{c}\right){∥}_{F}\\ & =∥e_{j}^{\prime }\left(\hat{V}-V\left(V^{\prime }\hat{V}\right)\right){\hat{B}}_{c}^{\prime }{\left({\hat{B}}_{c}{\hat{B}}_{c}^{\prime }\right)}^{-1}+e_{j}^{\prime }\left(V\left(V^{\prime }\hat{V}\right){\hat{B}}_{c}^{\prime }{\left({\hat{B}}_{c}{\hat{B}}_{c}^{\prime }\right)}^{-1}-V\left(V^{\prime }\hat{V}\right){\left(P_{c}^{\prime }\left(B_{c}B_{c}^{\prime }\right){\left(B_{c}^{\prime }\right)}^{-1}\left(V^{\prime }\hat{V}\right)\right)}^{-1}\right){∥}_{F}\\ & \leq ∥e_{j}^{\prime }\left(\hat{V}-V\left(V^{\prime }\hat{V}\right)\right){\hat{B}}_{c}^{\prime }{\left({\hat{B}}_{c}{\hat{B}}_{c}^{\prime }\right)}^{-1}{∥}_{F}+{∥e_{j}^{\prime }V\left(V^{\prime }\hat{V}\right)\left({\hat{B}}_{c}^{\prime }{\left({\hat{B}}_{c}{\hat{B}}_{c}^{\prime }\right)}^{-1}-{\left(P_{c}^{\prime }\left(B_{c}B_{c}^{\prime }\right){\left(B_{c}^{\prime }\right)}^{-1}\left(V^{\prime }\hat{V}\right)\right)}^{-1}\right)∥}_{F}\\ \; & \leq ∥e_{j}^{\prime }\left(\hat{V}-V\left(V^{\prime }\hat{V}\right)\right){∥}_{F}∥{\hat{B}}_{c}^{-1}{∥}_{F}+{∥e_{j}^{\prime }V\left(V^{\prime }\hat{V}\right)\left({\hat{B}}_{c}^{\prime }{\left({\hat{B}}_{c}{\hat{B}}_{c}^{\prime }\right)}^{-1}-{\left(P_{c}^{\prime }\left(B_{c}B_{c}^{\prime }\right){\left(B_{c}^{\prime }\right)}^{-1}\left(V^{\prime }\hat{V}\right)\right)}^{-1}\right)∥}_{F}\\ & \leq \sqrt{K}∥e_{j}^{\prime }\left(\hat{V}-V\left(V^{\prime }\hat{V}\right)\right){∥}_{F}/\sqrt{\lambda_{K}\left({\hat{B}}_{c}{\hat{B}}_{c}^{\prime }\right)}+{∥e_{j}^{\prime }V\left(V^{\prime }\hat{V}\right)\left({\hat{B}}_{c}^{-1}-{\left(P_{c}^{\prime }B_{c}\left(V^{\prime }\hat{V}\right)\right)}^{-1}\right)∥}_{F}\\ \; & =\sqrt{K}∥e_{j}^{\prime }\left(\hat{V}{\hat{V}}^{\prime }-VV^{\prime }\right)\hat{V}{∥}_{F}O\left(\sqrt{\lambda_{1}\left(\Pi_{c}^{\prime }\Pi_{c}\right)}\right)+{∥e_{j}^{\prime }V\left(V^{\prime }\hat{V}\right)\left({\hat{B}}_{c}^{-1}-{\left(P_{c}^{\prime }B_{c}\left(V^{\prime }\hat{V}\right)\right)}^{-1}\right)∥}_{F}\\ \; & \leq \sqrt{K}∥e_{j}^{\prime }\left(\hat{V}{\hat{V}}^{\prime }-VV^{\prime }\right){∥}_{F}O\left(\sqrt{\lambda_{1}\left(\Pi_{c}^{\prime }\Pi_{c}\right)}\right)+{∥e_{j}^{\prime }V\left(V^{\prime }\hat{V}\right)\left({\hat{B}}_{c}^{-1}-{\left(P_{c}^{\prime }B_{c}\left(V^{\prime }\hat{V}\right)\right)}^{-1}\right)∥}_{F}\\ \; & \leq \sqrt{K}\varpi O\left(\sqrt{\lambda_{1}\left(\Pi_{c}^{\prime }\Pi_{c}\right)}\right)+{∥e_{j}^{\prime }V\left(V^{\prime }\hat{V}\right)\left({\hat{B}}_{c}^{-1}-{\left(P_{c}^{\prime }B_{c}\left(V^{\prime }\hat{V}\right)\right)}^{-1}\right)∥}_{F}\\ & =O\left(\varpi \sqrt{K\lambda_{1}\left(\Pi_{c}^{\prime }\Pi_{c}\right)}\right)+{∥e_{j}^{\prime }V\left(V^{\prime }\hat{V}\right)\left({\hat{B}}_{c}^{-1}-{\left(P_{c}^{\prime }B_{c}\left(V^{\prime }\hat{V}\right)\right)}^{-1}\right)∥}_{F}, \end{array}$$

where we have used similar idea in the proof of Lemma VII.3 in [27] such that apply $O(\frac{1}{\lambda_{K}\left(B_{c}B_{c}^{\prime }\right)})$ to estimate $\frac{1}{\lambda_{K}\left({\hat{B}}_{c}{\hat{B}}_{c}^{\prime }\right)}$, then by Lemma A4, we have $\frac{1}{\lambda_{K}\left({\hat{B}}_{c}{\hat{B}}_{c}^{\prime }\right)}=O\left(\lambda_{1}\left(\Pi_{c}^{\prime }\Pi_{c}\right)\right)$. Now, we aim to bound $∥e_{j}^{\prime }V\left(V^{\prime }\hat{V}\right)\left({\hat{B}}_{c}^{-1}-{\left(P_{c}^{\prime }B_{c}\left(V^{\prime }\hat{V}\right)\right)}^{-1}\right){∥}_{F}$. For convenience, set $T_{c}=V^{\prime }\hat{V},S_{c}=P_{c}^{\prime }B_{c}T_{c}$. We have

(A2) $$\begin{array}{c} ∥e_{j}^{\prime }V\left(V^{\prime }\hat{V}\right)\left({\hat{B}}_{c}^{-1}-{\left(P_{c}^{\prime }B_{c}\left(V^{\prime }\hat{V}\right)\right)}^{-1}\right){∥}_{F}={∥e_{j}^{\prime }VT_{c}S_{c}^{-1}\left(S_{c}-{\hat{B}}_{c}\right){\hat{B}}_{c}^{-1}∥}_{F}\\ \leq ∥e_{j}^{\prime }VT_{c}S_{c}^{-1}\left(S_{c}-{\hat{B}}_{c}\right){∥}_{F}∥{\hat{B}}_{c}^{-1}{∥}_{F}\leq {∥e_{j}^{\prime }VT_{c}S_{c}^{-1}\left(S_{c}-{\hat{B}}_{c}\right)∥}_{F}\frac{\sqrt{K}}{|\lambda_{K}\left({\hat{B}}_{c}\right)|}\\ =∥e_{j}^{\prime }VT_{c}S_{c}^{-1}\left(S_{c}-{\hat{B}}_{c}\right){∥}_{F}\frac{\sqrt{K}}{\sqrt{\lambda_{K}\left({\hat{B}}_{c}{\hat{B}}_{c}^{\prime }\right)}}\leq {∥e_{j}^{\prime }VT_{c}S_{c}^{-1}\left(S_{c}-{\hat{B}}_{c}\right)∥}_{F}O\left(\sqrt{K\lambda_{1}\left(\Pi_{c}^{\prime }\Pi_{c}\right)}\right)\\ =∥e_{j}^{\prime }VT_{c}T_{c}^{-1}B_{c}^{\prime }{\left(B_{c}B_{c}^{\prime }\right)}^{-1}P_{c}\left(S_{c}-{\hat{B}}_{c}\right){∥}_{F}O\left(\sqrt{K\lambda_{1}\left(\Pi_{c}^{\prime }\Pi_{c}\right)}\right)\\ =∥e_{j}^{\prime }VB_{c}^{\prime }{\left(B_{c}B_{c}^{\prime }\right)}^{-1}P_{c}\left(S_{c}-{\hat{B}}_{c}\right){∥}_{F}O\left(\sqrt{K\lambda_{1}\left(\Pi_{c}^{\prime }\Pi_{c}\right)}\right)\\ =∥e_{j}^{\prime }Z_{c}P_{c}\left(S_{c}-{\hat{B}}_{c}\right){∥}_{F}O\left(\sqrt{K\lambda_{1}\left(\Pi_{c}^{\prime }\Pi_{c}\right)}\right)\overset{\mathrm{By}\;Z_{c}=\Pi_{c}}{\leq }\max_{1\leq k\leq K}{∥e_{k}^{\prime }\left(S_{c}-{\hat{B}}_{c}\right)∥}_{F}O\left(\sqrt{K\lambda_{1}\left(\Pi_{c}^{\prime }\Pi_{c}\right)}\right)\\ =\max_{1\leq k\leq K}{∥e_{k}^{\prime }\left({\hat{B}}_{c}-P_{c}^{\prime }B_{c}V^{\prime }\hat{V}\right)∥}_{F}O\left(\sqrt{K\lambda_{1}\left(\Pi_{c}^{\prime }\Pi_{c}\right)}\right)\\ =\max_{1\leq k\leq K}{∥e_{k}^{\prime }\left({\hat{B}}_{c}{\hat{V}}^{\prime }-P_{c}^{\prime }B_{c}V^{\prime }\right)\hat{V}∥}_{F}O\left(\sqrt{K\lambda_{1}\left(\Pi_{c}^{\prime }\Pi_{c}\right)}\right)\\ \leq \max_{1\leq k\leq K}{∥e_{k}^{\prime }\left({\hat{B}}_{c}{\hat{V}}^{\prime }-P_{c}^{\prime }B_{c}V^{\prime }\right)∥}_{F}O\left(\sqrt{K\lambda_{1}\left(\Pi_{c}^{\prime }\Pi_{c}\right)}\right)\\ =\max_{1\leq k\leq K}{∥e_{k}^{\prime }\left({\hat{B}}_{2c}-P_{c}^{\prime }B_{2c}\right)∥}_{F}O\left(\sqrt{K\lambda_{1}\left(\Pi_{c}^{\prime }\Pi_{c}\right)}\right)\\ =O(\varpi \kappa \left(\Pi_{c}^{\prime }\Pi_{c}\right)\sqrt{K\lambda_{1}\left(\Pi_{c}^{\prime }\Pi_{c}\right)}). \end{array}$$

**Remark A3.** *Equation (A2) supports our statement that building the theoretical framework of DiMSC benefits a lot by introducing the DiMSC-equivalence algorithm since $∥{\hat{B}}_{2c}-P_{c}^{\prime }B_{2c}{∥}_{2\to \infty }$ is obtained from DiMSC-equivalence (i.e., inputing ${\hat{V}}_{2}$ in the SP algorithm obtains $∥{\hat{B}}_{2c}-P_{c}^{\prime }B_{2c}{∥}_{2\to \infty }$).*

Then, we have

$$\begin{array}{cc} \; & ∥e_{j}^{\prime }\left({\hat{Z}}_{c}-Z_{c}P_{c}\right){∥}_{F}\leq O\left(\varpi \sqrt{K\lambda_{1}\left(\Pi_{c}^{\prime }\Pi_{c}\right)}\right)+{∥e_{j}^{\prime }V\left(V^{\prime }\hat{V}\right)\left({\hat{B}}_{c}^{-1}-{\left(P_{c}^{\prime }B_{c}\left(V^{\prime }\hat{V}\right)\right)}^{-1}\right)∥}_{F}\\ \; & \leq O\left(\varpi \sqrt{K\lambda_{1}\left(\Pi_{c}^{\prime }\Pi_{c}\right)}\right)+O\left(\varpi \kappa \left(\Pi_{c}^{\prime }\Pi_{c}\right)\sqrt{K\lambda_{1}\left(\Pi_{c}^{\prime }\Pi_{c}\right)}\right)=O\left(\varpi \kappa \left(\Pi_{c}^{\prime }\Pi_{c}\right)\sqrt{K\lambda_{1}\left(\Pi_{c}^{\prime }\Pi_{c}\right)}\right). \end{array}$$

Next, we consider row nodes. For $1\leq i\leq n_{r}$, since $Z_{r}=Y_{*}J_{*},{\hat{Z}}_{r}={\hat{Y}}_{*}{\hat{J}}_{*}$, we have

$$\begin{array}{cc} \; & ∥e_{i}^{\prime }\left({\hat{Z}}_{r}-Z_{r}P_{r}\right){∥}_{F}=∥e_{i}^{\prime }\left(\max\left(0,{\hat{Y}}_{*}{\hat{J}}_{*}\right)-Y_{*}J_{*}P_{r}\right){∥}_{F}\leq {∥e_{i}^{\prime }\left({\hat{Y}}_{*}{\hat{J}}_{*}-Y_{*}J_{*}P_{r}\right)∥}_{F}\\ \; & =∥e_{i}^{\prime }\left({\hat{Y}}_{*}-Y_{*}P_{r}\right){\hat{J}}_{*}+e_{i}^{\prime }Y_{*}P_{r}\left({\hat{J}}_{*}-P_{r}^{\prime }J_{*}P_{r}\right){∥}_{F}\\ \; & \leq ∥e_{i}^{\prime }\left({\hat{Y}}_{*}-Y_{*}P_{r}\right){∥}_{F}∥{\hat{J}}_{*}{∥}_{F}+∥e_{i}^{\prime }Y_{*}P_{r}{∥}_{F}{∥{\hat{J}}_{*}-P_{r}^{\prime }J_{*}P_{r}∥}_{F}\\ \; & =∥e_{i}^{\prime }\left({\hat{Y}}_{*}-Y_{*}P_{r}\right){∥}_{F}∥{\hat{J}}_{*}{∥}_{F}+∥e_{i}^{\prime }Y_{*}{∥}_{F}{∥{\hat{J}}_{*}-P_{r}^{\prime }J_{*}P_{r}∥}_{F}. \end{array}$$

Next, we consider row nodes. For $1\leq i\leq n_{r}$, since $Z_{r}=Y_{*}J_{*},{\hat{Z}}_{r}={\hat{Y}}_{*}{\hat{J}}_{*}$, we have

$$\begin{array}{cc} \; & ∥e_{i}^{\prime }\left({\hat{Z}}_{r}-Z_{r}P_{r}\right){∥}_{F}=∥e_{i}^{\prime }\left(\max\left(0,{\hat{Y}}_{*}{\hat{J}}_{*}\right)-Y_{*}J_{*}P_{r}\right){∥}_{F}\leq {∥e_{i}^{\prime }\left({\hat{Y}}_{*}{\hat{J}}_{*}-Y_{*}J_{*}P_{r}\right)∥}_{F}\\ \; & =∥e_{i}^{\prime }\left({\hat{Y}}_{*}-Y_{*}P_{r}\right){\hat{J}}_{*}+e_{i}^{\prime }Y_{*}P_{r}\left({\hat{J}}_{*}-P_{r}^{\prime }J_{*}P_{r}\right){∥}_{F}\\ \; & \leq ∥e_{i}^{\prime }\left({\hat{Y}}_{*}-Y_{*}P_{r}\right){∥}_{F}∥{\hat{J}}_{*}{∥}_{F}+∥e_{i}^{\prime }Y_{*}P_{r}{∥}_{F}{∥{\hat{J}}_{*}-P_{r}^{\prime }J_{*}P_{r}∥}_{F}\\ \; & =∥e_{i}^{\prime }\left({\hat{Y}}_{*}-Y_{*}P_{r}\right){∥}_{F}∥{\hat{J}}_{*}{∥}_{F}+∥e_{i}^{\prime }Y_{*}{∥}_{F}{∥{\hat{J}}_{*}-P_{r}^{\prime }J_{*}P_{r}∥}_{F}. \end{array}$$

Therefore, the bound of $∥e_{i}^{\prime }\left({\hat{Z}}_{r}-Z_{r}P_{r}\right){∥}_{F}$ can be obtained as long as we bound $∥e_{i}^{\prime }\left({\hat{Y}}_{*}-Y_{*}P_{r}\right){∥}_{F},∥{\hat{J}}_{*}{∥}_{F},{∥e_{i}^{\prime }Y_{*}∥}_{F}$ and $∥{\hat{J}}_{*}-P_{r}^{\prime }J_{*}P_{r}{∥}_{F}$. We bound the four terms as below:

- We bound $∥e_{i}^{\prime }\left({\hat{Y}}_{*}-Y_{*}P_{r}\right){∥}_{F}$ first. Similar as bounding $∥e_{j}^{\prime }\left({\hat{Z}}_{c}-Z_{c}P_{c}\right)∥$, we set $U_{*}\left(I_{r},:\right)=B_{R},{\hat{U}}_{*}\left({\hat{I}}_{r},:\right)={\hat{B}}_{R},U_{*,2}\left(I_{r},:\right)=B_{2R},{\hat{U}}_{*,2}\left({\hat{I}}_{r},:\right)={\hat{B}}_{2R}$ for convenience. We bound $∥e_{i}^{\prime }\left({\hat{Y}}_{*}-Y_{*}P_{r}\right){∥}_{F}$ when the input is ${\hat{U}}_{*}$ in the SVM-cone algorithm. For $1\leq i\leq n_{r}$, we have
  $$\begin{array}{cc} & ∥e_{i}^{\prime }\left({\hat{Y}}_{*}-Y_{*}P_{r}\right){∥}_{F}={∥e_{i}^{\prime }\left(\hat{U}{\hat{B}}_{R}^{\prime }{\left({\hat{B}}_{R}{\hat{B}}_{R}^{\prime }\right)}^{-1}-UB_{R}^{\prime }{\left(B_{R}B_{R}^{\prime }\right)}^{-1}P_{r}\right)∥}_{F}\\ & =∥e_{i}^{\prime }\left(\hat{U}-U\left(U^{\prime }\hat{U}\right)\right){\hat{B}}_{R}^{\prime }{\left({\hat{B}}_{R}{\hat{B}}_{R}^{\prime }\right)}^{-1}+e_{i}^{\prime }(U\left(U^{\prime }\hat{U}\right){\hat{B}}_{R}^{\prime }{\left({\hat{B}}_{R}{\hat{B}}_{R}^{\prime }\right)}^{-1}\\ \; & \;\;\;-U\left(U^{\prime }\hat{U}\right){\left(P_{r}^{\prime }\left(B_{R}B_{R}^{\prime }\right){\left(B_{R}^{\prime }\right)}^{-1}\left(U^{\prime }\hat{U}\right)\right)}^{-1}{)∥}_{F}\\ & \leq ∥e_{i}^{\prime }\left(\hat{U}-U\left(U^{\prime }\hat{U}\right)\right){\hat{B}}_{R}^{\prime }{\left({\hat{B}}_{R}{\hat{B}}_{R}^{\prime }\right)}^{-1}{∥}_{F}+∥e_{i}^{\prime }U\left(U^{\prime }\hat{U}\right)({\hat{B}}_{R}^{\prime }{\left({\hat{B}}_{R}{\hat{B}}_{R}^{\prime }\right)}^{-1}\\ \; & \;\;\;-{\left(P_{r}^{\prime }\left(B_{R}B_{R}^{\prime }\right){\left(B_{R}^{\prime }\right)}^{-1}\left(U^{\prime }\hat{U}\right)\right)}^{-1}{)∥}_{F}\\ \; & \leq ∥e_{i}^{\prime }\left(\hat{U}-U\left(U^{\prime }\hat{U}\right)\right){∥}_{F}∥{\hat{B}}_{R}^{-1}{∥}_{F}+{∥e_{i}^{\prime }U\left(U^{\prime }\hat{U}\right)\left({\hat{B}}_{R}^{\prime }{\left({\hat{B}}_{R}{\hat{B}}_{R}^{\prime }\right)}^{-1}-{\left(P_{r}^{\prime }\left(B_{R}B_{R}^{\prime }\right){\left(B_{R}^{\prime }\right)}^{-1}\left(U^{\prime }\hat{U}\right)\right)}^{-1}\right)∥}_{F}\\ & \leq \sqrt{K}∥e_{i}^{\prime }\left(\hat{U}-U\left(U^{\prime }\hat{U}\right)\right){∥}_{F}/\sqrt{\lambda_{K}\left({\hat{B}}_{R}{\hat{B}}_{R}^{\prime }\right)}+{∥e_{i}^{\prime }U\left(U^{\prime }\hat{U}\right)\left({\hat{B}}_{R}^{-1}-{\left(P_{r}^{\prime }B_{R}\left(U^{\prime }\hat{U}\right)\right)}^{-1}\right)∥}_{F}\\ \; & \overset{\left(i\right)}{=}\sqrt{K}∥e_{i}^{\prime }\left(\hat{U}{\hat{U}}^{\prime }-UU^{\prime }\right)\hat{U}{∥}_{F}O\left(\frac{\theta_{r,\max}\sqrt{\kappa (\Pi_{r}^{\prime }\Pi_{r})}}{\theta_{r,\min}}\right)+{∥e_{i}^{\prime }U\left(U^{\prime }\hat{U}\right)\left({\hat{B}}_{R}^{-1}-{\left(P_{r}^{\prime }B_{R}\left(U^{\prime }\hat{U}\right)\right)}^{-1}\right)∥}_{F}\\ \; & \leq \sqrt{K}∥e_{i}^{\prime }\left(\hat{U}{\hat{U}}^{\prime }-UU^{\prime }\right){∥}_{F}O\left(\frac{\theta_{r,\max}\sqrt{\kappa (\Pi_{r}^{\prime }\Pi_{r})}}{\theta_{r,\min}}\right)+{∥e_{i}^{\prime }U\left(U^{\prime }\hat{U}\right)\left({\hat{B}}_{R}^{-1}-{\left(P_{r}^{\prime }B_{R}\left(U^{\prime }\hat{U}\right)\right)}^{-1}\right)∥}_{F}\\ \; & \leq \sqrt{K}\varpi O\left(\frac{\theta_{r,\max}\sqrt{\kappa (\Pi_{r}^{\prime }\Pi_{r})}}{\theta_{r,\min}}\right)+{∥e_{i}^{\prime }U\left(U^{\prime }\hat{U}\right)\left({\hat{B}}_{R}^{-1}-{\left(P_{r}^{\prime }B_{R}\left(U^{\prime }\hat{U}\right)\right)}^{-1}\right)∥}_{F}\\ & =O\left(\varpi \frac{\theta_{r,\max}\sqrt{K\kappa (\Pi_{r}^{\prime }\Pi_{r})}}{\theta_{r,\min}}\right)+{∥e_{i}^{\prime }U\left(U^{\prime }\hat{U}\right)\left({\hat{B}}_{R}^{-1}-{\left(P_{r}^{\prime }B_{R}\left(U^{\prime }\hat{U}\right)\right)}^{-1}\right)∥}_{F}, \end{array}$$
  where we have used similar idea in the proof of Lemma VII.3 in [27] such that we apply $O(\frac{1}{\lambda_{K}\left(B_{R}B_{R}^{\prime }\right)})$ to estimate $\frac{1}{\lambda_{K}\left({\hat{B}}_{R}{\hat{B}}_{R}^{\prime }\right)}$, hence (i) holds by Lemma A4.

Now, we aim to bound $∥e_{i}^{\prime }U\left(U^{\prime }\hat{U}\right)\left({\hat{B}}_{R}^{-1}-{\left(P_{r}^{\prime }B_{R}\left(U^{\prime }\hat{U}\right)\right)}^{-1}\right){∥}_{F}$. For convenience, set $T_{r}=U^{\prime }\hat{U},S_{r}=P_{r}^{\prime }B_{R}T_{r}$. We have

(A3) $$\begin{array}{c} ∥e_{i}^{\prime }U\left(U^{\prime }\hat{U}\right)\left({\hat{B}}_{R}^{-1}-{\left(P_{r}^{\prime }B_{R}\left(U^{\prime }\hat{U}\right)\right)}^{-1}\right){∥}_{F}={∥e_{i}^{\prime }UT_{r}S_{r}^{-1}\left(S_{r}-{\hat{B}}_{R}\right){\hat{B}}_{R}^{-1}∥}_{F}\\ \leq ∥e_{i}^{\prime }UT_{r}S_{r}^{-1}\left(S_{r}-{\hat{B}}_{R}\right){∥}_{F}∥{\hat{B}}_{R}^{-1}{∥}_{F}\leq {∥e_{i}^{\prime }UT_{r}S_{r}^{-1}\left(S_{r}-{\hat{B}}_{R}\right)∥}_{F}\frac{\sqrt{K}}{|\lambda_{K}\left({\hat{B}}_{R}\right)|}\\ =∥e_{i}^{\prime }UT_{r}S_{r}^{-1}\left(S_{r}-{\hat{B}}_{R}\right){∥}_{F}\frac{\sqrt{K}}{\sqrt{\lambda_{K}\left({\hat{B}}_{R}{\hat{B}}_{R}^{\prime }\right)}}\leq {∥e_{i}^{\prime }UT_{r}S_{r}^{-1}\left(S_{r}-{\hat{B}}_{R}\right)∥}_{F}O\left(\frac{\theta_{r,\max}\sqrt{K\kappa (\Pi_{r}^{\prime }\Pi_{r})}}{\theta_{r,\min}}\right)\\ =∥e_{i}^{\prime }UT_{r}T_{r}^{-1}B_{R}^{\prime }{\left(B_{R}B_{R}^{\prime }\right)}^{-1}P_{r}\left(S_{r}-{\hat{B}}_{R}\right){∥}_{F}O\left(\frac{\theta_{r,\max}\sqrt{K\kappa (\Pi_{r}^{\prime }\Pi_{r})}}{\theta_{r,\min}}\right)\\ =∥e_{i}^{\prime }UB_{R}^{\prime }{\left(B_{R}B_{R}^{\prime }\right)}^{-1}P_{r}\left(S_{r}-{\hat{B}}_{R}\right){∥}_{F}O\left(\frac{\theta_{r,\max}\sqrt{K\kappa (\Pi_{r}^{\prime }\Pi_{r})}}{\theta_{r,\min}}\right)\\ =∥e_{i}^{\prime }Y_{*}P_{r}\left(S_{r}-{\hat{B}}_{R}\right){∥}_{F}O\left(\frac{\theta_{r,\max}\sqrt{K\lambda_{1}\left(\Pi_{r}^{\prime }\Pi_{r}\right)}}{\theta_{r,\min}}\right)\leq ∥e_{i}^{\prime }Y_{*}{∥}_{F}{∥S_{r}-{\hat{B}}_{R}∥}_{F}O\left(\frac{\theta_{r,\max}\sqrt{K\lambda_{1}\left(\Pi_{r}^{\prime }\Pi_{r}\right)}}{\theta_{r,\min}}\right)\\ \overset{\mathrm{By}\;\mathrm{Equation}(A4)}{\leq }\frac{\theta_{r,\max}^{2}\sqrt{K\lambda_{1}\left(\Pi_{r}^{\prime }\Pi_{r}\right)}}{\theta_{r,\min}^{2}\lambda_{K}\left(\Pi_{r}^{\prime }\Pi_{r}\right)}\max_{1\leq k\leq K}{∥e_{k}^{\prime }\left(S_{r}-{\hat{B}}_{R}\right)∥}_{F}O\left(\frac{\theta_{r,\max}K\sqrt{\kappa (\Pi_{r}^{\prime }\Pi_{r})}}{\theta_{r,\min}}\right)\\ =\max_{1\leq k\leq K}{∥e_{k}^{\prime }\left({\hat{B}}_{R}-P_{r}^{\prime }B_{R}U^{\prime }\hat{U}\right)∥}_{F}O\left(\frac{\theta_{r,\max}^{3}K^{1.5}\kappa \left(\Pi_{r}^{\prime }\Pi_{r}\right)}{\theta_{r,\min}^{3}\sqrt{\lambda_{K}\left(\Pi_{r}^{\prime }\Pi_{r}\right)}}\right)\\ =\max_{1\leq k\leq K}{∥e_{k}^{\prime }\left({\hat{B}}_{R}{\hat{U}}^{\prime }-P_{r}^{\prime }B_{R}U^{\prime }\right)\hat{U}∥}_{F}O\left(\frac{\theta_{r,\max}^{3}K^{1.5}\kappa \left(\Pi_{r}^{\prime }\Pi_{r}\right)}{\theta_{r,\min}^{3}\sqrt{\lambda_{K}\left(\Pi_{r}^{\prime }\Pi_{r}\right)}}\right)\\ \leq \max_{1\leq k\leq K}{∥e_{k}^{\prime }\left({\hat{B}}_{R}{\hat{U}}^{\prime }-P_{r}^{\prime }B_{R}U^{\prime }\right)∥}_{F}O\left(\frac{\theta_{r,\max}^{3}K^{1.5}\kappa \left(\Pi_{r}^{\prime }\Pi_{r}\right)}{\theta_{r,\min}^{3}\sqrt{\lambda_{K}\left(\Pi_{r}^{\prime }\Pi_{r}\right)}}\right)\\ =\max_{1\leq k\leq K}{∥e_{k}^{\prime }\left({\hat{B}}_{2R}-P_{r}^{\prime }B_{2R}\right)∥}_{F}O\left(\frac{\theta_{r,\max}^{3}K^{1.5}\kappa \left(\Pi_{r}^{\prime }\Pi_{r}\right)}{\theta_{r,\min}^{3}\sqrt{\lambda_{K}\left(\Pi_{r}^{\prime }\Pi_{r}\right)}}\right)\\ \overset{\mathrm{By}\;\mathrm{Lemma}A8\;}{=}O\left(\frac{K^{4.5}\theta_{r,\max}^{14}\varpi \kappa^{4.5}\left(\Pi_{r}^{\prime }\Pi_{r}\right)\lambda_{1}\left(\Pi_{r}^{\prime }\Pi_{r}\right)}{\theta_{r,\min}^{14}\pi_{r,\min}}\right). \end{array}$$

**Remark A4.** *Similar as Equation (A2), Equation (A3) supports our statement that building the theoretical framework of DiMSC benefits a lot by introducing the DiMSC-equivalence algorithm since $∥{\hat{B}}_{2R}-P_{r}^{\prime }B_{2R}{∥}_{2\to \infty }$ is obtained from DiMSC-equivalence (i.e., inputing ${\hat{U}}_{*,2}$ in the SVM-cone algorithm obtains $∥{\hat{B}}_{2R}-P_{r}^{\prime }B_{2R}{∥}_{2\to \infty }$).*

Then, we have

$$\begin{array}{cc} ∥e_{i}^{\prime }\left({\hat{Y}}_{*}-Y_{*}P_{r}\right){∥}_{F} & \leq O\left(\varpi \frac{\theta_{r,\max}\sqrt{K\kappa (\Pi_{r}^{\prime }\Pi_{r})}}{\theta_{r,\min}}\right)+∥e_{i}^{\prime }U\left(U^{\prime }\hat{U}\right){\left({\hat{B}}_{R}^{-1}-\left(P_{r}^{\prime }B_{R}U^{\prime }\hat{U}\right)\right)}^{-1}{)∥}_{F}\\ \; & \leq O\left(\varpi \frac{\theta_{r,\max}\sqrt{K\kappa (\Pi_{r}^{\prime }\Pi_{r})}}{\theta_{r,\min}}\right)+O\left(\frac{K^{4.5}\theta_{r,\max}^{14}\varpi \kappa^{4.5}\left(\Pi_{r}^{\prime }\Pi_{r}\right)\lambda_{1}\left(\Pi_{r}^{\prime }\Pi_{r}\right)}{\theta_{r,\min}^{14}\pi_{r,\min}}\right)\\ \; & =O(\frac{K^{4.5}\theta_{r,\max}^{14}\varpi \kappa^{4.5}\left(\Pi_{r}^{\prime }\Pi_{r}\right)\lambda_{1}\left(\Pi_{r}^{\prime }\Pi_{r}\right)}{\theta_{r,\min}^{14}\pi_{r,\min}}). \end{array}$$

- for $∥e_{i}^{\prime }Y_{*}{∥}_{F}$, since $Y_{*}=UU_{*}^{-1}\left(I_{r},:\right)$, by Lemmas (A3) and (A4), we have
  (A4) $$\begin{array}{c} ∥e_{i}^{\prime }Y_{*}{∥}_{F}\leq {∥U\left(i,:\right)∥}_{F}{∥U_{*}^{-1}\left(I_{r},:\right)∥}_{F}\leq \frac{\sqrt{K}{∥U\left(i,:\right)∥}_{F}}{\sqrt{\lambda_{K}\left(U_{*}\left(I_{r},:\right)U_{*}^{\prime }\left(I_{r},:\right)\right)}}\leq \frac{\theta_{r,\max}^{2}\sqrt{K\lambda_{1}\left(\Pi_{r}^{\prime }\Pi_{r}\right)}}{\theta_{r,\min}^{2}\lambda_{K}\left(\Pi_{r}^{\prime }\Pi_{r}\right)}. \end{array}$$
- for $∥{\hat{J}}_{*}{∥}_{F}$, recall that ${\hat{J}}_{*}=\mathrm{diag}\left({\hat{U}}_{*}\left({\hat{I}}_{r},:\right)\hat{\Lambda }{\hat{V}}^{\prime }\left({\hat{I}}_{c},:\right)\right)$, we have
  $$\begin{array}{cc} \; & ∥{\hat{J}}_{*}∥=\max_{1\leq k\leq K}{\hat{J}}_{*}\left(k,k\right)=\max_{1\leq k\leq K}e_{k}^{\prime }{\hat{U}}_{*}\left({\hat{I}}_{r},:\right)\hat{\Lambda }{\hat{V}}^{\prime }\left({\hat{I}}_{c},:\right)e_{k}\\ \; & =\max_{1\leq k\leq K}∥e_{k}^{\prime }{\hat{U}}_{*}\left({\hat{I}}_{r},:\right)\hat{\Lambda }{\hat{V}}^{\prime }\left({\hat{I}}_{c},:\right)e_{k}∥\\ \; & \leq \max_{1\leq k\leq K}∥e_{k}^{\prime }{\hat{U}}_{*}\left({\hat{I}}_{r},:\right)∥∥\hat{\Lambda }∥∥{\hat{V}}^{\prime }\left({\hat{I}}_{c},:\right)e_{k}∥\leq \max_{1\leq k\leq K}∥e_{k}^{\prime }{\hat{U}}_{*}\left({\hat{I}}_{r},:\right){∥}_{F}∥\hat{\Lambda }∥∥{\hat{V}}^{\prime }\left({\hat{I}}_{c},:\right)e_{k}∥\\ \; & =\max_{1\leq k\leq K}∥A∥∥{\hat{V}}^{\prime }\left({\hat{I}}_{c},:\right)e_{k}∥=\max_{1\leq k\leq K}∥A∥∥{\left(e_{k}^{\prime }\hat{V}\left({\hat{I}}_{c},:\right)\right)}^{\prime }∥=\max_{1\leq k\leq K}∥A∥∥e_{k}^{\prime }\hat{V}\left({\hat{I}}_{c},:\right)∥\\ \; & \leq \max_{1\leq k\leq K}∥A∥∥e_{k}^{\prime }\hat{V}\left({\hat{I}}_{c},:\right){∥}_{F}\leq ∥A∥∥\hat{V}{∥}_{2\to \infty }=∥A∥∥\hat{V}\mathrm{sgn}\left(H_{\hat{V}}\right){-V+V∥}_{2\to \infty }\\ \; & \leq ∥A∥(∥\hat{V}\mathrm{sgn}\left(H_{\hat{V}}\right)-V{∥}_{2\to \infty }+∥V{∥}_{2\to \infty }). \end{array}$$
  By Lemmas (A6) and (A5), $∥A∥=∥A-\Omega +\Omega ∥\leq ∥A-\Omega ∥+\sigma_{1}\left(\Omega \right)\leq ∥A-\Omega ∥+\theta_{r,\max}\sigma_{1}\left(P\right)\sigma_{1}\left(\Pi_{r}\right)\sigma_{1}\left(\Pi_{c}\right)=O\left(\theta_{r,\max}\sigma_{1}\left(\Pi_{r}\right)\sigma_{1}\left(\Pi_{c}\right)\right)$. By Lemma (A5) and Equation (A1),
  $∥\hat{V}\mathrm{sgn}\left(H_{\hat{V}}\right){-V∥}_{2\to \infty }\leq C\frac{\sqrt{\theta_{r,\max}K\log\left(n_{r}+n_{c}\right)}}{\theta_{r,\min}\sigma_{K}\left(P\right)\sigma_{K}\left(\Pi_{r}\right)\sigma_{K}\left(\Pi_{c}\right)}$. By Lemma A3, ${∥V∥}_{2\to \infty }\leq \sqrt{\frac{1}{\lambda_{K}\left(\Pi_{c}^{\prime }\Pi_{c}\right)}}$, which gives $∥\hat{V}\mathrm{sgn}\left(H_{\hat{V}}\right){-V∥}_{2\to \infty }+{∥V∥}_{2\to \infty }=O\left(\sqrt{\frac{1}{\lambda_{K}\left(\Pi_{c}^{\prime }\Pi_{c}\right)}}\right)$ (this can be seen as simply using ${∥V∥}_{2\to \infty }$ to estimate $∥\hat{V}{∥}_{2\to \infty }$ since $\sqrt{\frac{1}{\lambda_{K}\left(\Pi_{c}^{\prime }\Pi_{c}\right)}}$ is the same order as $\frac{\sqrt{\theta_{r,\max}K\log\left(n_{r}+n_{c}\right)}}{\theta_{r,\min}\sigma_{K}\left(P\right)\sigma_{K}\left(\Pi_{r}\right)\sigma_{K}\left(\Pi_{c}\right)}$). Then, we have $∥{\hat{J}}_{*}∥=O\left(\theta_{r,\max}\sigma_{1}\left(\Pi_{r}\right)\kappa \left(\Pi_{c}\right)\right)$, which gives that $∥{\hat{J}}_{*}{∥}_{F}=O\left(\theta_{r,\max}\sqrt{K}\sigma_{1}\left(\Pi_{r}\right)\kappa \left(\Pi_{c}\right)\right)$.
- for $∥{\hat{J}}_{*}-P_{r}^{\prime }J_{*}P_{r}{∥}_{F}$, since $J_{*}=N_{U}\left(I_{r},I_{r}\right)\Theta_{r}\left(I_{r},I_{r}\right)$, we have $∥J_{*}∥\leq N_{U,\max}\theta_{r,\max}\leq \frac{\theta_{r,\max}^{2}\sqrt{K\lambda_{1}\left(\Pi_{r}^{\prime }\Pi_{r}\right)}}{\theta_{r,\min}}$, which gives that $∥J_{*}{∥}_{F}\leq \frac{\theta_{r,\max}^{2}K\sigma_{1}\left(\Pi_{r}\right)}{\theta_{r,\min}}$. Thus, we have $∥{\hat{J}}_{*}-P_{r}^{\prime }J_{*}P_{r}{∥}_{F}=O\left(\frac{\theta_{r,\max}^{2}K\sigma_{1}\left(\Pi_{r}\right)}{\theta_{r,\min}}\right)$.

Combining the above results, we have

$$\begin{array}{cc} & ∥e_{i}^{\prime }\left({\hat{Z}}_{r}-Z_{r}P_{r}\right){∥}_{F}\leq ∥e_{i}^{\prime }\left({\hat{Y}}_{*}-Y_{*}P_{r}\right){∥}_{F}∥{\hat{J}}_{*}{∥}_{F}+∥e_{i}^{\prime }Y_{*}{∥}_{F}{∥{\hat{J}}_{*}-P_{r}^{\prime }J_{*}P_{r}∥}_{F}\\ \; & =O\left(\frac{K^{4.5}\theta_{r,\max}^{14}\varpi \kappa^{4.5}\left(\Pi_{r}^{\prime }\Pi_{r}\right)\lambda_{1}\left(\Pi_{r}^{\prime }\Pi_{r}\right)}{\theta_{r,\min}^{14}\pi_{r,\min}}\right)O\left(\theta_{r,\max}\sqrt{K}\sigma_{1}\left(\Pi_{r}\right)\kappa \left(\Pi_{c}\right)\right)\\ \; & \;\;\;+\frac{\theta_{r,\max}^{2}\sqrt{K\lambda_{1}\left(\Pi_{r}^{\prime }\Pi_{r}\right)}}{\theta_{r,\min}^{2}\lambda_{K}\left(\Pi_{r}\Pi_{r}\right)}O\left(\frac{\theta_{r,\max}^{2}K\sigma_{1}\left(\Pi_{r}\right)}{\theta_{r,\min}}\right)=O\left(\frac{K^{5}\theta_{r,\max}^{15}\varpi \kappa^{4.5}\left(\Pi_{r}^{\prime }\Pi_{r}\right)\kappa \left(\Pi_{c}\right)\lambda_{1}^{1.5}\left(\Pi_{r}^{\prime }\Pi_{r}\right)}{\theta_{r,\min}^{14}\pi_{r,\min}}\right). \end{array}$$

   □

### Appendix E.1. Proof of Theorem 2

**Proof.** We bound $∥e_{j}^{\prime }\left({\hat{\Pi }}_{c}-\Pi_{c}P_{c}\right){∥}_{1}$ first. Recall that $Z_{c}=\Pi_{c},\Pi_{c}\left(j,:\right)=\frac{Z_{c}\left(j,:\right)}{∥Z_{c}{\left(j,:\right)∥}_{1}},{\hat{\Pi }}_{c}\left(i,:\right)=\frac{{\hat{Z}}_{c}\left(j,:\right)}{∥{\hat{Z}}_{c}{\left(j,:\right)∥}_{1}}$, for $1\leq j\leq n_{c}$, since

$$\begin{array}{cc} ∥e_{j}^{\prime }\left({\hat{\Pi }}_{c}-\Pi_{c}P_{c}\right){∥}_{1} & =∥\frac{e_{j}^{\prime }{\hat{Z}}_{c}}{∥e_{j}^{\prime }{\hat{Z}}_{c}{∥}_{1}}-\frac{e_{j}^{\prime }Z_{c}P_{c}}{∥e_{j}^{\prime }Z_{c}P_{c}{∥}_{1}}{∥}_{1}={∥\frac{e_{j}^{\prime }{\hat{Z}}_{c}∥e_{j}^{\prime }Z_{c}{∥}_{1}-e_{j}^{\prime }Z_{c}P_{c}{∥e_{j}^{\prime }{\hat{Z}}_{c}∥}_{1}}{∥e_{j}^{\prime }{\hat{Z}}_{c}{∥}_{1}{∥e_{j}^{\prime }Z_{c}∥}_{1}}∥}_{1}\\ & =∥\frac{e_{j}^{\prime }{\hat{Z}}_{c}∥e_{j}^{\prime }Z_{c}{∥}_{1}-e_{j}^{\prime }{\hat{Z}}_{c}∥e_{j}^{\prime }{\hat{Z}}_{c}{∥}_{1}+e_{j}^{\prime }{\hat{Z}}_{c}∥e_{j}^{\prime }{\hat{Z}}_{c}{∥}_{1}-e_{j}^{\prime }Z_{c}P{∥e_{j}^{\prime }{\hat{Z}}_{c}∥}_{1}}{∥e_{j}^{\prime }{\hat{Z}}_{c}{∥}_{1}{∥e_{j}^{\prime }Z_{c}∥}_{1}}{∥}_{1}\\ \; & \leq \frac{∥e_{j}^{\prime }{\hat{Z}}_{c}∥e_{j}^{\prime }Z_{c}{∥}_{1}-e_{j}^{\prime }{\hat{Z}}_{c}∥e_{j}^{\prime }{\hat{Z}}_{c}{∥}_{1}{∥}_{1}+∥e_{j}^{\prime }{\hat{Z}}_{c}∥e_{j}^{\prime }{\hat{Z}}_{c}{∥}_{1}-e_{j}^{\prime }Z_{c}P_{c}∥e_{j}^{\prime }{\hat{Z}}_{c}{{∥}_{1}∥}_{1}}{∥e_{j}^{\prime }{\hat{Z}}_{c}{∥}_{1}{∥e_{j}^{\prime }Z_{c}∥}_{1}}\\ \; & =\frac{|∥e_{j}^{\prime }Z_{c}{∥}_{1}-∥e_{j}^{\prime }{\hat{Z}}_{c}{∥}_{1}|+∥e_{j}^{\prime }{\hat{Z}}_{c}-e_{j}^{\prime }Z_{c}P_{c}{∥}_{1}}{∥e_{j}^{\prime }Z_{c}{∥}_{1}}\leq \frac{2∥e_{j}^{\prime }\left({\hat{Z}}_{c}-Z_{c}P_{c}\right){∥}_{1}}{∥e_{j}^{\prime }Z_{c}{∥}_{1}}\\ \; & =\frac{2∥e_{j}^{\prime }\left({\hat{Z}}_{c}-Z_{c}P_{c}\right){∥}_{1}}{∥e_{j}^{\prime }\Pi_{c}{∥}_{1}}=2∥e_{j}^{\prime }\left({\hat{Z}}_{c}-Z_{c}P_{c}\right){∥}_{1}\leq 2\sqrt{K}{∥e_{j}^{\prime }\left({\hat{Z}}_{c}-Z_{c}P_{c}\right)∥}_{F}, \end{array}$$

by Lemma A9, we have

$$\begin{array}{c} ∥e_{j}^{\prime }\left({\hat{\Pi }}_{c}-\Pi_{c}P_{c}\right){∥}_{1}=O(\sqrt{K}∥e_{j}^{\prime }\left({\hat{Z}}_{c}-Z_{c}P_{c}\right){∥}_{F})=O\left(\varpi K\kappa \left(\Pi_{c}^{\prime }\Pi_{c}\right)\sqrt{\lambda_{1}\left(\Pi_{c}^{\prime }\Pi_{c}\right)}\right). \end{array}$$

For row nodes $1\leq i\leq n_{r}$, recall that $Z_{r}=Y_{*}J_{*}\equiv N_{U}^{-1}N_{M}\Pi_{r},{\hat{Z}}_{r}={\hat{Y}}_{*}{\hat{J}}_{*},\Pi_{r}\left(i,:\right)=\frac{Z_{r}\left(i,:\right)}{∥Z_{r}{\left(i,:\right)∥}_{1}}$ and ${\hat{\Pi }}_{r}\left(i,:\right)=\frac{{\hat{Z}}_{r}\left(i,:\right)}{∥{\hat{Z}}_{r}{\left(i,:\right)∥}_{1}}$, where $N_{M}$ and *M* are defined in the proof of Lemma 1 such that $U=\Theta_{r}M\equiv \Theta_{r}\Pi_{r}B_{r}$ and $N_{M}\left(i,i\right)=\frac{1}{{∥M\left(i,:\right)∥}_{F}}$, similar as the proof for column nodes, we have

$$\begin{array}{c} ∥e_{i}^{\prime }\left({\hat{\Pi }}_{r}-\Pi_{r}P_{r}\right){∥}_{1}\leq \frac{2∥e_{i}^{\prime }\left({\hat{Z}}_{r}-Z_{r}P_{r}\right){∥}_{1}}{∥e_{i}^{\prime }Z_{r}{∥}_{1}}\leq \frac{2\sqrt{K}{∥e_{i}^{\prime }\left({\hat{Z}}_{r}-Z_{r}P_{r}\right)∥}_{F}}{∥e_{i}^{\prime }Z_{r}{∥}_{1}}. \end{array}$$

Now, we provide a lower bound of $∥e_{i}^{\prime }Z_{r}{∥}_{1}$ as below

$$\begin{array}{cc} ∥e_{i}^{\prime }Z_{r}{∥}_{1} & =∥e_{i}^{\prime }N_{U}^{-1}N_{M}\Pi_{r}{∥}_{1}=∥N_{U}^{-1}\left(i,i\right)e_{i}^{\prime }N_{M}\Pi_{r}{∥}_{1}=N_{U}^{-1}\left(i,i\right){∥N_{M}\left(i,i\right)e_{i}^{\prime }\Pi_{r}∥}_{1}=\frac{N_{M}\left(i,i\right)}{N_{U}\left(i,i\right)}\\ \; & ={∥U\left(i,:\right)∥}_{F}N_{M}\left(i,i\right)={∥U\left(i,:\right)∥}_{F}\frac{1}{{∥M\left(i,:\right)∥}_{F}}={∥U\left(i,:\right)∥}_{F}\frac{1}{∥e_{i}^{\prime }{M∥}_{F}}\\ \; & ={∥U\left(i,:\right)∥}_{F}\frac{1}{∥e_{i}^{\prime }\Theta_{r}^{-1}{U∥}_{F}}={∥U\left(i,:\right)∥}_{F}\frac{1}{∥\Theta_{r}^{-1}\left(i,i\right)e_{i}^{\prime }{U∥}_{F}}=\theta_{r}\left(i\right)\geq \theta_{r,\min}. \end{array}$$

Therefore, by Lemma A9, we have

$$\begin{array}{cc} ∥e_{i}^{\prime }\left({\hat{\Pi }}_{r}-\Pi_{r}P_{r}\right){∥}_{1} & \leq \frac{2\sqrt{K}{∥e_{i}^{\prime }\left({\hat{Z}}_{r}-Z_{r}P_{r}\right)∥}_{F}}{∥e_{i}^{\prime }Z_{r}{∥}_{1}}\leq \frac{2\sqrt{K}{∥e_{i}^{\prime }\left({\hat{Z}}_{r}-Z_{r}P_{r}\right)∥}_{F}}{\theta_{r,\min}}\\ \; & =O(\frac{K^{5.5}\theta_{r,\max}^{15}\varpi \kappa^{4.5}\left(\Pi_{r}^{\prime }\Pi_{r}\right)\kappa \left(\Pi_{c}\right)\lambda_{1}^{1.5}\left(\Pi_{r}^{\prime }\Pi_{r}\right)}{\theta_{r,\min}^{15}\pi_{r,\min}}). \end{array}$$

   □

### Appendix E.2. Proof of Corollary 1

**Proof.** Under conditions of Corollary 1, we have

$$\begin{array}{cc} \; & ∥e_{i}^{\prime }\left({\hat{\Pi }}_{r}-\Pi_{r}P_{r}\right){∥}_{1}=O\left(\frac{K^{5.5}\theta_{r,\max}^{15}\varpi \kappa^{4.5}\left(\Pi_{r}^{\prime }\Pi_{r}\right)\kappa \left(\Pi_{c}\right)\lambda_{1}^{1.5}\left(\Pi_{r}^{\prime }\Pi_{r}\right)}{\theta_{r,\min}^{15}\pi_{r,\min}}\right)=O\left(\frac{\theta_{r,\max}^{15}\varpi \sqrt{n_{r}}}{\theta_{r,\min}^{15}}\right),\\ \; & ∥e_{j}^{\prime }\left({\hat{\Pi }}_{c}-\Pi_{c}P_{c}\right){∥}_{1}=O\left(\varpi K\kappa \left(\Pi_{c}^{\prime }\Pi_{c}\right)\sqrt{\lambda_{1}\left(\Pi_{c}^{\prime }\Pi_{c}\right)}\right)=O\left(\varpi \sqrt{n_{c}}\right). \end{array}$$

Under conditions of Corollary 1, Lemma A7 gives $\varpi =O(\frac{\sqrt{P_{\max}\theta_{r,\max}\log\left(n_{r}+n_{c}\right)}}{\theta_{r,\min}\sigma_{K}\left(P\right)\sqrt{n_{r}n_{c}}})$, which gives that

$$\begin{array}{cc} \; & ∥e_{i}^{\prime }\left({\hat{\Pi }}_{r}-\Pi_{r}P_{r}\right){∥}_{1}=O\left(\frac{\theta_{r,\max}^{15}\varpi \sqrt{n_{r}}}{\theta_{r,\min}^{15}}\right)=O\left(\frac{\theta_{r,\max}^{15.5}\sqrt{P_{\max}\log\left(n_{r}+n_{c}\right)}}{\theta_{r,\min}^{16}\sigma_{K}\left(P\right)\sqrt{n_{c}}}\right),\\ \; & ∥e_{j}^{\prime }\left({\hat{\Pi }}_{c}-\Pi_{c}P_{c}\right){∥}_{1}=O\left(\varpi \sqrt{n_{c}}\right)=O\left(\frac{\sqrt{P_{\max}\theta_{r,\max}\log\left(n_{r}+n_{c}\right)}}{\theta_{r,\min}\sigma_{K}\left(P\right)\sqrt{n_{r}}}\right). \end{array}$$

By basic algebra, this corollary follows.    □

## Appendix F. SVM-Cone Algorithm

For readers’ convenience, we briefly introduce the SVM-cone algorithm given in [26] and provide another view that the SVM-cone algorithm exactly recovers $\Pi_{r}$ when the input is $U_{*}$ (or $U_{*,2}$). Let *S* be a matrix whose rows have unit $l_{2}$ norm, and *S* can be written as $S=HS_{C}$, where $H\in R^{n\times K}$ with nonnegative entries, no row of *H* is 0, and $S_{C}\in R^{K\times m}$ corresponding to *K* rows of *S* (i.e., there exists an index set I with *K* entries such that $S_{C}=S\left(I,:\right)$). Inferring *H* from *S* is called the ideal cone problem, i.e., Problem 1 in [26]. The ideal cone problem can be solved by applying one-class SVM to the rows of *S*, and the *K* rows of $S_{C}$ are the support vectors found by one-class SVM:

(A5) $$\begin{array}{c} \mathrm{maximize}\;b\;\;s.t.\;\;w^{\prime }S\left(i,:\right)\geq b\left(\;\mathrm{for}\;i=1,2,\ldots ,n\right)\;\mathrm{and}\;\;{∥w∥}_{F}\leq 1. \end{array}$$

The solution (w,b) for the ideal cone problem when ${\left(S_{C}S_{C}^{\prime }\right)}^{-1}1>0$ is given by

(A6) $$\begin{array}{c} w=b^{-1}\cdot S_{C}^{\prime }\frac{{\left(S_{C}S_{C}^{\prime }\right)}^{-1}1}{1^{\prime }{\left(S_{C}S_{C}^{\prime }\right)}^{-1}1},\;\;\;b=\frac{1}{\sqrt{1^{\prime }{\left(S_{C}S_{C}^{\prime }\right)}^{-1}1}}. \end{array}$$

For the empirical case, let $\hat{S}\in R^{n\times m}$ be a matrix where all rows have unit $l_{2}$ norm, infer *H* from $\hat{S}$ with given *K* is called the empirical cone problem, i.e., Problem 2 in [26]. For the empirical cone problem, one-class SVM is applied to all rows of $\hat{S}$ to obtain w and *b*’s estimations $\hat{w}$ and $\hat{b}$. Then, apply the K-means algorithm to rows of $\hat{S}$ that are close to the hyperplane into *K* clusters, and an estimation of the index set I can be obtained from the *K* clusters provided. Algorithm A3 below is the SVM-cone algorithm provided in [26].

**Algorithm 3:** SVM-cone [26]
Require: $\hat{S}\in R^{n\times m}$ with rows having unit $l_{2}$ norm, number of corners *K*, estimated distance corners from hyperplane γ. Ensure: The near-corner index set $\hat{I}$. 1: Run one-class SVM on $\hat{S}\left(i,:\right)$ to obtain $\hat{w}$ and $\hat{b}$. 2: Run K-means algorithm to the set $\left\{\hat{S}\left(i,:\right)|\hat{S}\left(i,:\right)\hat{w}\leq \hat{b}+\gamma \right\}$ that are close to the hyperplane into *K* clusters. 3: Pick one point from each cluster to obtain the near-corner set $\hat{I}$.

As suggested in [26], we can start γ=0 and incrementally increase it until *K* distinct clusters are found.

Now, turn to our DiMSC algorithm, and focus on estimating $I_{r}$ with given $U_{*},U_{*,2}$, and *K*. By Lemmas 1 and A1, we know that $U_{*}$ and $U_{*,2}$ enjoy the ideal cone structure, and Lemma 2 guarantees that one-class SVM can be applied to rows of $U_{*}$ and $U_{*,2}$. Set $w_{1}=b_{1}^{-1}U_{*}^{\prime }\left(I_{r},:\right)\frac{{\left(U_{*}\left(I_{r},:\right)U_{*}^{\prime }\left(I_{r},:\right)\right)}^{-1}1}{1^{\prime }{\left(U_{*}\left(I_{r},:\right)U_{*}^{\prime }\left(I_{r},:\right)\right)}^{-1}},b_{1}=\frac{1}{\sqrt{1^{\prime }{\left(U_{*}\left(I_{r},:\right)U_{*}^{\prime }\left(I_{r},:\right)\right)}^{-1}1}}$, and $w_{2}=b_{2}^{-1}U_{*,2}^{\prime }\left(I_{r},:\right)\frac{{\left(U_{*,2}\left(I_{r},:\right)U_{*,2}^{\prime }\left(I_{r},:\right)\right)}^{-1}1}{1^{\prime }{\left(U_{*,2}\left(I_{r},:\right)U_{*,2}^{\prime }\left(I_{r},:\right)\right)}^{-1}},b_{2}=\frac{1}{\sqrt{1^{\prime }{\left(U_{*,2}\left(I_{r},:\right)U_{*,2}^{\prime }\left(I_{r},:\right)\right)}^{-1}1}}$. Now that $w_{1}$ and $b_{1}$ are solutions of the one-class SVM in Equation (A5) by setting $S=U_{*}$, and $w_{2}$ and $b_{2}$ are solutions of the one-class SVM in Equation (A5) by setting $S=U_{*,2}$. Lemma A11 says that if row node *i* is a pure node, we have $U_{*}\left(i,:\right)w_{1}=b_{1}$, and this suggests that in the SVM-cone algorithm, if the input matrix is $U_{*}$, by setting γ=0, we can find all pure row nodes, i.e., the set $\left\{U_{*}\left(i,:\right)|U_{*}\left(i,:\right)w_{1}=b_{1}\right\}$ contains all rows of $U_{*}$ respective to pure row nodes while including mixed row nodes. By Lemma 1, these pure row nodes belong to the *K* distinct row communities such that if row nodes $i,\bar{i}$ are in the same row community, then we have $U_{*}\left(i,:\right)=U_{*}\left(\bar{i},:\right)$, and this is the reason that we need to apply the K-means algorithm on the set obtained in Step 2 in the SVM-cone algorithm to obtain the *K* distinct row communities, and this is also the reason that we said the SVM-cone algorithm returns the index set I exactly when the input is $U_{*}$. These conclusions also hold when we set the input in the SVM-cone algorithm as $U_{*,2}$.

**Lemma A10.** 
*Under $DiDCMM_{n_{r},n_{c}}\left(K,P,\Pi_{r},\Pi_{c},\Theta_{r}\right)$, for $1\leq i\leq n_{r}$, $U_{*}\left(i,:\right)$ can be written as $U_{*}\left(i,:\right)=r_{1}\left(i\right)\Phi_{1}\left(i,:\right)U_{*}\left(I_{r},:\right)$, where $r_{1}\left(i\right)\geq 1$. Meanwhile, $r_{1}\left(i\right)=1$ and $\Phi_{1}\left(i,:\right)=e_{k}^{\prime }$ if i is a pure node such that $\Pi_{r}\left(i,k\right)=1$; $r_{1}\left(i\right)>1$ and $\Phi_{1}\left(i,:\right)\neq e_{k}^{\prime }$ if $\Pi_{r}\left(i,k\right)<1$ for 1≤k≤K. Similarly, $U_{*,2}\left(i,:\right)$ can be written as $U_{*,2}\left(i,:\right)=r_{2}\left(i\right)\Phi_{2}\left(i,:\right)U_{*,2}\left(I_{r},:\right)$, where $r_{2}\left(i\right)\geq 1$. Meanwhile, $r_{2}\left(i\right)=1$ and $\Phi_{2}\left(i,:\right)=e_{k}^{\prime }$ if $\Pi_{r}\left(i,k\right)=1$; $r_{2}\left(i\right)>1$ and $\Phi_{2}\left(i,:\right)\neq e_{k}^{\prime }$ if $\Pi_{r}\left(i,k\right)<1$ for 1≤k≤K.*

**Proof.** Since $U_{*}=YU_{*}\left(I_{r},:\right)$ by Lemma 1, for $1\leq i\leq n_{r}$, we have

$$\begin{array}{c} U_{*}\left(i,:\right)=Y\left(i,:\right)U_{*}\left(I_{r},:\right)=Y\left(i,:\right)1\frac{Y(i,:)}{Y(i,:)1}U_{*}\left(I_{r},:\right)=r_{1}\left(i\right)\Phi_{1}\left(i,:\right)U_{*}\left(I_{r},:\right), \end{array}$$

where we set $r_{1}\left(i\right)=Y\left(i,:\right)1$, $\Phi_{1}\left(i,:\right)=\frac{Y(i,:)}{Y(i,:)1}$, and 1 is a K×1 vector with all entries being ones. By the proof of Lemma 1, $Y\left(i,:\right)=\frac{\Pi_{r}\left(i,:\right)}{{∥M\left(i,:\right)∥}_{F}}\Theta_{r}^{-1}\left(I_{r},I_{r}\right)N_{U}^{-1}\left(I_{r},I_{r}\right)$, where $M=\Pi_{r}\Theta_{r}^{-1}\left(I_{r},I_{r}\right)U\left(I_{r},:\right)$. For convenience, set $T=\Theta_{r}^{-1}\left(I_{r},I_{r}\right),Q=N_{U}^{-1}\left(I_{r},I_{r}\right)$, and $R=U(I_{r},:)$ (such setting of T,Q,R is only for used for notation convenience for the proof of Lemma A10). On the one hand, if row node *i* is pure such that $\Pi_{r}\left(i,k\right)=1$ for certain *k* among {1,2,…,K} (i.e., $\Pi_{r}\left(i,:\right)=e_{k}$ if $\Pi_{r}\left(i,k\right)=1$), we have $M\left(i,:\right)=\Pi_{r}\left(i,:\right)\Theta_{r}^{-1}\left(I_{r},I_{r}\right)U\left(I_{r},:\right)=T\left(k,k\right)R\left(k,:\right)$, and $\Pi_{r}\left(i,:\right)TQ=T\left(k,k\right)Q\left(k,:\right)$, which give that $Y\left(i,:\right)=\frac{T(k,k)Q(k,:)}{{∥T\left(k,k\right)R\left(k,:\right)∥}_{F}}=\frac{Q(k,:)}{{∥R\left(k,:\right)∥}_{F}}$. Recall that the *k*-th diagonal entry of $N_{U}^{-1}\left(I_{r},I_{r}\right)$ is $∥\left[U\left(I_{r},:\right)\right]\left(k,:\right){∥}_{F}$, i.e., $Q\left(k,:\right)1={∥R\left(k,:\right)∥}_{F}$, which gives that $r_{1}\left(i\right)=Y\left(i,:\right)1=1$ and $\Phi_{1}\left(i,:\right)=e_{k}^{\prime }$ when $\Pi_{r}\left(i,k\right)=1$. On the other hand, if *i* is a mixed node, since ${∥M\left(i,:\right)∥}_{F}=∥\Pi_{r}\left(i,:\right)\Theta_{r}^{-1}\left(I_{r},I_{r}\right)U\left(I_{r},:\right){∥}_{F}=∥\sum_{k=1}^{K}\Pi_{r}\left(i,k\right)T\left(k,k\right)R\left(k,:\right){∥}_{F}<\sum_{k=1}^{K}\Pi_{r}\left(i,k\right)T\left(k,k\right){∥R\left(k,:\right)∥}_{F}=\sum_{k=1}^{K}\Pi_{r}\left(i,k\right)T\left(k,k\right)Q\left(k,k\right)$, combine it with $\Pi_{r}\left(i,:\right)TQ1=\sum_{k=1}^{K}\Pi_{r}\left(i,k\right)T\left(k,k\right)Q\left(k,k\right)$, so $r_{1}\left(i\right)=Y\left(i,:\right)1=\frac{\Pi_{r}\left(i,:\right)TQ1}{{∥M\left(i,:\right)∥}_{F}}>1$. The lemma follows by a similar analysis for $U_{*,2}$. □

**Lemma A11.** 
*Under $DiDCMM_{n_{r},n_{c}}\left(K,P,\Pi_{r},\Pi_{c},\Theta_{r}\right)$, for $1\leq i\leq n_{r}$, if row node i is a pure node such that $\Pi_{r}\left(i,k\right)=1$ for certain k, we have*

$$\begin{array}{c} U_{*}\left(i,:\right)w_{1}=b_{1}\;\;\;\mathrm{and}\;\;\;U_{*,2}\left(i,:\right)w_{2}=b_{2}, \end{array}$$

*Meanwhile, if row node i is a mixed node, the above equalities do not hold.*

**Proof.** For the claim that $U_{*}\left(i,:\right)w_{1}=b_{1}$ holds when *i* is pure, by Lemma A10, when *i* is a pure node such that $\Pi_{r}\left(i,k\right)=1$, $U_{*}\left(i,:\right)$ can be written as $U_{*}\left(i,:\right)=e_{k}^{\prime }U_{*}\left(I_{r},:\right)$, so $U_{*}\left(i,:\right)w_{1}=b_{1}$ holds surely. When *i* is a mixed node, by Lemma A10, $r_{1}\left(i\right)>1$ and $\Phi_{1}\left(i,:\right)\neq e_{k}$ for any k=1,2,…,K; hence $U_{*}\left(i,:\right)\neq e_{k}^{\prime }U_{*}\left(I_{r},:\right)$ if *i* is mixed, which gives the result. Follow a similar analysis, we obtain the results associated with $U_{*,2}$, and the lemma follows. □
